# Coupled spin-coordination self-adaptation drives efficient nitrate to ammonia conversion

**DOI:** 10.1038/s41467-026-77163-2

**Published:** 2026-08-29

**Authors:** Chun-Kuo Peng, Yu-Ru Lin, Hsiang-Chun Yu, Shih-Ching Huang, Yu-Chang Lin, Daqin Guan, Xiaomin Xu, Yingtang Zhou, Yung-Jung Hsu, San-Yuan Chen, Zongping Shao, Yan-Gu Lin

**Affiliations:** 1 Curtin Centre for Advanced Energy Materials and Technologies (CAEMT), WA School of Mines (WASM), Curtin University, Perth, WA Australia; 2 Scientific Research Division, National Synchrotron Radiation Research Center, Hsinchu, Taiwan; 3 Zhejiang Key Laboratory of Pollution Control for Port-Petrochemical Industry, Marine Science and Technology College, Zhejiang Ocean University, Zhoushan, China; 4 Department of Materials Science and Engineering, National Yang Ming Chiao Tung University, Hsinchu, Taiwan; 5 Graduate Institute of Biomedical Sciences, China Medical University, Taichung, Taiwan; 6 School of Dentistry, College of Dental Medicine, Kaohsiung Medical University, Kaohsiung, Taiwan

**Keywords:** Electrocatalysis, Electrocatalysis, Electrocatalysis

## Abstract

Electrochemical nitrate reduction reaction (NO_3_RR) is a promising approach for sustainable ammonia production and nitrogen pollution mitigation, but its efficiency is often limited by sluggish nitrate hydrogenation. The spin state and coordination environment of the active metal play crucial roles in selectivity and reaction kinetics, yet designing electronically adaptive active sites for the complex multielectron NO_3_RR process remains challenging. Here, we report an Fe-N_3_(PO_X_) single-atom catalyst that breaks the local symmetry to induce a switch-like adaptive coordination and dynamical tuning of the Fe spin state, enabling high activity at low overpotentials, with an ammonia yield rate of 21.96 g mg_Fe_^−1^ h^−1^ and a Faradaic efficiency of 89.8% at −0.56 V versus the reversible hydrogen electrode (RHE). In situ spectroscopy confirms coordination reorganization and an increase in the Fe spin state, accompanied by accelerated consumption of asymmetric nitrate species. The resulting higher spin state character enhances back-donation into nitrate-derived intermediates and accelerates hydrogenation. This study demonstrates a viable strategy to manipulate spin states at the single-atom level, providing mechanistic insight for optimizing ammonia production on Fe-based catalysts.

## Abstract (web summary)

Producing ammonia from nitrate could help address both chemical manufacturing and water pollution. Here, the authors show that an iron single-atom catalyst dynamically adjusts its coordination and spin state to promote nitrate conversion to ammonia.

## Introduction

Spin-state regulation has been demonstrated to be an effective way to enhance the intrinsic activity of catalytic sites in many key electrocatalytic reactions, thereby driving catalysts development^1–3^. The rapid conversion of nitrate to ammonia relies on efficient ${{{{\rm{NO}}}}}_{3}^{-}$ adsorption followed by a series of subsequent hydrogenation steps. Although strengthening these steps can increase the overall reaction rate, selectively accelerating those hydrogenation steps that are coupled to spin transitions through spin state control remains highly challenging^4–6^. Ammonia is the cornerstone feedstock of the fertilizer industry and a highly promising carbon-free energy carrier, and is therefore crucial for both global food security and the energy transition^7,8^. Selective conversion of ${{{{\rm{NO}}}}}_{3}^{-}$ to NH_3_ offers a feasible pathway to address energy and environmental issues simultaneously^9^. However, because the nitrate reduction reaction (NO_3_RR) involves an eight-electron transfer, its reaction kinetics are intrinsically sluggish and are further complicated by the hydrogen evolution reaction and various side pathways^10–13^. Consequently, precisely regulating catalyst dynamics under practical operating conditions and elucidating the associated reaction mechanism are of paramount importance.

Conventional metal-N_4_ single-atom catalysts (SACs) combine high atomic utilization with well-defined structures^14–16^, providing an ideal platform for probing fundamental catalytic mechanisms^17,18^. Nevertheless, their metal centers typically adopt a planar D_4_h symmetry with a symmetric charge distribution. Such highly symmetric structures substantially limit the fine-tuning of electronic configurations and hinder controllable adsorption of intermediates along the axial direction^19,20^. Together with the inherent difficulty of systematically tuning the local coordination environment in SACs, this significantly restricts deeper optimization of catalytic activity and selectivity. Accordingly, precisely regulating the adsorption energy of key intermediates at the metal center and enhancing the adsorption and activation of oxygen-containing species are widely regarded as key strategies to improve catalytic performance, steer reaction pathways, and boost selectivity toward the desired products. These considerations highlight an urgent need to develop catalytic platforms that simultaneously offer high activity, high selectivity, and precisely tunable coordination environments.

Fe-based materials possess earth abundance, diverse and tunable electronic structures, and engineerable spin degrees of freedom^2,21,22^, making them strong candidates for designing efficient NO_3_RR catalysts. However, the overall NO_3_RR performance of Fe-based SACs remains far from ideal. In many Fe-based SACs systems, the adsorption and conversion of nitrate-derived intermediates are still not optimally balanced, and hydrogenation-related steps, such as NO* reduction to HNO* and HNO* reduction to N*, have been identified as key kinetic issues^23,24^. Compared with Cu-based catalysts, which are often effective for nitrate adsorption and N-O bond activation^25^, and Co-based catalysts^26,27^, which have been associated with promoting the H* pathway, Fe SACs still require careful regulation of nitrate/NOx adsorption, active hydrogen utilization, and HER suppression. Although recent efforts have explored heteroatom doping to modulate the electronic structure of asymmetric Fe active centers^28^, and even employed external magnetic fields to manipulate the spin state^2^, it remains highly challenging to construct catalytic architectures that can provide dynamic electronic and structural adaptivity in a complex reaction such as NO_3_RR, which involves multiple intermediates and multi-electron transfer steps. At the same time, our understanding of the nature of the active sites, catalytic behavior, and spin state dynamics of Fe SACs is still incomplete. In particular, within the Fe SAC framework, the intrinsic correlation between dynamic electronic/atomic structural evolution and the adsorption behavior of nitrate intermediates has not yet been clearly established. Therefore, developing a design strategy that can drive spontaneous and adaptive spin regulation while unambiguously identifying the true active sites has become a central challenge and is expected to provide a critical route to enhancing the NO_3_RR performance of Fe-based SACs.

In this work, we break the Fe-N_4_ symmetry and design Fe-N_3_(PO_X_) SACs with an asymmetric coordination environment. Under working conditions, these Fe-N_3_(PO_X_) sites can spontaneously adjust their local coordination structure and spin state as a function of potential and intermediate coverage, exhibiting a highly coupled, electronically and structurally adaptive character. The asymmetric coordination induced by PO_X_ moderately weakens the bonding strength of oxygenated intermediates in the initial state, while under operating potentials, it endows the active site with a tunable, switch-like catalytic behavior that facilitates activation to a highly active state at relatively low potentials. Fe-N_3_(PO_X_) achieves 21.96 g mg_Fe_^−1^ h^−1^ at − 0.56 V, reaching a gram-level ammonia production rate. In situ X-ray absorption and emission spectroscopy (XAS/XES) reveal pronounced local structural reorganization and a spin state increase at the Fe center during NO_3_RR, whereas in situ ATR-FTIR reflectance spectroscopy shows that the PO_X_-induced asymmetric sites preferentially activate and consume asymmetrically coordinated ${{{{\rm{NO}}}}}_{3}^{-}$ species. Taken together, these results demonstrate that Fe-N_3_(PO_X_) SACs realize cooperative regulation of coordination structure and spin state, establish a clear link between coordination environment, spin state, and catalytic performance, and uncover the true active centers and reaction dynamics at asymmetric Fe single-atom sites, thereby providing concrete and broadly applicable design guidelines for the rational development of highly efficient NO_3_RR electrocatalysts.

## Results

### Structure characterization of Fe-N_3_(PO_X_)

**Fig. 1:**
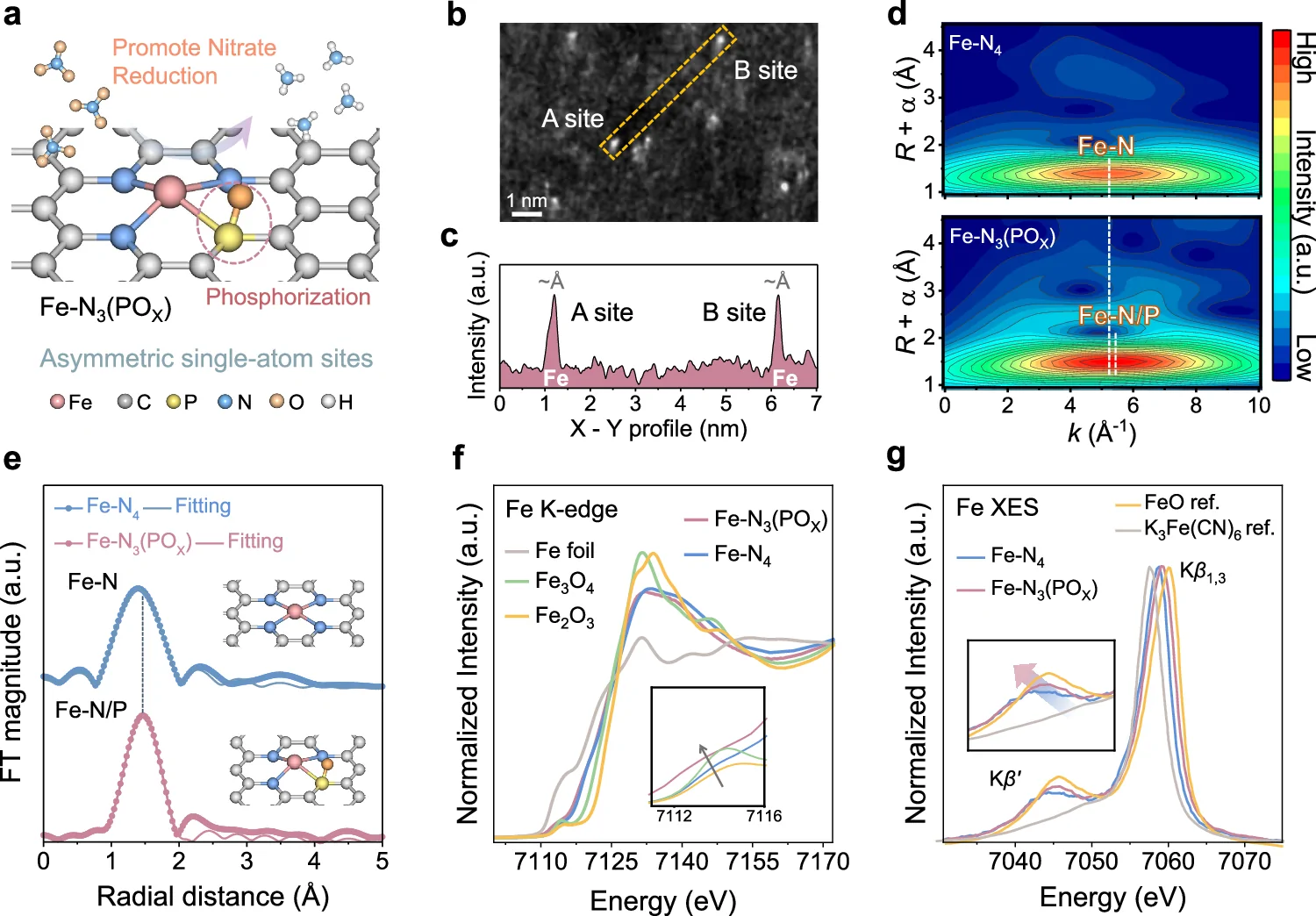
Characterization of Fe-N_3_(PO_X_) catalysts. **a** Schematic illustration of Fe-N_3_(PO_X_) for enhanced NO_3_RR. **b** TEM image of Fe-N_3_(PO_X_) and (**c**) the corresponding intensity profiles. **d** WT-EXAFS contour plot for Fe-N_4_ and Fe-N_3_(PO_X_). **e** *k*^3^-weighted EXAFS fitting curves of Fe-N_4_ and Fe-N_3_(PO_X_). Insets show the corresponding Fe-N_4_ and Fe-N_3_(PO_X_) structural models used for the EXAFS fitting. **f** Fe K-edge XANES spectra for Fe-N_4_ and Fe-N_3_(PO_X_) and references. **g** XES spectra of Fe-N_4_ and Fe-N_3_(PO_X_) and references. Source data are provided as a Source Data file.

Fe-N_4_ single-atom catalysts supported on porous carbon were synthesized using an aqueous Fe precursor with glucose and NaCl as templates. Subsequent phosphorylation yielded Fe-N_3_(PO_X_) single-atom sites (Fig. 1a). 3D transmission electron microscope (TEM) image plots of Fe-N_3_(PO_X_) in Fig. 1b clearly exhibit each sharp peak of these atomic sites, confirming that single-atom sites are uniformly dispersed. Figure 1c further shows a characteristic size around Å for these atomic sites. As observed across a wider field of view in the high-resolution TEM (HR-TEM) image of Fe-N_4_ and Fe-N_3_(PO_X_) (Supplementary Fig. 3a, b), all Fe single-atom sites are uniformly dispersed as atomically bright spots without aggregation, even after phosphorylation. High-resolution powder X-ray diffraction (HR-PXRD) in Supplementary Fig. 2 shows only broad graphitic features and no reflections from metallic or oxide phases, supporting atomically dispersed Fe in both materials. Inductively Coupled Plasma Optical Emission Spectrometer (ICP-OES) shows Fe contents of 0.314% for Fe-N_4_ and 0.215% for Fe-N_3_(PO_X_), and a P content of 7.25% for Fe-N_3_(PO_X_) (Supplementary Table 1). This indicates that introducing PO_X_ does not change the Fe loading or dispersion. To better elucidate the local structure around the Fe single-atom sites upon PO_X_ incorporation, XAS can be effectively employed for detailed analysis. In k-space, both samples show simple χ(k) oscillations arising only from first-shell Fe-N or Fe-N/PO_X_ scattering for Fe-N_4_ and Fe-N_3_(PO_X_), unlike the more complex features in references, indicating the formation of isolated single-atom Fe sites (Supplementary Fig. 3). As shown in Fig. 1d, wavelet transform-extended X-ray absorption fine structure (WT-EXAFS) at Fe K-edge reveals that the dominant feature of Fe-N_3_(PO_X_) shifts to higher k relative to Fe-N_4_, indicating PO_X_ as a heavier backscattered path that partially replaces N in the first coordination shell and confirming the presence of Fe-PO_X_ pairs. Fourier transform EXAFS fitting quantifies PO_X_ -induced coordination changes. An Fe foil reference was fitted to determine the amplitude reduction factor (Supplementary Fig. 4). As shown in Fig. 1e, only a pronounced first-shell contribution is observed, and the fits reproduce the spectra well. As summarized in Supplementary Table 2, the pristine Fe-N_4_ site is coordinated by four nitrogen atoms at an Fe-N distance of 1.82 Å. Upon PO_X_ incorporation, the Fe-N_3_(PO_X_) configuration contains a single Fe-P(O_X_) path at 2.18 Å that partially replaces the Fe-N_4_ coordination. Meanwhile, the Fe-N in Fe-N_3_(PO_X_) coordination number decreases from 4 to 3, and the Fe-N bond length decreases to 1.81 Å relative to Fe-N_4_. The incorporation of a longer Fe-PO_X_ coordination drives an outward desymmetrization of the local Fe environment (see inset of Fig. 1e). Subsequent analysis determines PO_X_ speciation and quantifies its influence on the Fe electronic structure in Fe-N_3_(PO_X_). X-ray absorption near-edge structure (XANES) was used to probe the electronic structure (Fig. 1f). The absorption edge of Fe-N_3_(PO_X_) is slightly shifted to lower energy relative to Fe-N_4_, indicating a higher electron density at the Fe center. Linear calibration of the edge position (Supplementary Fig. 5) yields average oxidation states of + 2.7 for Fe-N_4_ and + 2.5 for Fe-N_3_(PO_X_). The lower oxidation state of Fe-N_3_(PO_X_) is consistent with stronger electron donation from PO_X_ than from N due to its lower electronegativity^29^. Notably, the pre-edge feature of Fe-N_3_(PO_X_) increases in intensity (inset of Fig. 1f), indicating reduced local symmetry^30,31^. This asymmetry arises from coordination-geometry distortion introduced by the longer Fe-PO_X_ bond, consistent with the EXAFS analysis above. To clarify ligand speciation, X-ray Photoelectron Spectroscopy (XPS) and soft X-ray absorption spectroscopy (soft XAS) were carried out. The N 1 *s* spectra of Fe-N_4_ and Fe-N_3_(PO_X_) in Supplementary Fig. 6a, b can be deconvoluted into five components, including pyridinic N, Metallic N, pyrrolic N, graphitic N and oxidized N. The fitted fractions are summarized in Supplementary Table 3. Compared with Fe-N_4_, Fe-N_3_(PO_X_) shows reduced pyrrolic N and metallic N, consistent with partial substitution of pyrrolic N by PO_X_. This observation also indicates that pyrrolic N serves as the anchoring ligand, establishing the Fe-N coordination and fixing the Fe single-atom site. Consistent with the above results, N K-edge soft XAS (Supplementary Fig. 7a) shows that the pyrrolic feature at around 399 eV is markedly weakened after phosphorylation. The P K-edge spectrum of Fe-N_3_(PO_X_) (Fig. 1g) resembles that of FePO_4_, and no feature attributable to bidentate phosphate coordination to Fe is observed near ~2149 eV, indicating that PO_X_ coordinates to Fe predominantly in a monodentate manner^32^. Furthermore, the P 2*p* spectrum in Supplementary Fig. 7b exhibits only phosphate-related features without any signal of phosphide bonding, while a peak assignable to Fe-PO_X_ appears at around 134 eV, confirming that phosphorus is present as PO_X_-type ligands coordinated to Fe^33^. Overall, phosphorylation promotes the formation of PO_X_ ligands bound to Fe, lowers the local structural symmetry, and modifies the local electronic environment of Fe.

XES was used to resolve in detail the spin information and electronic configuration of Fe in the asymmetric Fe-N_3_(PO_X_) site. In Fig. 1h, the K*β*_1,3_ energy position reflects the oxidation state, while the K*β*_1,3_-K*β*′ energy splitting and the K*β*′ intensity are sensitive to the number of unpaired electrons. Compared with Fe-N_4_, Fe-N_3_(PO_X_) shows a higher K*β*_1,3_ peak position, indicating a reduced oxidation state due to the stronger electron-donating effect of PO_X_ ligation, consistent with XANES. Notably, Fe-N_3_(PO_X_) exhibits a stronger K*β*′ feature, indicating that electrons donated by PO_X_ to Fe populate higher-energy levels as unpaired electrons, resulting in more unpaired electrons and a higher spin state character. To further quantify the spin-state variation, the Fe K*β* XES spectra were analyzed by Gaussian fitting. The K*β* emission profiles were deconvoluted into the main K*β*_1,3_ emission line and the K*β*′ satellite feature, and the relative K*β*′ peak areas were extracted for spin-state evaluation (Supplementary Fig. 24a–e and Supplementary Table 7). Based on the reference compounds, a calibration relationship between the relative K*β*′ peak area and the average spin state was established^34,35^ (Supplementary Fig. 25). According to this calibration, the estimated average spin state increases from 1.51 for Fe-N_4_ to 1.82 for Fe-N_3_(PO_X_), indicating that the asymmetric PO_X_ coordination induces a higher-spin character at the Fe center. These results confirm that the Fe-N_3_(PO_X_) configuration promotes spin-state modulation of the Fe single-atom site.

### Electrocatalytic performance toward nitrate reduction

**Fig. 2:**
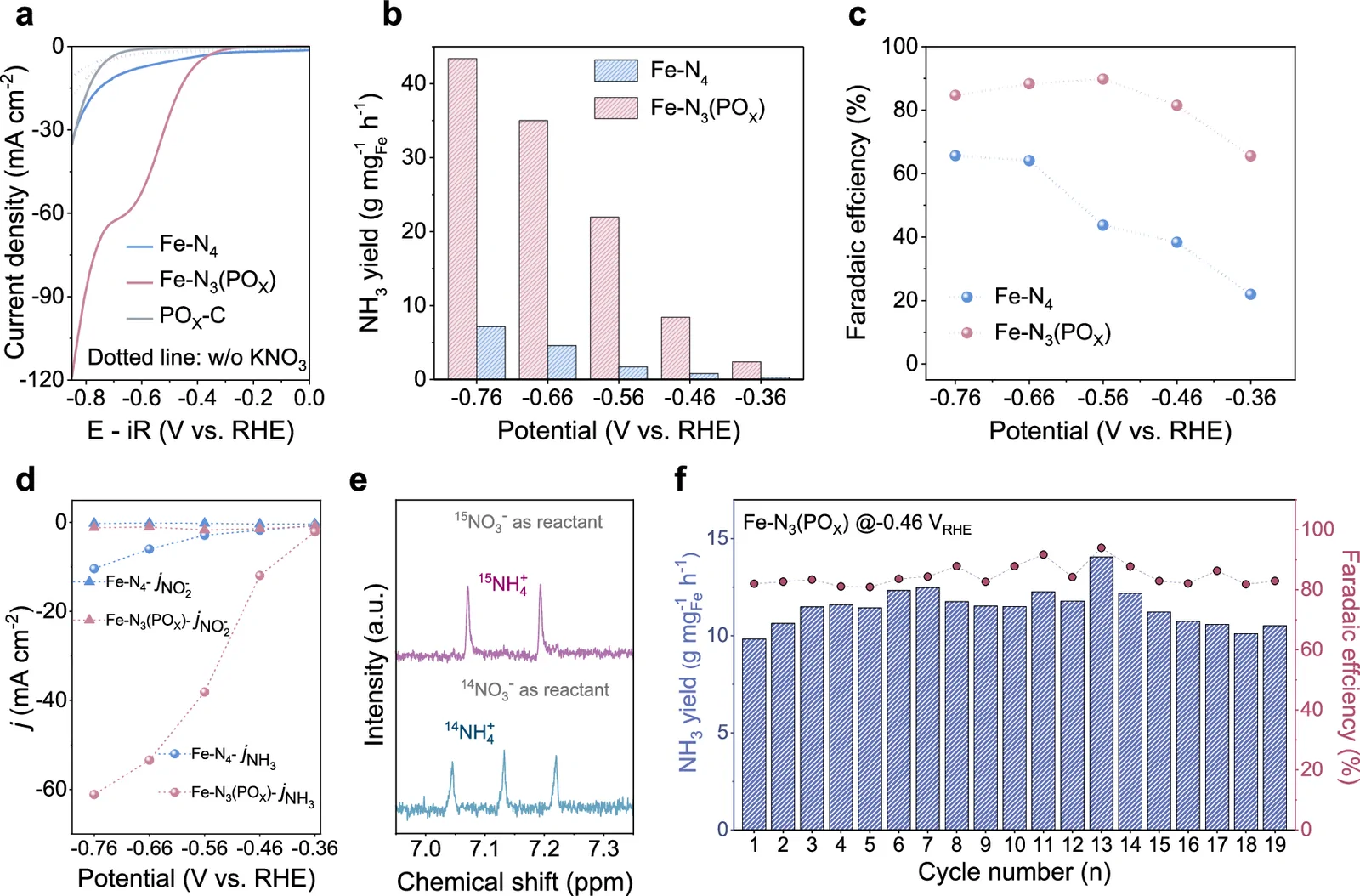
Electrocatalytic nitrate reduction performance. **a** Linear sweep voltammetry curve of Fe-N_4_, Fe-N_3_(PO_X_) and PO_X_-C in 1.0 M KOH without and with 0.1 M KNO_3_ mixed electrolyte (with iR correction). The solution resistances of Fe-N_4_, Fe-N_3_(PO_X_) and PO_X_-C were 2.7 ± 0.02, 2.3 ± 0.05 and 2.4 ± 0.1 Ω in KNO_3_-containing electrolyte. In the KNO_3_-free electrolyte, the values for Fe-N_4_ and Fe-N_3_(PO_X_) were 2.7 ± 0.01 and 2.9 ± 0.02 Ω, respectively. **b** Ammonia yield rate and (**c**) Faradaic efficiency at different applied potentials for Fe-N_4_ and Fe-N_3_(PO_X_), (potentials are not iR corrected). **d** Potential-dependent partial current density of NH_3_ and ${{{{\rm{NO}}}}}_{2}^{-}$ for Fe-N_4_ and Fe-N_3_(PO_X_) with iR correction. **e** ^1^H NMR spectra of ^14^NH_4_^+^ and ^15^NH_4_^+^ produced after the NO_3_RR at −0.46 V for 2 h using ^14^NO_3_ and ^15^NO_3_ as reactants for Fe-N_4_. **f** Cycling tests of Fe-N_3_(PO_X_) at −0.46 V during 19 periods of electrocatalytic NO_3_RR (potentials are not iR corrected). Electrochemical measurements were performed in a two-compartment H-cell at 25 °C. Source data are provided as a Source Data file.

Figure 2a compares the nitrate reduction performance of Fe-N_4_, Fe-N_3_(PO_X_) and PO_X_-doped carbon (PO_X_-C) measured in 1.0 M KOH and in a mixed electrolyte with 0.1 M KNO_3_, both with iR correction. The corresponding non-iR corrected LSV curves are shown in Supplementary Fig. 9a. The Hg/HgO reference electrode was calibrated against RHE using a Pt foil electrode in H_2_ saturated 1.0 M KOH electrolyte, as shown in Supplementary Fig. 9b. A PO_X_-C blank control was tested under NO_3_RR conditions. Its current density was much lower than those of Fe-N_4_ and Fe-N_3_(PO_X_), and the corresponding indophenol assay showed almost no detectable NH_3_ production, confirming that PO_X_-C itself contributes negligibly to NO_3_RR activity. The dashed line corresponds to measurements in pure 1.0 M KOH. In 1.0 M KOH + 0.1 M KNO_3_, the Fe-N_3_(PO_X_) SACs shows a much lower onset potential, shifted positively by about 0.4 V relative to Fe-N_4_, and the current density at each potential is more than four times that of pristine Fe-N_4_. This indicates that Fe-N_3_(PO_X_) enables fast NO_3_RR. Figure 2b presents bar charts of NH_3_ yield at each potential for Fe-N_4_ and Fe-N_3_(PO_X_). The 0.25 h potentiostatic i-t results in 1.0 M KOH + 0.1 M KNO_3_ are shown in Supplementary Fig. 9c, d. Ammonia in the electrolyte was quantified by the UV-vis indophenol method, and the calibration in Supplementary Fig. 10a, b supports reliable NH_3_ quantification. At −0.56 V, Fe-N_3_(PO_X_) reaches an NH_3_ yield rate of 21.96 g mg_Fe_^−1^ h^−1^, about four times that of Fe-N_4_ and better than most Fe-based catalysts as summarized later in Fig. 2g. At a more negative potential of −0.76 V, the yield rate reaches 43.39 g mg_Fe_^−1^ h^−1^. Figure 2c further shows that the Faradaic efficiency for NH_3_ is about 65.6% at −0.36 V and gradually approaches saturation as the potential shifts to more negative values, reaching 89.8% at − 0.56 V. Compared with Fe-N_4_, the FE increases more rapidly, confirming that Fe-N_3_(PO_X_) is highly selective toward NH_3_. Supplementary Fig. 11 shows that the Faradaic efficiencies for ${{{{\rm{NO}}}}}_{2}^{-}$ and H_2_ over both Fe-N_4_ and Fe-N_3_(PO_X_) are low, and the corresponding quantification by the Griess assay is validated by the calibration curves in Supplementary Fig. 12a, b, confirming the reliability of ${{{{\rm{NO}}}}}_{2}^{-}$ analysis. Furthermore, Fig. 2d presents the partial current densities for NH_3_ and ${{{{\rm{NO}}}}}_{2}^{-}$ over Fe-N_4_ and Fe-N_3_(PO_X_). It is clear that the current for Fe-N_3_(PO_X_) is dominated by NH_3_ production, and selective NH_3_ formation already begins at a relatively low potential of −0.46 V. To exclude differences in electrochemically active surface area (ECSA), Supplementary Fig. 13 reports the ECSA for each catalyst. Supplementary Fig. 13a, b shows CV curves at different scan rates, and Supplementary Fig. 13c shows similar C_*dl*_ values for Fe-N_4_ and Fe-N_3_(PO_X_), about 0.68 and 0.70 mF cm^−2^, indicating comparable active surface areas and similar numbers of single-atom sites. Supplementary Fig. 13d shows the current density at − 0.5 V after normalization, confirming that Fe-N_3_(PO_X_) delivers about four times the current of Fe-N_4_, which indicates that PO_X_ incorporation boosts the intrinsic NO_3_RR activity of Fe single-atom sites. Control experiments in pure 1.0 M KOH with and without KNO_3_ confirm that no NH_3_ is formed in the absence of KNO_3_, indicating that NO_X_ impurities do not interfere, as shown in Supplementary Fig. 14. Isotopic labeling in 1.0 M KOH with 0.1 M ${}^{15}{{{\rm{N}}}}{{{{\rm{O}}}}}_{3}^{-}$ and 0.1 M ${}^{14}{{{\rm{N}}}}{{{{\rm{O}}}}}_{3}^{-}$ further verified the nitrogen source. After electrolysis of ${}^{15}{{{\rm{N}}}}{{{{\rm{O}}}}}_{3}^{-}$, two ^1^H NMR signals assigned to ^15^NH_4_^+^ were observed. After electrolysis of ${}^{14}{{{\rm{N}}}}{{{{\rm{O}}}}}_{3}^{-}$, three signals assigned to ^14^NH_4_^+^ were observed, confirming that NH_3_ originates from nitrate reduction rather than contamination, as shown in Fig. 2e. These results confirm that NH_3_ originates from nitrate reduction rather than contamination. To clarify the effect of the PO_X_ ligand on the mechanism, time-dependent concentrations of ${{{{\rm{NO}}}}}_{3}^{-}$, ${{{{\rm{NO}}}}}_{2}^{-}$, and NH_3_ were measured at − 0.46 V vs RHE as shown in Supplementary Fig. 15. Calibration curves for ${{{{\rm{NO}}}}}_{3}^{-}$ is given in Supplementary Fig. 16, enabling accurate quantification. Fe-N_4_ shows slower NO_3_RR kinetics, with almost no change in ${{{{\rm{NO}}}}}_{3}^{-}$ concentration after 15 min of electrolysis. Fe-N_3_(PO_X_) shows faster kinetics, with about 10% ${{{{\rm{NO}}}}}_{3}^{-}$ consumption after 15 min and faster overall consumption curves, while neither catalyst produces significant ${{{{\rm{NO}}}}}_{2}^{-}$. These findings indicate that PO_X_ incorporation introduces spin state effects and electron delocalization that effectively and rapidly convert ${{{{\rm{NO}}}}}_{3}^{-}$ to NH_3_, thus enhancing NO_3_RR activity. Chronoamperometry of Fe-N_3_(PO_X_) at − 0.46 V versus RHE for nineteen periods of 0.25 h is shown in Supplementary Fig. 17. Cycling tests in Fig. 2f show that during continuous electrolysis, the Faradaic efficiency remains near 90% and the ammonia yield rate stays around 10 g mg_Fe_^−1^ h^−1^, demonstrating good long-term stability. A 24 h continuous electrolysis test at − 0.46 V to further evaluate the catalytic durability under prolonged operation (Supplementary Fig. 26). The current density remained stable during the 24 h electrolysis without obvious decay, demonstrating the good operational stability of the catalyst. After 24 h of continuous electrolysis, the catalyst still achieved a high Faradaic efficiency of 84.37% and an NH_3_ yield rate of 8.06 g mg_Fe_^−1^ h^−1^, which are close to the values obtained at − 0.46 V in the initial performance test. These results indicate that Fe-N_3_(PO_X_) can maintain stable NO_3_RR activity and selectivity under long-term operation. In addition, we performed XAS characterization before and after the stability test to examine whether the Fe sites maintain their atomic dispersion and local coordination environment after extended electrolysis. The XAS results before and after electrolysis indicate that the Fe species remain atomically dispersed. More importantly, the coordination number obtained from the post-reaction EXAFS fitting is 2.1, closely matching the value of 2.2 derived from the in situ EXAFS fitting at − 0.46 V. This result further supports that the catalyst retains a local coordination environment similar to the working-state active structure after prolonged operation (Supplementary Fig. 27 and Table 8). To further evaluate the practical feasibility of Fe-N_3_(PO_X_) under application-relevant NO_3_RR conditions, the catalyst was assembled in a flow-cell configuration using an FAA-130 alkaline anion-exchange membrane, and nitrate reduction measurements were performed. As shown in Supplementary Fig. 30a, the LSV curve shows that the current density exceeds 0.15 A cm^−2^ at a cell voltage of approximately 2.5 V, indicating effective nitrate reduction under flow-cell operation. Supplementary Fig. 30b further demonstrates the operational stability of Fe-N_3_(PO_X_) at 2.5 V, where the current response remains stable for approximately 20 h. The Faradaic efficiencies measured during the stability test are maintained at around 85%, confirming that stable NH_3_ selectivity is preserved under continuous flow-cell operation. Finally, Supplementary Fig. 30c compares the NH_3_ yield rate and Faradaic efficiency obtained at 2.5 V before and after the 20 h stability test. The comparable values further demonstrate that Fe-N_3_(PO_X_) can maintain stable NO_3_RR activity and selectivity in an alkaline AEM flow-cell system. Supplementary Fig. 18a compares this work with previously reported NO_3_RR SACs electrocatalysts in terms of the relationship between potential and NH_3_ yield rate, where the y-axis is plotted on a logarithmic scale, and the corresponding Faradaic efficiencies are summarized in Supplementary Fig. 18b^2,3,8,23,36–45^. Fe-N_3_(PO_X_) achieves gram-scale ammonia production, reaching 21.96 g mg_Fe_^−1^ h^−1^ with a Faradaic efficiency of 89.8% at the relatively low potential of − 0.56 V, and further increasing to 43.39 g mg_Fe_^−1^ h^−1^ at − 0.76 V. Notably, some of the better-performing Fe-based catalysts still require potentials of − 0.60 V or more negative to achieve gram-scale ammonia yield rates. To further enable a fairer comparison with representative NO_3_RR electrocatalysts with different catalyst architectures and metal loadings, the NH_3_ yield rate was also recalculated by normalizing it to the total catalyst mass and reported in g mg_cat_^−1^ h^−1^, as summarized in Supplementary Fig. 18c. This comparison based on total catalyst mass further supports the relatively competitive NO_3_RR performance of Fe-N_3_(PO_X_). A detailed comparison with previously reported NO_3_RR single-atom electrocatalysts is provided in Supplementary Table 11. Overall, Fe-N_3_(PO_X_) shows competitive performance among reported NO_3_RR single-atom electrocatalysts and maintains competitive activity when normalized to the total catalyst mass, supporting the effectiveness of PO_X_-modulated Fe single-atom sites for nitrate-to-ammonia conversion.

### In situ XAS tracks dynamic structural evolution in Fe-N_3_(PO_X_) SACs

**Fig. 3:**
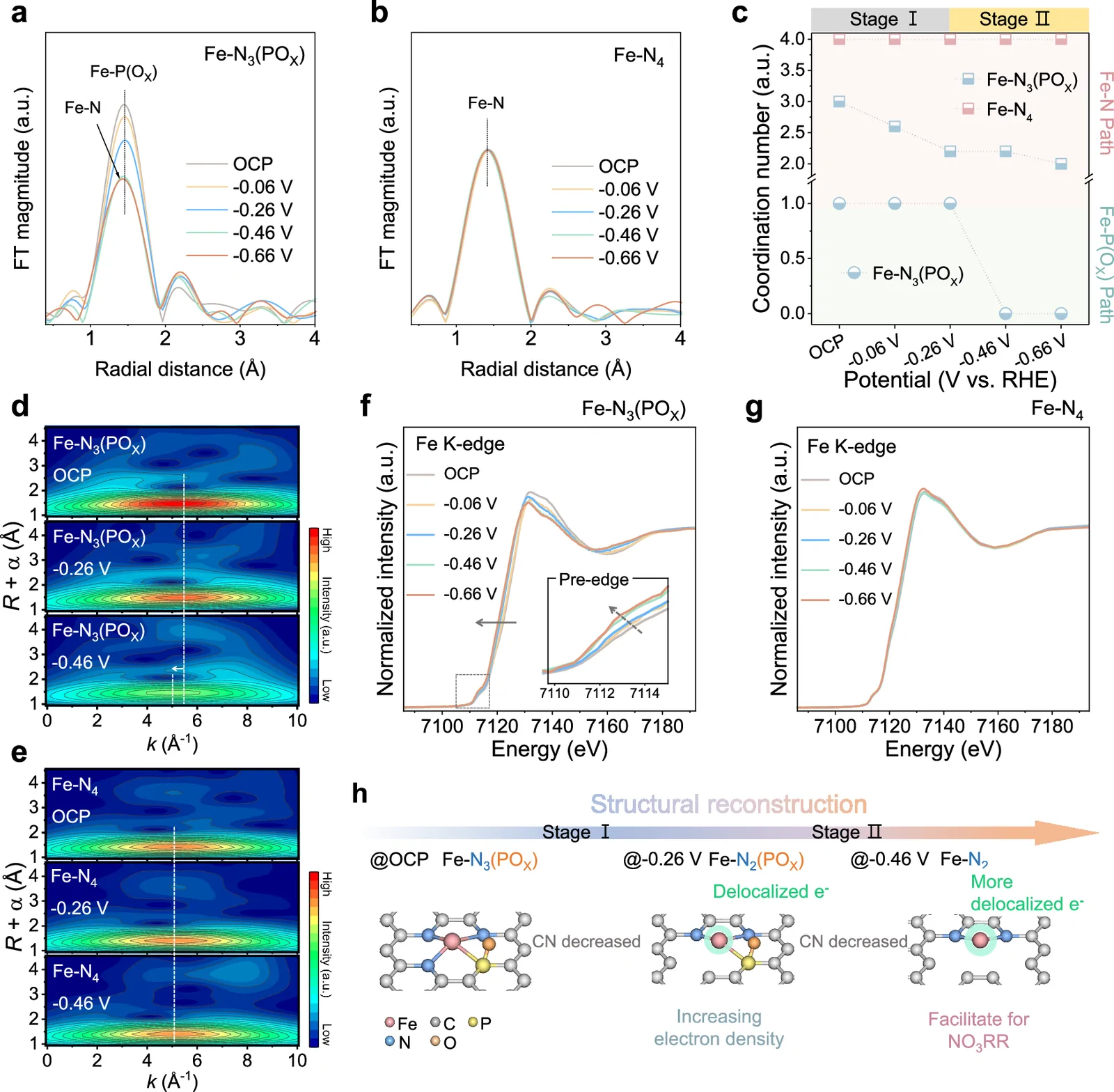
In situ XAS characterizations for the NO_3_RR. In situ Fe K-edge EXAFS spectra of (**a**) Fe-N_3_(PO_X_) and (**b**) Fe-N_4_ at different applied potentials. **c** Evolution of Fe-N and Fe-PO_X_ path coordination number change in Fe-N_3_(PO_X_) and Fe-N_4_. In situ WT-EXAFS contour plots of (**d**) Fe-N_3_(PO_X_) and (**e**) Fe-N_4_. In situ Fe K-edge XANES spectra of (**f**) Fe-N_3_(PO_X_) and (**g**) Fe-N_4_. **h** Proposed atomistic structures change of the active sites during NO_3_RR. All the potentials were not iR corrected. Measurements were performed in 1.0 M KOH + 0.1 M KNO_3_ at 25 °C using an in situ electrochemical cell. Source data are provided as a Source Data file.

In situ XAS has been widely used to probe dynamic catalyst changes under reaction conditions^46–49^. To elucidate how PO_X_ introduction sets the spin-active sites and the corresponding reaction mechanism, in situ XAS was performed. As shown in Fig. 3a, b, the corresponding 3D EXAFS maps reveal pronounced dynamic structural evolution during electrochemical operation. For Fe-N_3_(PO_X_) (Fig. 3a), the Fe-N/PO_X_ peak at ~ 1.5 Å gradually decreases in intensity during catalysis, indicating a reduction in coordination number. At potentials more negative than − 0.26 V the intensity becomes nearly constant, accompanied by a slight bond-length contraction. By contrast, Fe-N_4_ (Fig. 3b) shows essentially no change, indicating that this structure is relatively slow for the ammonia-producing cascade. Fourier-transformed EXAFS fits are provided in Supplementary Figs. 19, 20 and Supplementary Tables 4, 5. Figure 3c quantifies the Fe-center coordination environment. Notably, Fe-N_3_(PO_X_) shows two stages of change. From OCP to − 0.26 V, only the Fe-N coordination in Fe-N_3_(PO_X_) decreases. At more negative potentials, Fe-P(O_X_) bond cleavage becomes evident, suggesting that PO_X_ does not remain as a stable, independent primary active site throughout the reaction. On this basis, the enhanced catalytic performance is mainly attributed to the PO_X_-induced modulation and reconstruction of the Fe single-atom center, while PO_X_ may also contribute to the local coordination/electronic microenvironment. As seen in Supplementary Table 4, the Fe-N bond length in Fe-N_3_(PO_X_) increases slightly beyond − 0.26 V after PO_X_-ligand cleavage, consistent with the 3D EXAFS observations in Fig. 3a. This reconstruction is also corroborated by WT-EXAFS (Fig. 3d). The dashed line near k at around 5.5 Å^−1^ marks the region of maximal combined Fe-N/PO_X_ contribution. At − 0.26 V, the k-center of this ridge slightly shifts to higher k, whereas at − 0.46 V it shifts to lower k, consistent with Fe-PO_X_ bond scission. For Fe-N_4_, the Fe-N signal remains constant (Fig. 3e), matching the trend in Fig. 3c. Altogether, the coordination number in Fe-N_3_(PO_X_) decreases stepwise. Fe-N breaks first, followed by Fe-PO_X_ cleavage beyond − 0.26 V, ultimately forming an intermediate Fe-N_2_ state with a slightly elongated Fe-N bond. Thus, the asymmetric spin chemistry of Fe-N_3_(PO_X_) activates a dynamic reconstruction switch during NO_3_RR, and the P-induced asymmetry-driven reconstruction is key to the improved Faradaic efficiency.

In situ Fe K-edge XANES (Fig. 3f, g) links electronic with atomic-structure changes. For Fe-N_3_(PO_X_) (Fig. 3f), from OCP to − 0.26 V, the Fe electronic structure shows only a slight reduction. Upon further decrease to − 0.46 V, the edge position shifts markedly to lower energy, indicating concurrent adjustments of coordination and electronic structure. Increased electron density at Fe sites promotes delocalization and thereby weakens ${{{{\rm{NO}}}}}_{3}^{-}$ adsorption, accelerating NO_3_RR. In the Fig. 3f inset, the pre-edge intensity increases from − 0.26 to − 0.46 V and then plateaus, in good agreement with the coordination changes above. Fe-N_4_ catalyst in Fig. 3g shows a negligible change. Supplementary Fig. 21 further highlights these trends. In Fe-N_3_(PO_X_), the oxidation state drops slightly from OCP to − 0.26 V and then decreases sharply beyond − 0.26 V, consistent with Fe-PO_X_ bond cleavage and aligned with the electrochemical FE maximum near − 0.46 V. In contrast, symmetric Fe-N_4_ shows no obvious electronic or atomic structure changes. These results indicate that triggering dynamic reconstruction accompanied by valence-state changes is crucial for efficient ammonia production. Figure 3h summarizes the atomic and electronic evolution of Fe-N_3_(PO_X_). Compared with inert symmetric Fe-N_4_, the asymmetric spin chemistry drives a two-step electronic-structure change that yields higher NH_3_ yield and Faradaic efficiency. After PO_X_ substitution, the resulting asymmetry creates spin states in Fe sites with partially filled *e*_*g*_ orbitals that favor NO_3_RR and act as a switch for stepwise evolution. Specifically, Fe-N breaks first, then Fe-PO_X_ cleavage occurs beyond −0.26 V, accompanied by gradually increasing electron delocalization. The structure ultimately evolves to the active Fe-N_2_ state, delivering higher NH_3_ yield and Faradaic efficiency.

### In situ ATR-FTIR and XES reveal intermediates and spin state coupling

**Fig. 4:**
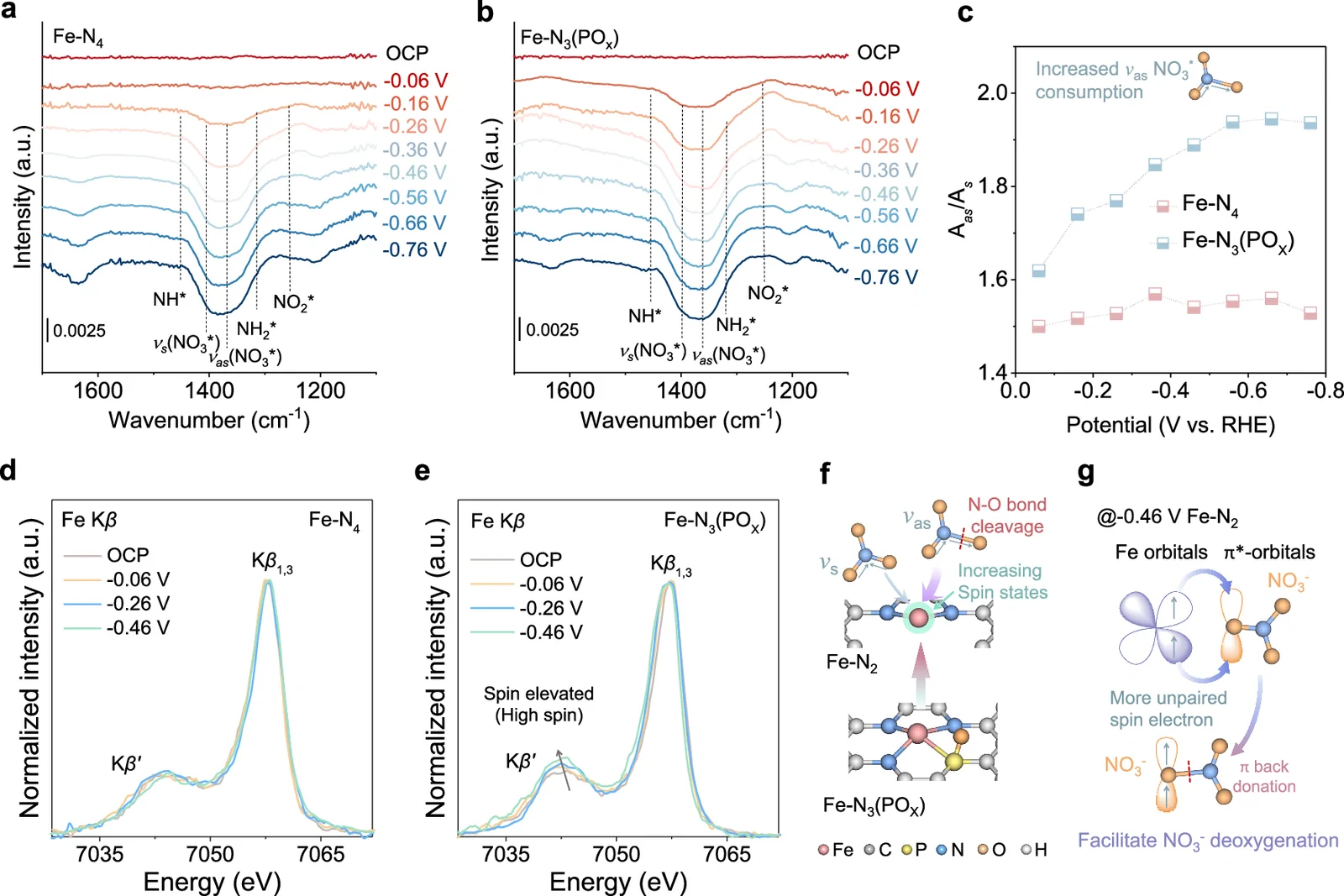
In situ ATR-FTIR and XES mechanistic study of NO_3_RR. In situ ATR-FTIR spectra of (**a**) Fe-N_4_ and (**b**) Fe-N_3_(PO_X_) during potential scan from OCP to − 0.76 V. **c** Evolution of the intensity ratio between symmetric and asymmetric NO_3_* peak ratio. In situ XES spectra of (**d**) Fe-N_4_ and (**e**) Fe-N_3_(PO_X_). **f** Schematic of spin state evolution in Fe-N_3_(PO_X_), enhancing asymmetric nitrate activation and consumption. **g** Proposed ligand-interaction reaction mechanism for NO_3_RR. All the potentials were not iR corrected. Measurements were performed in 1.0 M KOH + 0.1 M KNO_3_ at 25 °C using the corresponding in situ electrochemical cells. Source data are provided as a Source Data file.

To clarify how PO_X_-induced dynamics enhance nitrate reduction, in situ ATR-FTIR and XES were used to correlate surface speciation with the ammonia-formation pathway. In situ ATR-FTIR first probed reaction intermediates (Fig. 4a, b). The waterfall plots show five key bands at 1230, 1290, 1350, 1390, and 1450 cm^−1^ ^50–52^ Negative signals at 1350 and 1390 cm^−1^ are assigned to the N-O asymmetric and symmetric stretching modes *ν*_as_(N-O) and *ν*_s_(N-O), reflecting consumption of the corresponding nitrate-ligand vibrations. Nitrate has a planar D_3h_-symmetric structure with three equivalent N-O bonds under ideal conditions. However, when one N-O group preferentially interacts with the catalyst surface or nearby hydrogen-bonding environment, local symmetry breaking may occur, accompanied by transient N-O bond elongation and charge redistribution within the nitrate ion^53,54^. Such an asymmetric adsorption configuration may promote local N-O bond activation and facilitate subsequent hydrogenation/deoxygenation-related steps, thereby improving NO_3_RR kinetics. Positive peaks indicate formation of species, with 1230 cm^−1^ assigned to NO_2_^*^, 1290 cm^−1^ to NH_2_^*^, and 1450 cm^−1^ to NH^*^. In Fig. 4a a clear nitrate-consumption band is observed, and the symmetric nitrate feature at 1390 cm^−1^ dominates. As the potential is stepped negatively in 0.1 V increments, this symmetric vibration is continually consumed while the asymmetric band remains weaker than the symmetric band. Figure 4b further shows that the 1350 cm^−1^ shoulder attributable to asymmetric nitrate grows steadily at more negative potentials, indicating increased consumption via *ν*_as_(N-O). Compared with Fe-N_4_, stronger nitrate consumption is already evident under negative bias, and the asymmetric band becomes dominant. This means PO_X_ initially tunes ${{{{\rm{NO}}}}}_{3}^{-}$ adsorption and then acts as a switch that drives further structural evolution to enhance ammonia formation. Features assigned to NH^*^ and NH_2_^*^ intermediates are also much stronger than on Fe-N_4_ in Fig. 4b, reinforcing this interpretation. Overall, with structural evolution on Fe-N_3_(PO_X_), the O atom bearing a partial negative charge in the asymmetric mode is more readily hydrogenated by a nearby adsorbed H, which accelerates subsequent ammonia-forming steps^53,54^. Figure 4c quantifies these trends using the *ν*_as_/*ν*_s_ ratio. The corresponding fitting plots and detailed parameters are provided in Supplementary Fig. 22 and Table 6. Fe-N_3_(PO_X_) catalyst shows a higher asymmetric-consumption fraction than Fe-N_4_, and the ratio increases with potential magnitude and saturates near − 0.66 V. This agrees with in situ XAS evidence for coordinated adjustments of coordination and electronic structure and for increased electron delocalization. The result is enhanced asymmetric nitrate consumption and an FE maximum consistent with the electrochemical data. In situ XES directly probes interactions between spin state and nitrate. In Fig. 4d for Fe-N_4_, the K*β*′ feature near 7045 eV is essentially unchanged, and the K*β*_1,3_ near 7060 eV does not shift, indicating minimal change in electronic structure. This implies that Fe lower spin state donates electrons to ${{{{\rm{NO}}}}}_{3}^{-}$ weakly and struggles to promote asymmetric nitrate intermediates, consistent with the inert behavior above. By contrast, Fig. 4e shows that in Fe-N_3_(PO_X_), the K*β*_1,3_ shifts to higher energy while K*β*′ intensity increases at more negative potentials, indicating a lower Fe valence together with a rising spin state. To further quantify this potential-dependent spin-state evolution, the in situ Fe K*β* XES spectra of Fe-N_3_(PO_X_) were analyzed by Gaussian fitting. The K*β* emission profiles were deconvoluted into the K*β*_1,3_ main emission line and the K*β*′ satellite feature, and the relative K*β*′ peak areas were extracted for spin state evaluation (Supplementary Fig. 28 and Supplementary Table 9). The estimated average spin state of Fe-N_3_(PO_X_) gradually increases from 1.96 at OCP to 2.23 at − 0.46 V during NO_3_RR (Supplementary Fig. 29), confirming a potential-dependent enhancement of the higher-spin character of the Fe center. This dynamically enhanced higher-spin character may facilitate back-donation from Fe 3 *d* into the ${{{{\rm{NO}}}}}_{3}^{-}$ π* orbitals, weakens the N-O σ bond, and drives the subsequent nitrate-reduction sequence. Supplementary Fig. 23 summarizes how structural evolution impacts spin. In PO_X_-induced asymmetric Fe-N_3_(PO_X_), the total coordination number gradually decreases while the total spin increases. This trend closely tracks the *ν*_as_/*ν*_s_ evolution in Fig. 4c, indicating that the initially spin state of Fe-N_3_(PO_X_) switches the structure toward Fe-N_2_ while electron density and delocalization increase. Higher spin state donates electrons to ${{{{\rm{NO}}}}}_{3}^{-}$ more effectively and increases the asymmetric fraction (Fig. 4f). A simplified schematic of ${{{{\rm{NO}}}}}_{3}^{-}$ bonding to Fe single-atom sites is shown in Fig. 4g. Accordingly, Fe-N_3_(PO_X_) with spin-polarized sites leverages spin alignment to donate electrons to ${{{{\rm{NO}}}}}_{3}^{-}$ more effectively than Fe-N_4_, resulting in a higher NO_3_RR rate. Taken together, these multimodal spectroscopies tightly link mechanism with electronic structure. PO_X_-induced asymmetry increases the spin state, first mitigating the overly strong ${{{{\rm{NO}}}}}_{3}^{-}$ adsorption characteristic of Fe-N_4_, then acting as a switch that enables adaptive structural evolution toward Fe-N_2_. Based on the in situ XAS results, this Fe-N_2_ intermediate is difficult to obtain from symmetric Fe-N_4_, whereas on active Fe-N_3_(PO_X_), it is reached as the spin increases. The higher spin state character makes the interaction between Fe sites in Fe-N_3_(PO_X_) and ${{{{\rm{NO}}}}}_{3}^{-}$ more compatible, promotes population of NO_3_ π* orbitals, and enhances overall catalytic activity.

### Theoretical simulations of the NO_3_RR mechanism

**Fig. 5:**
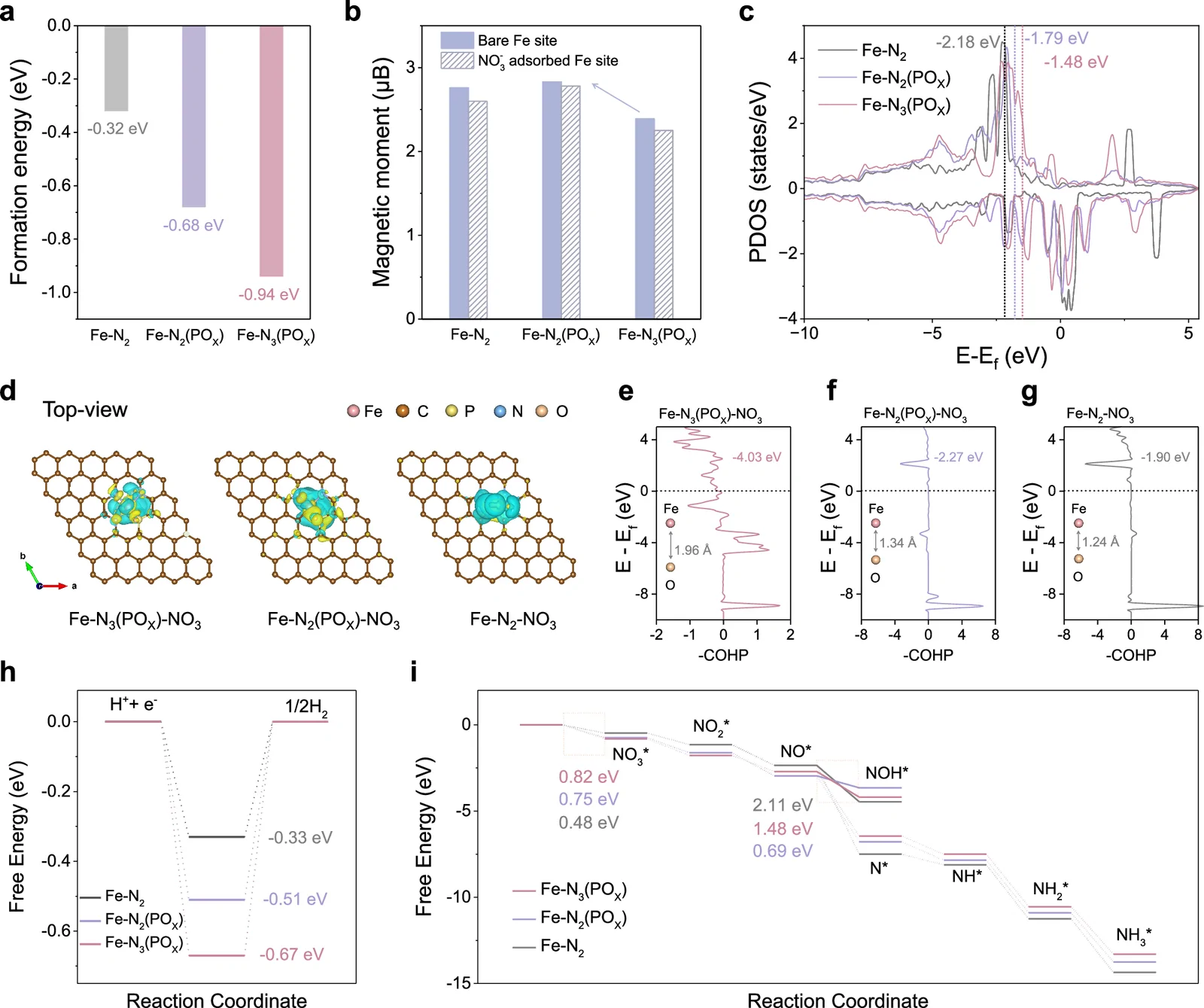
Theoretical investigation of the reconstructed active configuration for NO_3_RR. **a** Formation energy of Fe-N_2_, Fe-N_2_(PO_X_) and Fe-N_3_(PO_X_) models. **b** Magnetic moments of the three models with before and after ${{{{\rm{NO}}}}}_{3}^{-}$ adsorption. **c** Fe 3 *d* projected density of states and *d*-band center. **d** Charge density difference of three ${{{{\rm{NO}}}}}_{3}^{-}$-adsorbed models. The yellow and cyan is isosurfaces represent the charge accumulation and charge depletion regions, respectively. COHP analyses of Fe-O interactions in (**e**) Fe-N_3_(PO_X_), (**f**) Fe-N_2_(PO_X_) and (**g**) Fe-N_2_. **h** Gibbs free energies of H adsorption of Fe-N_2_, Fe-N_2_(PO_X_) and Fe-N_3_(PO_X_). **i** Gibbs free-energy diagrams for the NO_3_RR pathway over the three models. Source data are provided as a Source Data file.

To elucidate how PO_X_-assisted coordination reconstruction regulates the NO_3_RR mechanism, DFT calculations and MD were performed using Fe-N_3_(PO_X_), Fe-N_2_(PO_X_) and Fe-N_2_ as representative working-state configurations, with their optimized atomic structures provided in Supplementary Fig. 31. These models describe the progressive coordination evolution of the Fe center observed by in situ XAS rather than three independent static active sites. The calculated formation energies of Fe-N_2_, Fe-N_2_(PO_X_) and Fe-N_3_(PO_X_) are − 0.32, − 0.68 and − 0.94 eV, respectively (Fig. 5a), demonstrating that PO_X_ stabilizes the asymmetric Fe coordination environment and provides a thermodynamically accessible platform for subsequent potential-driven reconstruction. The Fe local magnetic moment increases from 2.39 μB for Fe-N_3_(PO_X_) to 2.83 μB for Fe-N_2_(PO_X_) and remains high after ${{{{\rm{NO}}}}}_{3}^{-}$ adsorption (2.78 μB), identifying Fe-N_2_(PO_X_) as a highly spin-polarized state (Fig. 5b). This trend agrees well with the increased higher-spin character observed by in situ Fe K*β* XES. Electronic-structure calculations further reveal coordination-dependent Fe-adsorbate interactions. The d-band centers shift from − 1.48 eV for Fe-N_3_(PO_X_) to − 1.79 eV for Fe-N_2_(PO_X_) and − 2.18 eV for Fe-N_2_ (Fig. 5c). Charge density difference and COHP analyses show the strongest Fe-NO_3_ orbital coupling on Fe-N_3_(PO_X_) (-ICOHP = 4.03 eV), followed by Fe-N_2_(PO_X_) (2.27 eV) and Fe-N_2_ (1.90 eV) (Fig. 5d–g). These results indicate that Fe-N_3_(PO_X_) favors initial nitrate adsorption and activation, whereas partial reconstruction to Fe-N_2_(PO_X_) moderates the Fe-NO_3_ interaction while retaining effective electronic coupling. The calculated H adsorption free energies are − 0.67, − 0.51 and − 0.33 eV for Fe-N_3_(PO_X_), Fe-N_2_(PO_X_) and Fe-N_2_, respectively (Fig. 5h), with the corresponding H adsorption configurations provided in Supplementary Fig. 32 The intermediate H-binding strength of Fe-N_2_(PO_X_), together with its enhanced Fe spin polarization and moderate Fe-NO_3_ coupling, provides a balanced local environment for hydrogenation of nitrate-derived intermediates. Further, the process from NO* to NOH* hydrogenation step requires 2.11 eV on Fe-N_3_(PO_X_) and 1.48 eV on Fe-N_2_ but only 0.69 eV on Fe-N_2_(PO_X_) (Fig. 5i). The optimized adsorption configurations of key NO_3_RR intermediates on the three models are provided in Supplementary Figs. 33–35 and Table 10. Thus, potential-driven reconstruction from Fe-N_3_(PO_X_) to Fe-N_2_(PO_X_) substantially facilitates the critical NO hydrogenation step, whereas the further reconstructed Fe-N_2_ configuration favors downstream conversion of N-containing intermediates toward NH_3_. Therefore, the calculations support a dynamic relay mechanism in which Fe-N_3_(PO_X_) acts primarily as a nitrate-activation state, Fe-N_2_(PO_X_) serves as a spin-polarized hydrogenation state, and Fe-N_2_ facilitates downstream hydrogenation and NH_3_ formation. PO_X_ therefore promotes NO_3_RR not through a single static active site, but by enabling sequential coordination states with complementary catalytic functions, providing a theoretical basis for the experimentally observed coupling among coordination reconstruction, spin-state enhancement, asymmetric nitrate activation, and accelerated ammonia formation.

## Discussion

This study proposes a Fe-N_3_(PO_X_) single-atom configuration with highly adaptable electronic and atomic dynamics that can accommodate complex multielectron transfer processes. In the Fe-N_3_(PO_X_) SACs, PO_X_ both moderates the initially overly strong ${{{{\rm{NO}}}}}_{3}^{-}$ adsorption and acts as a switch that breaks the symmetric Fe-N_4_ coordination structure, thereby triggering adaptive coordination dynamics and spin state changes. Multiple in situ characterizations confirm that the initially spin state in Fe-N_3_(PO_X_) sites gradually evolves under applied bias into a highly active Fe-N_2_ structure with a higher-spin state, which promotes the preferential consumption of asymmetric ${{{{\rm{NO}}}}}_{3}^{-}$ species. The resulting coupled electronic-atomic self-adaptation at the Fe center generates higher spin electrons that drive efficient selectivity and fast reaction kinetics in NO_3_RR. This work achieves precise control of active sites at the single-atom level and direct tracking of the true active centers and their reaction dynamics, providing a valuable blueprint that establishes clear correlations among coordination environment, spin state, and catalytic performance, and offering concrete, practical guidelines for the rational design of efficient NO_3_RR electrocatalysts.

## Methods

### Chemicals and reagents

Iron (III) chloride anhydrous (Alfa Aesar, 98%, FeCl_3_). D-(+)-Glucose (Sigma-Aldrich, ≧ 99.5%). Sodium Chloride (Seedchem, 99%, NaCl). Potassium nitrate (Thermo Scientific, 99%, KNO_3_). Potassium hydroxide (Sigma-Aldrich, ACS reagent, ≥ 85%, KOH). D2020CS Nafion^®^ dispersion (Chemours^TM^, 20%). Salicylic acid (Thermo Scientific, 99%). Trisodium citrate dihydrate (Showa, 99%). Sodium hydroxide (Showa, 97%, NaOH). Sodium hypochlorite solution (Showa, 10 ~ 12%, NaClO). Sodium pentacyanonitrosylferrate (III) dihydrate (Alfa Aesar, 98%). N-(1-Naphthyl) ethylenediamine, dihydrochloride (Thermo Scientific, ACS reagent, 98%). Sulfanilamide (Alfa Aesar, 98%). Phosphoric acid (Sigma-Aldrich, 85 wt.% in H_2_O, 99.99% trace metals basis, H_3_PO_4_). Ferric phosphate (ANGENE, 99%, FePO_4_). Sulfamic acid (ACS reagent, 99.3%). Hydrochloric acid (SHOWA, 35%, HCl). Platinum foil (99%, Pt). Carbon cloth (CeTech Co., Ltd). Hg/HgO reference electrode (RE-61AP, BAS Inc., Japan).

### Preparation of Fe single-atom catalyst

4.3874 g NaCl, 0.426 g D-glucose, and 100 mg iron (III) nitrate nonahydrate Fe(NO_3_)_3_·9H_2_O, equivalent to 500 ppm Fe, were dissolved in 100 mL deionized water (DI water) and stirred for 30 min until a clear solution was obtained. The solution was pre-frozen and freeze-dried at − 50 °C and < 0.1 mbar for 24 h to obtain a dry composite.

The resulting powder was lightly ground, loaded into an alumina boat and placed in a quartz tube furnace. The furnace was purged with NH_3_ for 30 min (100 mL min^−1^), heated to 500 °C for 4 h at a heating rate 5 °C min^−1^ under NH_3_ and then cooled to room temperature (25 °C).

The resulting black solid was washed three times with 2 L DI water each time to remove NaCl, collected by vacuum filtration and dried at room temperature overnight to obtain the Fe single-atom catalyst.

### Preparation of Fe-N_3_(PO_X_) single-atom catalyst

15 mg Fe SACs were thoroughly mixed with 10 mg NH_2_PO_2_ to obtain a uniform powder. The mixture was pressed into uniform pellets using a die press. The pellets were placed in an alumina boat and loaded into a quartz tube furnace. After evacuation to 10^−2^ torr, the sample was heated at 300 °C for 4 h. It was then cooled to room temperature under vacuum. The resulting product was collected as the Fe-N_3_(PO_X_) single-atom catalyst.

### Characterization

The morphology of the catalysts was characterized using SEM (JEOL, JSM-6700F) and TEM (JEOL, ARM-200FTH). The HR-PXRD was performed at the TPS-19A of National Synchrotron Radiation Research Center (NSRRC), Taiwan. The high-resolution XPS were measured at the TLS-24A of NSRRC, Taiwan. The XPS measurements were performed under high vacuum conditions (< 10^−6^ bar). The binding energies of collected spectra were calibrated to Au 4 *f*_7/2_ of 84 eV for comparison. The XAS measurements were performed in fluorescence mode at the TPS-44A of NSRRC, Taiwan. The acquired XANES and EXAFS data were processed with ATHENA, and EXAFS fitting was carried out using the ARTEMIS module of the Demeter software package^55^. Further details are provided in the Supplementary Information.

### Electrochemical measurements

All the electrochemical performance were carried out in a homemade two-compartment H-cell separated with a Nafion^®^117 membrane with a thickness of 183 µm at room temperature. Each compartment contained 20 mL of electrolyte, with 1.0 M KOH in the anodic compartment and 1.0 M KOH and 0.1 M KNO_3_ in the cathodic compartment. The Nafion^®^117 membrane was pretreated in 3% H_2_O_2_ at 80 °C for 1 h and thoroughly rinsed with DI water. The membrane was subsequently treated in 0.5 M H_2_SO_4_ at 80 °C for 1 h, rinsed repeatedly with DI water to remove residual acid and stored in DI water before use. The measurements were performed in a conventional three-electrode configuration connected with a CHI 6278e electrochemical workstation (CH Instruments, Inc., USA) with platinum foil as a counter electrode and Hg/HgO as a reference electrode. Working electrodes were prepared by drop-casting the catalyst ink onto carbon cloth. Specifically, a 20 wt% Nafion stock solution was first diluted with absolute ethanol to obtain a 5 wt% Nafion solution. The dispersion medium was then prepared by mixing 1.0 mL of 5 wt% Nafion solution with 4.5 mL of ethanol and 4.5 mL of DI water. Subsequently, 0.05 mg of catalyst powder was dispersed in 20 μL of the dispersion medium and sonicated to form a homogeneous catalyst ink. Finally, the entire catalyst ink was drop-cast onto carbon cloth (1 cm^2^), resulting in a catalyst loading of 0.05 mg cm^−2^. The potentials reported in this work were all against Reversible Hydrogen Electrode (RHE, E_RHE_) corrected with Equation (1): ${{{{\rm{E}}}}}_{{{{\rm{RHE}}}}}={{{{\rm{E}}}}}_{{{{\rm{Hg}}}}/{{{\rm{HgO}}}}}+0.118+0.059{{{\rm{pH}}}}$, where 0.118 V is the Hg/HgO reference potential.

LSV measurements were conducted in 1.0 M KOH, with or without 0.1 M KNO_3_, over a potential range of 0 to − 0.8 V vs. RHE, with 90% iR correction at a scan rate of 5 mV s^−1^. The solution resistance of the electrochemical cell was measured using the built-in iR measurement function of a CHI electrochemical workstation under OCP conditions. A stock electrolyte containing 2.0 M KOH and 0.2 M KNO_3_ in DI water was stored in a tightly sealed container at room temperature and used within 5 days of preparation. Prior to each measurement, the stock electrolyte was thoroughly mixed with an equal volume of DI water to obtain 1.0 M KOH containing 0.1 M KNO_3_. The pH values of 1.0 M KOH and 1.0 M KOH containing 0.1 M KNO_3_ are 13.83 ± 0.10 and 13.84 ± 0.10, respectively. Double-layer capacitance (C_dl_) was determined using cyclic voltammograms within the non-Faraday current range (open circuit potential ± 50 mV) under various scan rates of 20–100 mV s^−1^ at 20 mV steps. C_dl_ was calculated using the formula of C_dl _= *i*_*v*_/(dE/dt) (where *i*_*v*_ is half of the difference between the anodic and cathodic currents at the open-circuit potential; dE/dt represents the scan rate). Current density based on electrochemical surface area (*j*_*ECSA*_) was normalized using the formula of *I* × C_s_/C_dl_ (where *I* is the current based on geometric area; C_s_ represents specific capacitances^56^). Chronoamperometric measurements were performed in 1.0 M KOH with 0.1 M KNO_3_ under various applied potentials for 30 min without iR correction. Cell cycling experiments were performed under ambient laboratory conditions at room temperature. All the electrochemical process were performed under Ar purged unless otherwise mentioned. All error bars throughout the manuscript represent the standard deviation from three independent measurements. The NH_3_ yield rate and Faradaic efficiency values reported are based on single measurements. The practical NO_3_RR performance of Fe-N_3_(PO_X_) was evaluated in a membrane-electrode-assembly flow-cell reactor with an effective electrode area of 1 cm^2^, using a 1.5 × 1.5 cm^2^ FAA-130 alkaline anion-exchange membrane with a thickness of 130 µm. Before the measurements, the FAA-130 membrane was pretreated in 1.0 M KOH for at least 24 h to convert it into the OH^-^ form. The catalyst ink contained 5 mg mL^−1^ Fe-N_3_(PO_X_) in an IPA/DI water mixed solvent (v/v = 3:1) with 0.4 vol% of 20 wt% Nafion solution. Then, 100 μL of the catalyst ink was loaded onto a 1 cm^2^ carbon cloth substrate to achieve a catalyst loading of 0.5 mg cm^−2^. A 1 cm^2^ Ni foam was used as the anode for the oxygen evolution reaction. During the flow-cell NO_3_RR measurements, 1.0 M KOH containing 0.1 M KNO_3_ was used as the catholyte, while 1.0 M KOH was used as the anolyte. The catholyte and anolyte were each stored in a 1 L reservoir and continuously circulated through the corresponding flow channels at a flow rate of 50 mL min^−1^.

### Quantitative analysis of NH_3_

The indophenol method was used to quantify the formation of NH_3_ after each electrolysis. Specifically, the catholyte after the reaction was collected and diluted to a detection range within a calibration curve. Subsequently, 2 mL of diluted electrolyte was mixed with 2 mL of reagent containing salicylic acid (5 wt%) and sodium citrate (5 wt%). Finally, a 1 mL sodium hypochlorite (0.05 M) followed by the addition of 0.2 mL of sodium nitroprusside solution (1 wt%) was further added and thoroughly mixed. After incubating the mixture at room temperature for 2 h, the UV-vis measurements were carried out using a HITACHI U-3900 spectrophotometer over the wavelength range of 800–500 nm, with the absorbance at 650 nm extracted for NH_3_ quantification. NH_3_ Faradaic efficiency and NH_3_ yield rate are calculated with Equation (2): NH_3_ Faradaic Efficiency (%) = (N_NH3_ × F × 8)/Q and Equation (3): NH_3_ Yield rate (mg_NH3_ mg_Fe_^−1^ h^−1^) = (N_NH3_ × 17.03 × 10^−3^)/(m_cat_ × t), respectively. Where N_NH3_ is mole of generated NH_3_; F is Faraday constant; 8 is the electron transfer number of ${{{{\rm{NO}}}}}_{3}^{-}$ convert to NH_3_; Q is the total charge passage during the electrolysis; 17.03 is molecule weight of NH_3_; m_cat_ is the loading amount of catalysts; and t is the electrolysis duration.

### Quantitative analysis of ${{{{\rm{NO}}}}}_{2}^{-}$

The Griess test was used to quantify the formation of ${{{{\rm{NO}}}}}_{2}^{-}$ after each electrolysis. In detail, Griess reagent was prepared with 0.2 mL of H_3_PO_4_ and 1 mL DI water solvent dissolved with 80 mg N-(1-naphthyl) ethylenediamine dihydrochloride and 4 mg sulfanilamide. Additionally, the catholyte after the reaction was collected and diluted to a detection range within a calibration curve. Subsequently, 2 mL of 1 M HCl was added to neutralize the pH, followed by the addition of 0.08 mL of Griess reagent. After incubating the mixture at room temperature for 15 min, the UV-vis measurements were carried out over the wavelength range of 650–400 nm, with the absorbance at 540 nm extracted for ${{{{\rm{NO}}}}}_{2}^{-}$ quantification. ${{{{\rm{NO}}}}}_{2}^{-}$ Faradaic Efficiency (%) is calculated according to Equation (4): ${{{{\rm{NO}}}}}_{2}^{-}$ Faradaic Efficiency (%) = (N_NO2_^- ^× F × 2)/Q. Where N_NO2-_ is mole of generated ${{{{\rm{NO}}}}}_{2}^{-}$; F is Faraday constant; 2 is the electron transfer number of ${{{{\rm{NO}}}}}_{3}^{-}$ converted to ${{{{\rm{NO}}}}}_{2}^{-}$; Q is the total charge passage during the electrolysis.

### Quantitative analysis of ${{{{\rm{NO}}}}}_{3}^{-}$

The conversion of ${{{{\rm{NO}}}}}_{3}^{-}$ was detected under − 0.3 V vs. RHE for different durations. To facilitate the evaluation of conversion, the working electrode would be enlarged to 4 cm^2^ while maintaining the identical catalyst loading (0.05 mg cm^−2^). For the ${{{{\rm{NO}}}}}_{3}^{-}$ detection, the catholyte after the reaction was collected and diluted by a factor of 160. Subsequently, the diluted catholyte was initially neutralized by adding 3 mL of 1 M HCl, followed by adding 0.1 mL of sulfamic acid (0.8%). After incubating the mixture at room temperature for 15 min, the UV-vis measurements were carried out over the wavelength range of 300–200 nm, with the absorbance calculated using the formula of A_220_-2A_275_ (where A_220_ and A_275_ is the absorbance at 220 nm and 275 nm, respectively). ${{{{\rm{NO}}}}}_{3}^{-}$ concentration (mM) is calculated according to Equation (5): ${{{{\rm{NO}}}}}_{3}^{-}$ concentration $({{{\rm{mM}}}})={{{{\rm{N}}}}}_{{{{{\rm{NO}}}}}_{{3}^{-}}}/{{{\rm{V}}}}$. Where ${{{{\rm{N}}}}}_{{{{{\rm{NO}}}}}_{{3}^{-}}}$ is mole of generated ${{{{\rm{NO}}}}}_{3}^{-}$; V is the volume of the catholyte.

### Quantitative analysis of H_2_

The amount of generated H_2_ was quantified by gas chromatography (GC). The Faradaic efficiency for H_2_ production was calculated according to Equation (6): H_2_ Faradaic Efficiency (%) = (N_H2 _× F × 2)/Q., where F is the Faraday constant, nH_2_ is the amount of H_2_ quantified by GC, and Q is the total charge passed during electrolysis.

### ^15^N isotope labeling experiments

To qualitatively confirm the nitrogen source of the produced NH_3_, the electrolyte consisting of 1.0 M KOH and 0.1 M KNO_3_ was replaced with 1.0 M KOH and 0.1 M K^15^NO_3_. Both electrolytes were subjected to electrolysis at −0.3 V vs. RHE for 0.5 hours. After electrolysis, 580 μL of the electrolyte was mixed with 50 μL of 6 M H_2_SO_4_ and 25 μL of dimethyl sulfoxide-d_6_ (DMSO-d_6_) to obtain the ^1^H NMR spectrum. The ^15^N isotope labeling experiments were conducted using a VARIAN VNMRS-600 NMR spectrometer.

### In situ XAS measurements

The Fe K-edge XAS was measured at the TPS-44A of NSRRC, Taiwan. In situ XAS measurements were performed in the self-assembled Teflon cell (Supplementary Fig. 36a). The X-ray beam was transmitted through the Kapton tape and electrolyte and reached the detector for XAS collection in the fluorescence mode. Platinum was chosen as the counter electrode, while an Hg/HgO electrode served as the reference electrode, with electrolyte of 1.0 M KOH + 0.1 M KNO_3_. For the working electrode preparation, 10 mg of catalyst powder were dissolved in ethanol containing 50 μL of a 5% Nafion solution and sonicated for 20 min to form a homogeneous ink. Subsequently, the mixed solution was uniformly drop-coated onto a 1 × 1 cm^2^ carbon cloth and allowed to dry to obtain the working electrode.

### In situ XES measurements

The Fe XES measurements was conducted at the BL-12XU of SPring-8, Japan. In situ XES measurements were performed in the self-assembled Teflon cell, as that used for the XAS measurements. The X-ray beam was transmitted through the Kapton tape and electrolyte. In a typical three-electrode setup, the working electrode was a piece of 1 × 1 cm^2^ carbon cloth uniformly drop-cast with catalyst ink containing 20 mg of catalyst, with the Hg/HgO electrode and platinum acting as the reference and counter electrodes, respectively, in a solution of 1.0 M KOH + 0.1 M KNO_3_. The fluorescence was separated by the analyzer crystal Si (444) and collected by a silicon drift detector (XR-100CR Si-PIN X-ray detector).

### In situ ATR-FTIR measurements

ATR-FTIR experiments were carried out at the TLS-14A1 of NSRRC, Taiwan. The reflecting surface of a 60° silicon prism (Veemax, 2 cm in diameter) was polished with alumina paste to expose a fresh silicon layer. Then, a solution of 1 mg of Fe-N_4_ and Fe-N_3_(PO_X_) catalysts mixed with 100 μL of Nafion ink was dropped onto the fresh surface of the silicon prism after ultrasonic mixing. Subsequently, three small pieces of conductive copper tape were adhered around the circumference of the fresh silicon surface, and finally, an in situ cell was assembled (Supplementary Fig. 36b). The three-electrode cell system used in the experiment included a working electrode with Fe-N_4_ and Fe-N_3_(PO_X_) catalysts, a reference electrode (Hg/HgO), and a platinum electrode. This cell was integrated into a Fourier transform infrared spectrometer (Thermo Scientific Nicolet 6700 FT-IR), equipped with a mercury-cadmium telluride (MCT) detector and a specular reflectance accessory set at a 65° angle (Veemax III, Pike Technology). A baseline spectrum was recorded at OCP using a CHI Instrument, followed by a series of applied voltage steps (each overpotential maintained for 3 min) within the potential range of 0.06 V to − 0.76 V vs. RHE, collecting the corresponding infrared spectra.

#### Density functional theory calculations

Density functional theory (DFT) calculations were performed to clarify the electronic origin of the PO_X_-induced coordination reconstruction and its influence on the NO_3_RR. All spin-polarized calculations were carried out using the Vienna Ab initio Simulation Package (VASP) based on the MeadA platform. The projector augmented wave (PAW) method was employed to describe the ion-electron interactions, and the exchange-correlation energy was treated using the Perdew-Burke-Ernzerhof (PBE) functional within the generalized gradient approximation (GGA). The DFT-D3 correction with Becke-Johnson damping was included to account for long-range van der Waals interactions^57^. Based on the in situ XAS results, three representative Fe single-atom models, including Fe-N_3_(PO_X_), Fe-N_2_(PO_X_), and Fe-N_2_, were constructed to describe the PO_X_-assisted coordination reconstruction under NO_3_RR conditions. For computational tractability, the local PO_X_ environment was represented by a simplified model containing a single O center, which was used to capture the local coordination and electronic effects associated with PO_X_. These models are intended to reflect the influence of PO_X_ on the local electronic structure and coordination environment under the simplified computational framework. Fe-N_3_(PO_X_) represents the initial PO_X_-coordinated Fe site, whereas Fe-N_2_(PO_X_) and Fe-N_2_ represent reconstructed Fe coordination environments with different degrees of PO_X_ involvement. A vacuum layer of at least 15 Å was introduced perpendicular to the slab to avoid interactions between periodic images. All structures were fully relaxed until the residual force on each atom was less than 0.02 eV Å^−1^, and the total energy convergence criterion was set to 10^−5^ eV. The plane-wave kinetic energy cutoff was set to 450 eV. Brillouin-zone sampling was performed using a Monkhorst-Pack k-point mesh of 3 × 3 × 1 for geometry optimization and a denser k-point mesh for electronic-structure analysis. Different initial magnetic moments were assigned to the Fe center to identify the most stable spin state for each coordination configuration. The magnetic moments, spin-density distributions, projected density of states (PDOS), d-band centers, Bader charges, charge-density differences, and crystal orbital Hamilton population (COHP) were calculated to evaluate the electronic structure, spin polarization, and Fe-NO_3_ interfacial orbital coupling. Bader charge analysis was performed using the grid-based charge partitioning method, while COHP analysis was carried out using the LOBSTER program. The adsorption free energy of NO_3_RR intermediates was evaluated by placing the adsorbates on the Fe active center and fully relaxing the adsorbate-catalyst systems. The adsorption energy was calculated as Equation (7): *E*_*ads *_*= E*_*total*_ *− E*_*slab*_ − *E*_*X*_, where *E*_total_, *E*_*slab*_, and *E*_*X*_ represent the total energies of the adsorbate-covered catalyst, the clean catalyst, and the isolated adsorbate, respectively^58^. The key intermediates considered in this work included NO_3_*, NO_2_*, NO*, NOH*, N*, NH*, NH_2_*, and NH_3_*. The free-energy change for each elementary step was calculated according to Equation (8): Δ*G* = Δ*E* + Δ*ZPE – T*Δ*S*. Where Δ*E* is the electronic energy difference obtained from DFT calculations, Δ*ZPE* is the zero-point energy correction, *T*Δ*S* is the entropy correction at 298.15 K (Supplementary Table 10). The potential-determining step (PDS) was identified as the elementary step with the largest uphill free-energy change^59^. The atomic coordinates of the optimized computational models are provided in Supplementary Data 1.

## Supplementary information

Supplementary Information

Description of Additional Supplementary Files

Supplementary Data 1

Transparent Peer Review file

## Data Availability

The data that support the conclusions of this study are available within the paper and Supplementary information. Source data are provided with this paper. The source data file has been deposited in Figshare under accession code DOI link^60^.
