# Supplementary material for: Coupled spin-coordination self-adaptation drives efficient nitrate to ammonia conversion
Source: Nat Commun. 2026 Aug 29;17:10334. doi: 10.1038/s41467-026-77163-2 (PMC13623963; doi:10.1038/s41467-026-77163-2)
Supplement: Supplementary file 1 [file 41467_2026_77163_MOESM1_ESM.pdf]

Supplementary Information

Coupled spin-coordination self-adaptation drives efficient nitrate to ammonia conversion

Chun-Kuo Peng[1], Yu-Ru Lin[2], Hsiang-Chun Yu[2], Shih-Ching Huang[2], Yu-Chang Lin[2], Daqin Guan[1], Xiaomin Xu[1], Yingtang Zhou[3], Yung-Jung Hsu[4*], San-Yuan Chen[4,5,6*], Zongping Shao[1*], Yan-Gu Lin[2,4*]

1. *Curtin Centre for Advanced Energy Materials and Technologies (CAEMT), WA School of Mines (WASM), Curtin University, Perth, WA 6102, Australia.*
2. *Scientific Research Division, National Synchrotron Radiation Research Center, Hsinchu 300092, Taiwan.*
3. *Zhejiang Key Laboratory of Pollution Control for Port-Petrochemical Industry, Marine Science and Technology College, Zhejiang Ocean University, Zhoushan, China*
4. *Department of Materials Science and Engineering, National Yang Ming Chiao Tung University, Hsinchu, 300093, Taiwan.*
5. *Graduate Institute of Biomedical Sciences, China Medical University, Taichung, Taiwan*
6. *School of Dentistry, College of Dental Medicine, Kaohsiung Medical University, Kaohsiung, Taiwan*

* Corresponding Authors. *E-mail address:* yhsu@nycu.edu.tw *(Y.J. Hsu),* sanyuanchen@nycu.edu.tw *(S.Y. Chen),* Zongping.Shao@curtin.edu.au *(Z. Shao),* lin.yg@nsrrc.org.tw *(Y.G. Lin)*

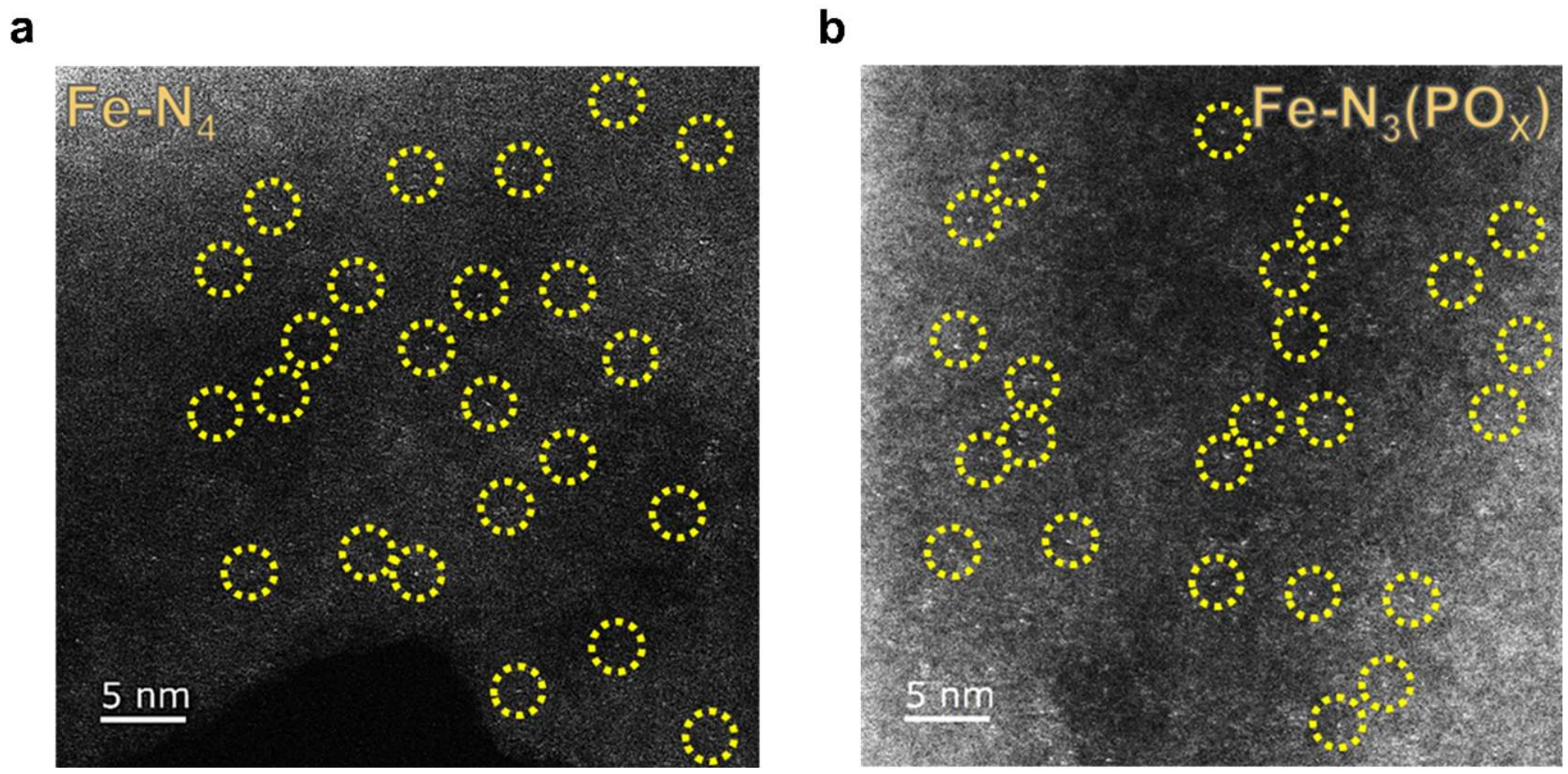

Supplementary Fig. 1. HR-TEM of **a** Fe-N_4 and **b** Fe-N_3(PO_X). The circled bright spots correspond to isolated single atoms.

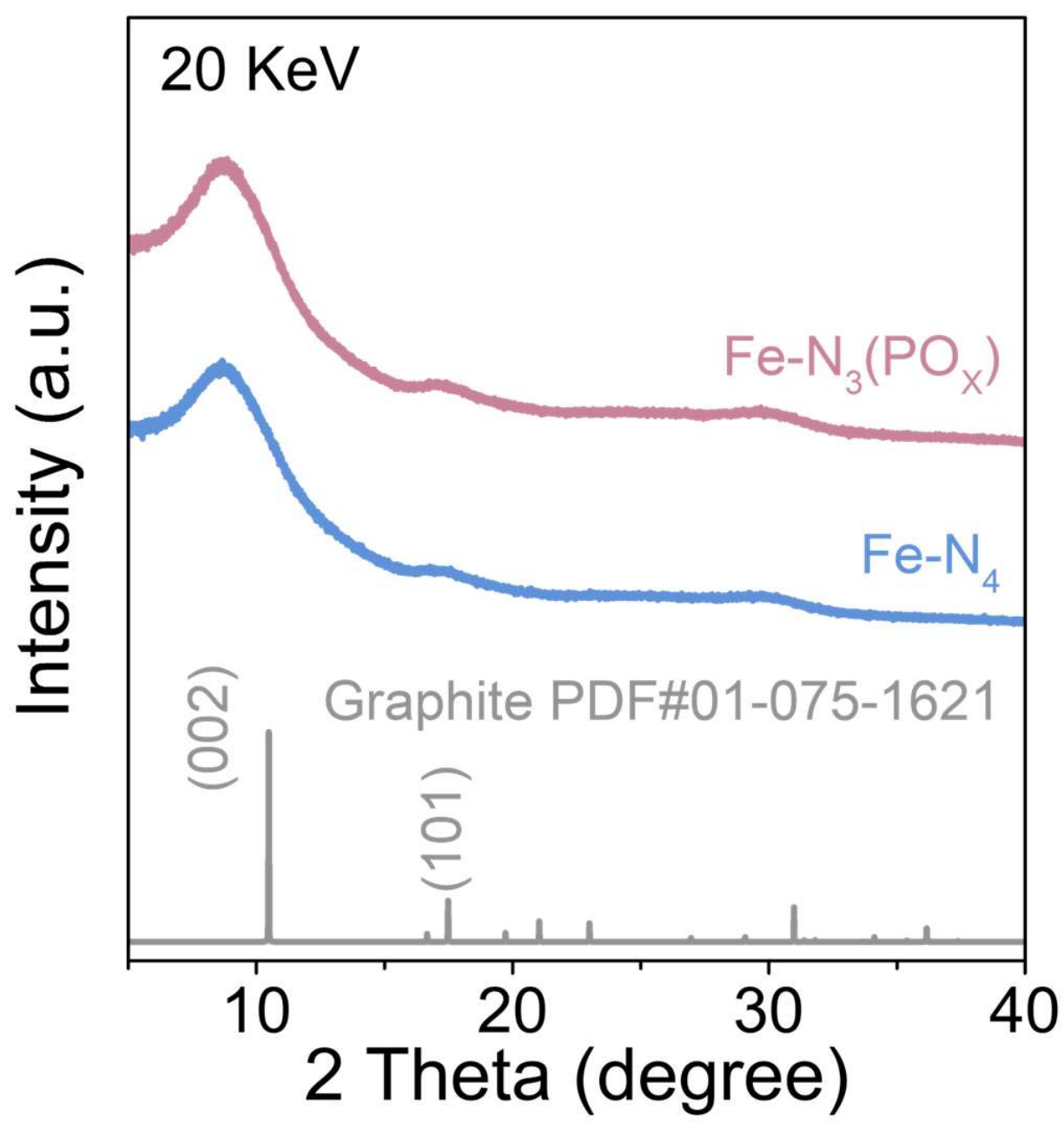

Supplementary Fig. 2. HR-PXRD pattern of $Fe-N_3(PO_X)$ and $Fe-N_4$. Source data are provided as a Source Data file.

Supplementary Table 1. ICP-OES analysis for $Fe-N_4$ and $Fe-N_3(PO_X)$ catalysts.

Catalyst	Chemical composition (wt. %)	
	Fe	P
$Fe-N_4$	0.314	-
$Fe-N_3(PO_X)$	0.215	7.25

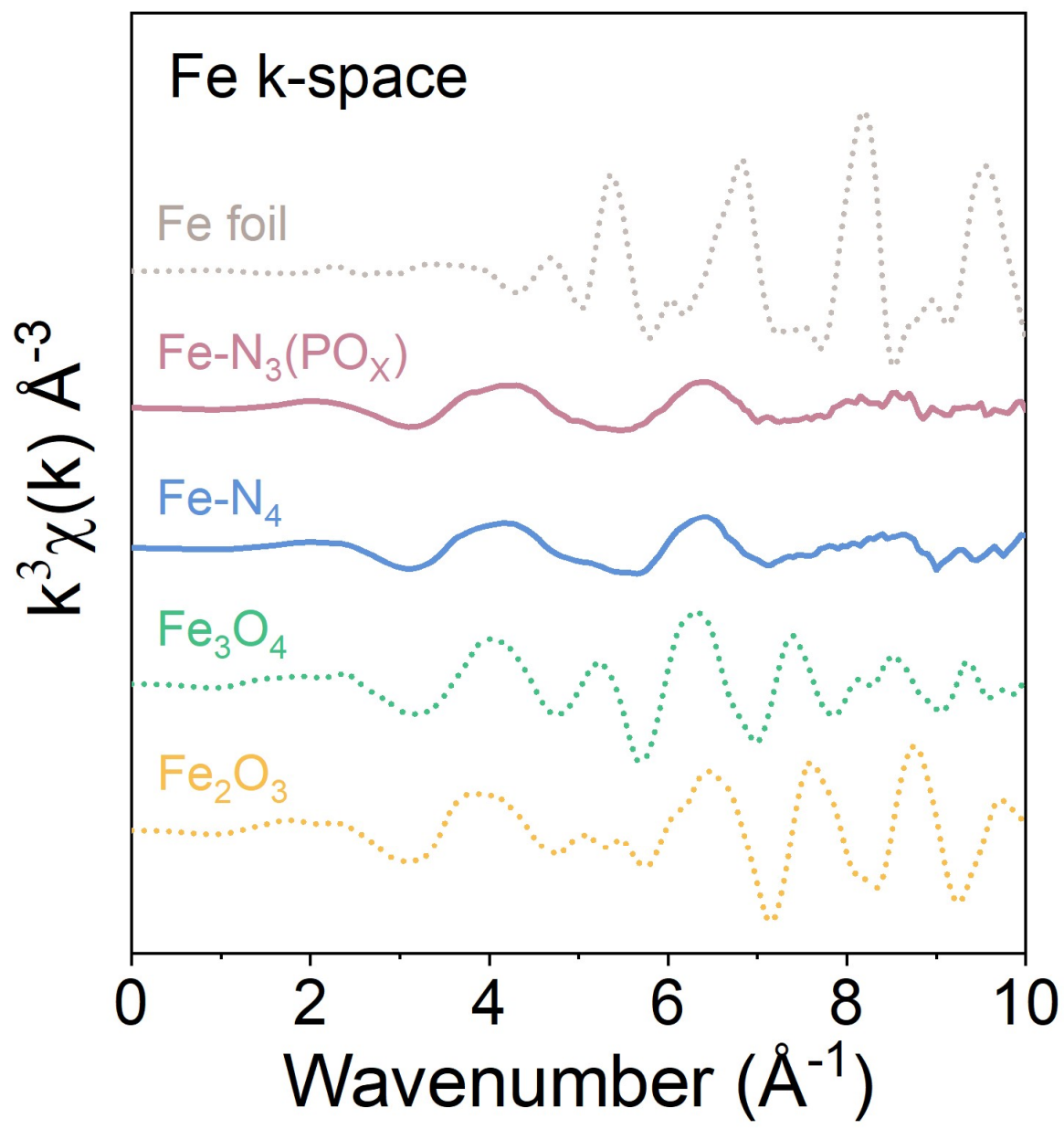

Supplementary Fig. 3. Corresponding k-space of Fe K-edge spectra for $Fe\text{-}N_4$, $Fe\text{-}N_3(PO_X)$, and relevant iron references. Source data are provided as a Source Data file.

Supplementary Note 1

Quantitative EXAFS Curve-Fitting Procedure. EXAFS processing was performed with ATHENA in the Demeter package. The raw absorption data were processed by removing the post-edge background, followed by normalization relative to the edge jump. A k^3-weighting was applied to the resulting $\chi(k)$ signal to amplify oscillations in the high-k region. Finally, the k^3-weighted EXAFS data were Fourier transformed into R-space using a Hanning window (dk = 1 $Å^{-1}$) to resolve the contributions from different coordination shells. To quantitatively determine the local atomic environment, least-squares curve fitting was performed on the k^3-weighted $\chi(k)$ EXAFS data using the ARTEMIS module within the Demeter software package. The data were fitted within a k-space range of 3-9 $Å^{-1}$ and an R-space range of 1-2 Å. The fitting procedure involved refining the coordination number (CN), interatomic distance (R), Debye-Waller factor (σ^2), and edge energy shift (ΔE_0). To ensure consistency across all fits, reference measurements were first conducted using Fe foil, where the coordination number for the first Fe-Fe shell was fixed to the value (CN = 12), and all other parameters were allowed to vary. Based on this reference fitting, the amplitude reduction factor (S_0^2) was evaluated as 0.9 for both systems and subsequently applied to the EXAFS analysis of all other samples. In these sample fittings, CN, R, σ^2, and ΔE_0 were freely optimized without additional constraints. Following this fitting strategy, satisfactory curve-fitting results were obtained, as shown in Fig 1e, Supplementary Fig. 4 and 20. The corresponding fitting parameters are summarized in Supplementary Tables 2, 4 and 5.

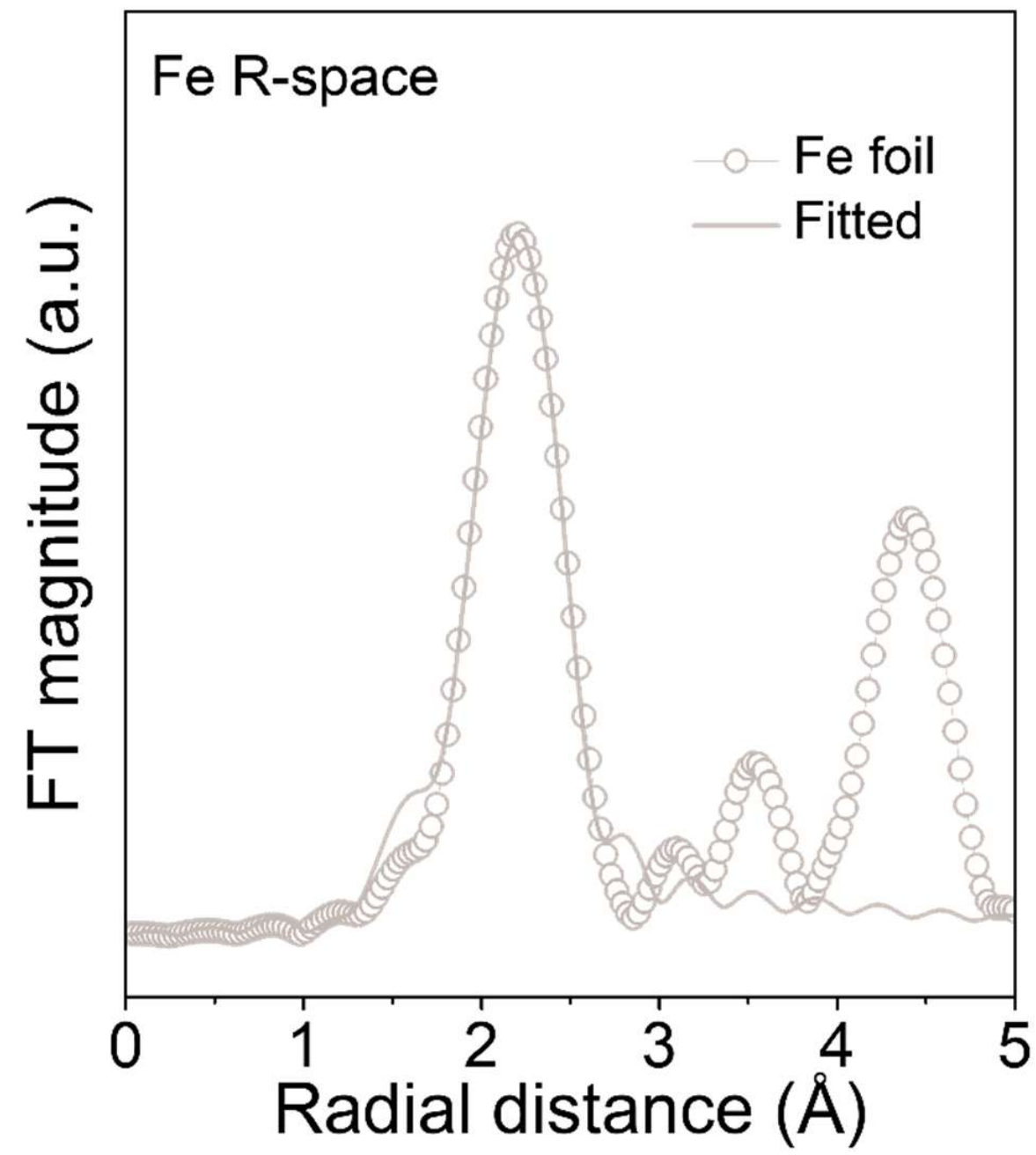

Supplementary Fig. 4. Fe K-edge EXAFS fitting result of Fe foil. Source data are provided as a Source Data file.

Supplementary Table 2. Structural parameters extracted from the Fe K-edge EXAFS fitting of samples.

Sample	Shell	CN	R (Å)	σ^2 (10^{-3} Å^{-2})	ΔE_0 (eV)	*R*-factor
Fe foil	Fe-Fe	12.0±0.9	2.52±0.01	8.0±0.1	7.2±0.8	0.012
$Fe-N_4$	Fe-N	4.0±0.8	1.82±0.01	9.0±0.1	6.9±2.0	0.002
$Fe-N_3(PO_X)$	Fe-N	3.0±0.5	1.81±0.02	8.2±0.1	8.9±1.5	0.001
	Fe-P(O_X)	1.0±0.6	2.18±0.03			

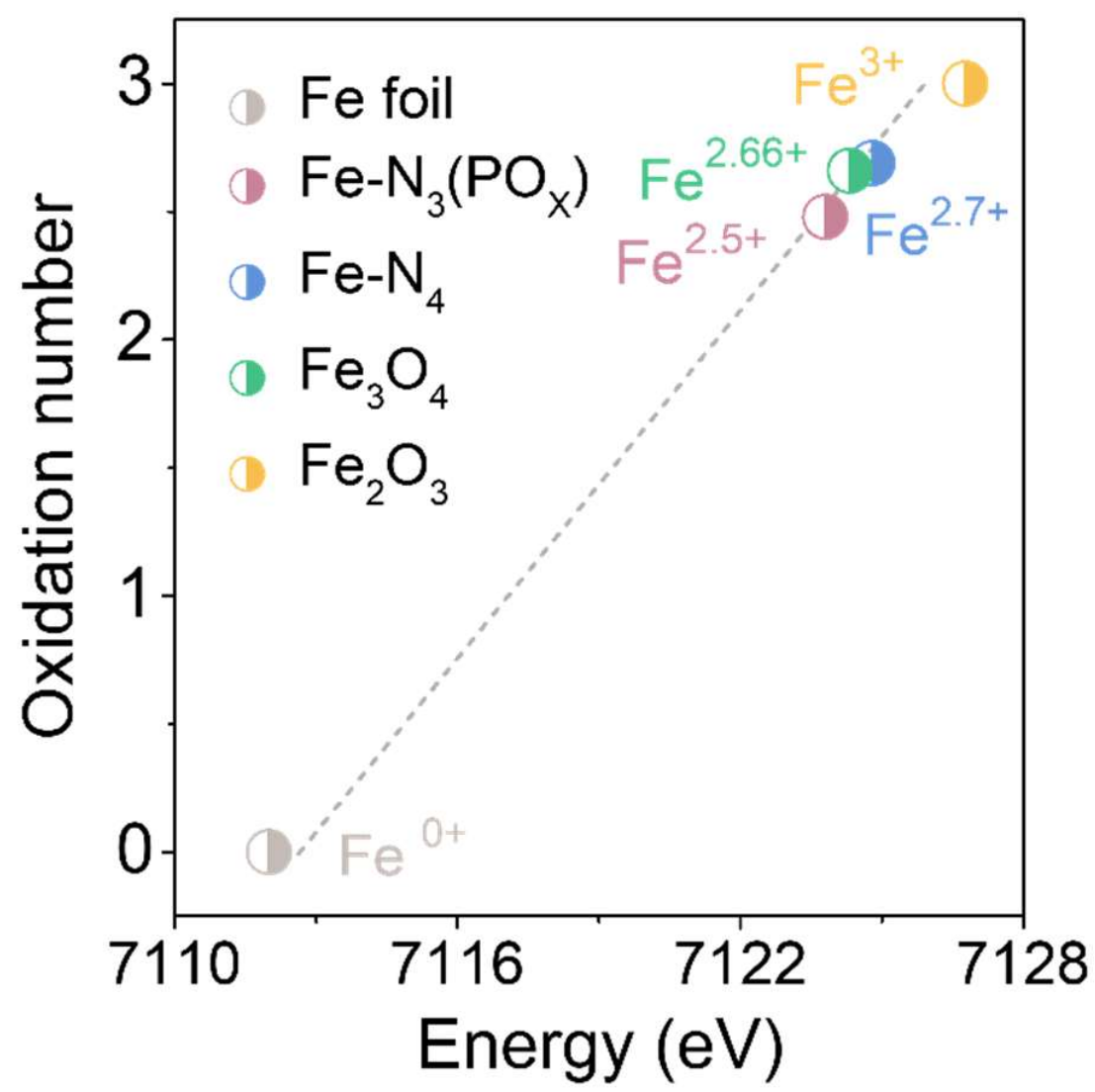

Supplementary Fig. 5. The reference-derived linear regression of Fe valence for $Fe\text{-}N_4$ and $Fe\text{-}N_3(PO_X)$ catalysts. Source data are provided as a Source Data file.

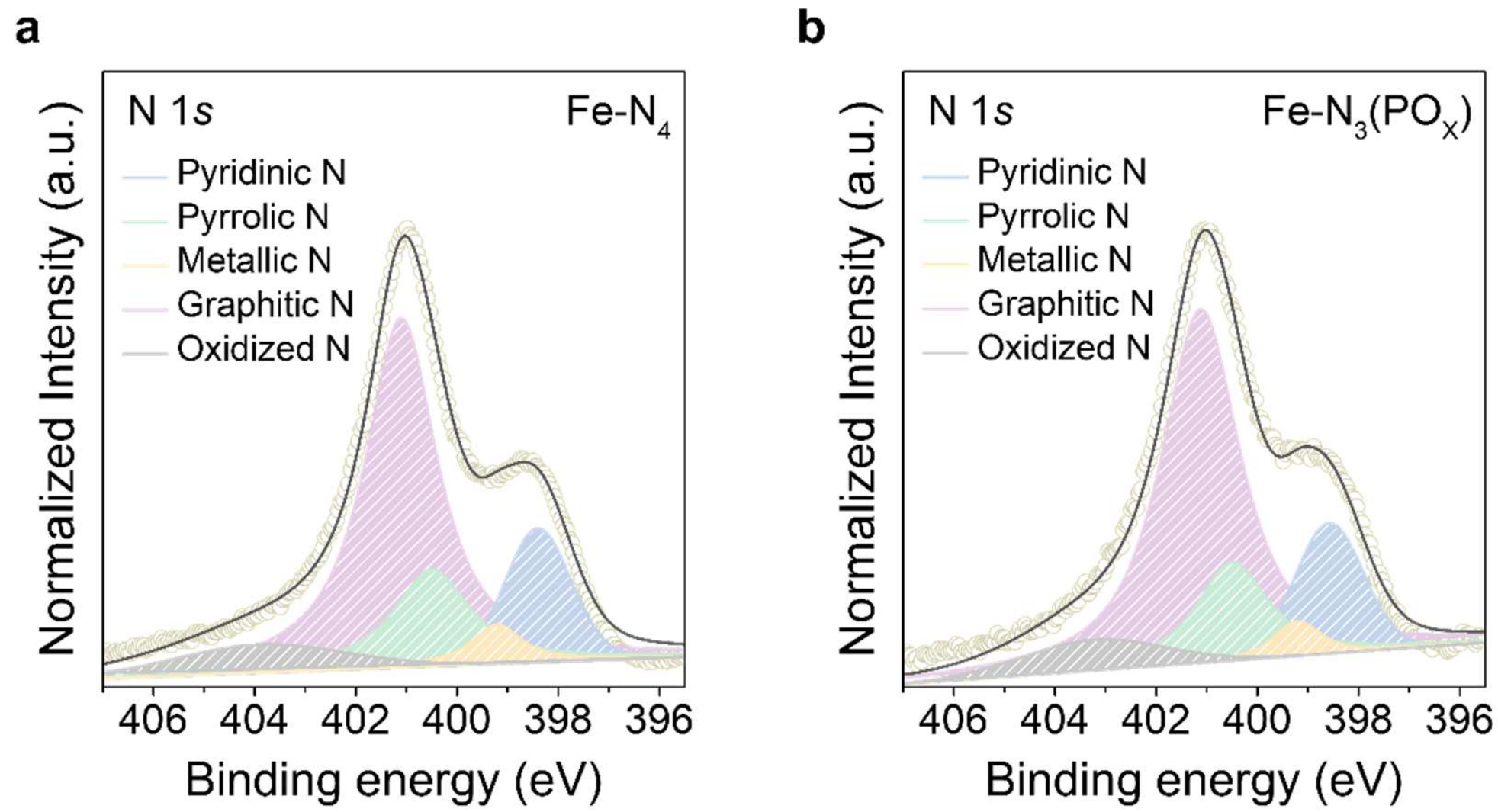

Supplementary Fig. 6. The deconvoluted N 1*s* XPS spectra of **a** $Fe\text{-}N_4$ and **b** $Fe\text{-}N_3PO_X$ catalysts. Source data are provided as a Source Data file.

Supplementary Table 3. Summary of N 1*s* XPS peak components and their relative ratios in $Fe-N_4$ and $Fe-N_3(PO_X)$ catalysts.

Catalyst	Pyridinic N	Metallic N	Pyrrolic N	Graphitic N	Oxidized N
$Fe-N_4$	14.24%	4.54%	16.66%	56.80%	7.76%
$Fe-N_3(PO_X)$	13.86%	3.37%	15.33%	60.70%	6.74%

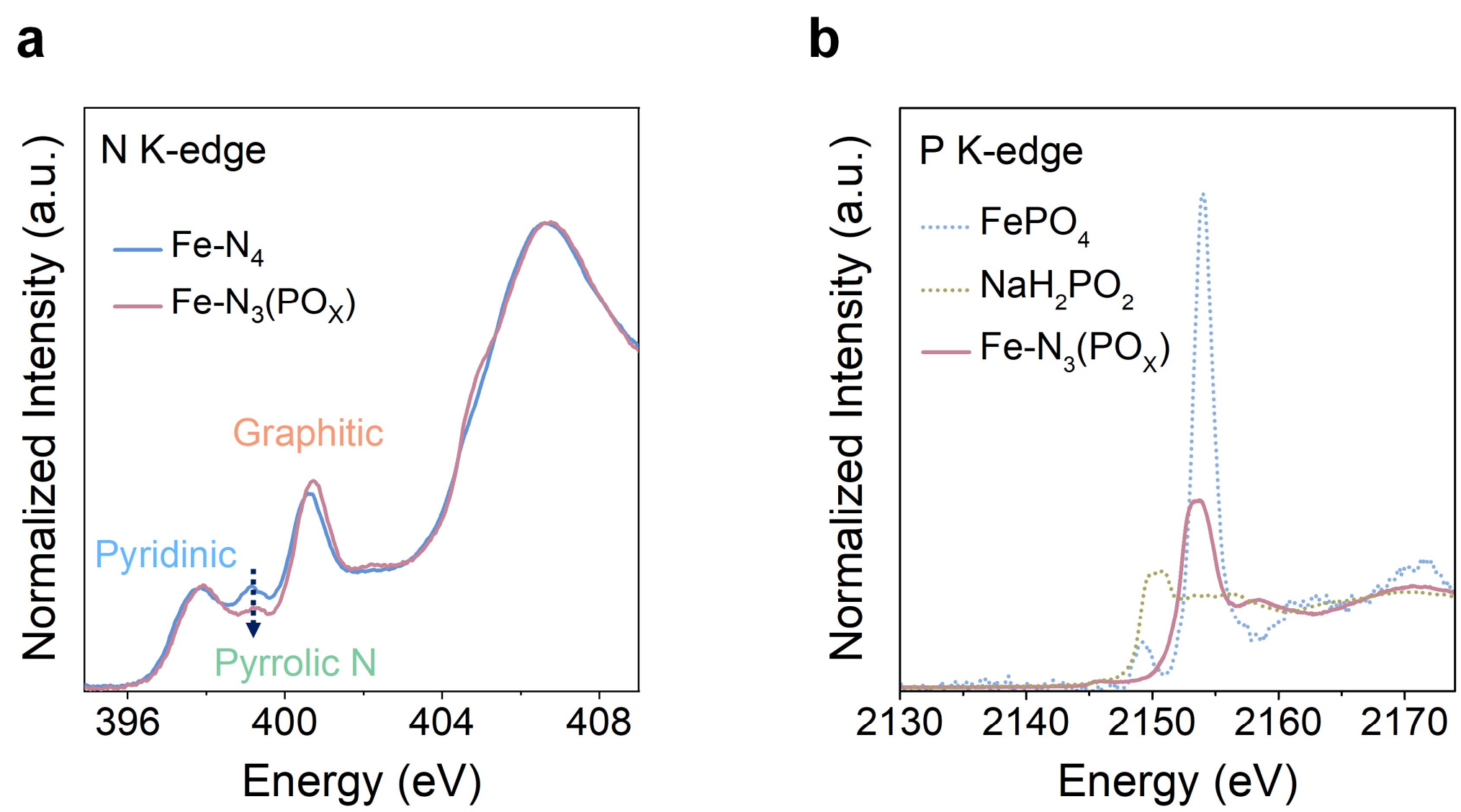

Supplementary Fig. 7. a N K-edge and **b** P K-edge spectra of Fe-N_4 and Fe-$N_3(PO_X)$. Source data are provided as a Source Data file.

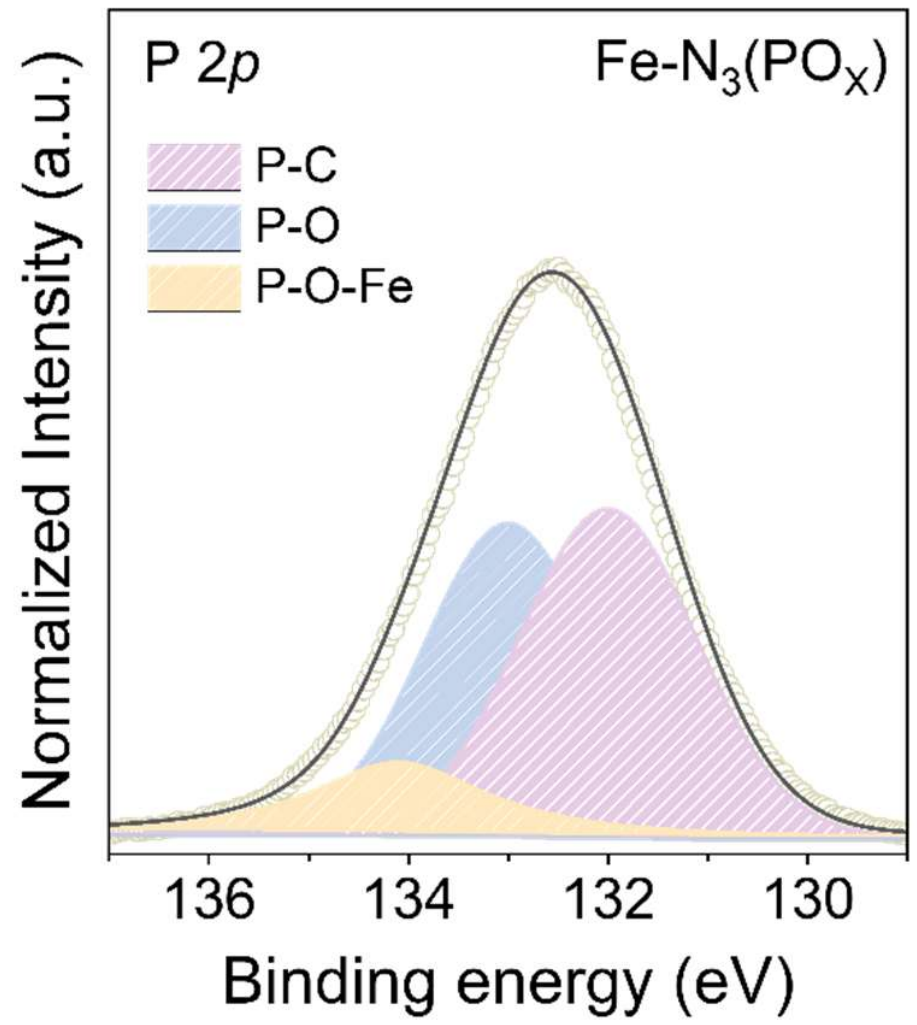

Supplementary Fig. 8. P 2*p* spectrum of Fe-$N_3(PO_X)$. Source data are provided as a Source Data file.

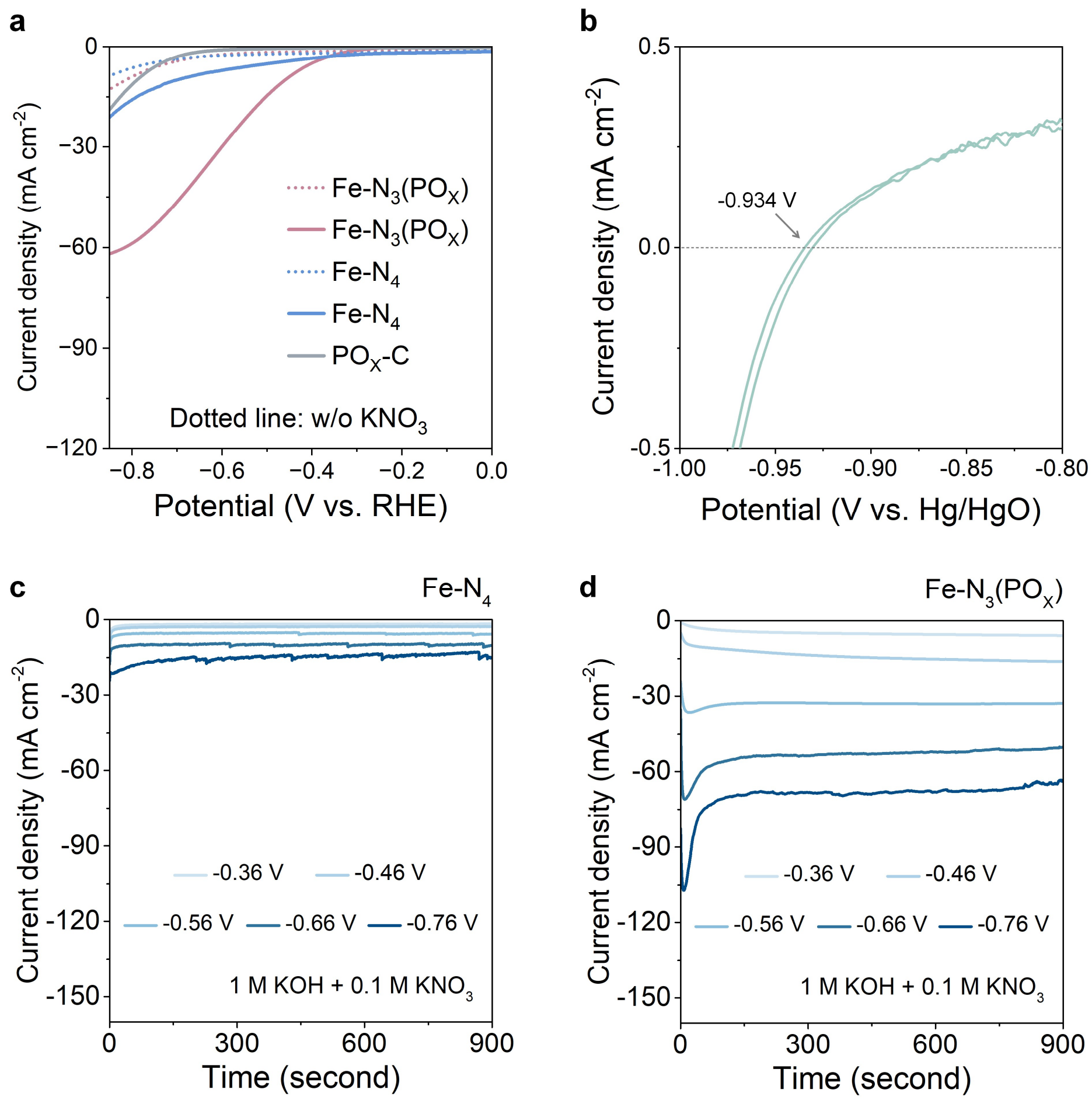

Supplementary Fig. 9. a Linear sweep voltammetry curves of Fe-N_4 and Fe-$N_3(PO_X)$ in 1.0 M KOH with and without 0.1 M KNO_3, and PO_X-C in 1.0 M KOH + 0.1 M KNO_3. Dotted lines indicate KNO_3-free electrolyte, whereas solid lines indicate KNO_3-containing electrolyte. All curves are non-iR-corrected. **b** Calibration of the Hg/HgO reference electrode against RHE using a Pt electrode in H_2 saturated electrolyte. I-t curves of **c** Fe-N_4 and **d** Fe-$N_3(PO_X)$ at various potentials. (Potentials were not iR corrected). Source data are provided as a Source Data file.

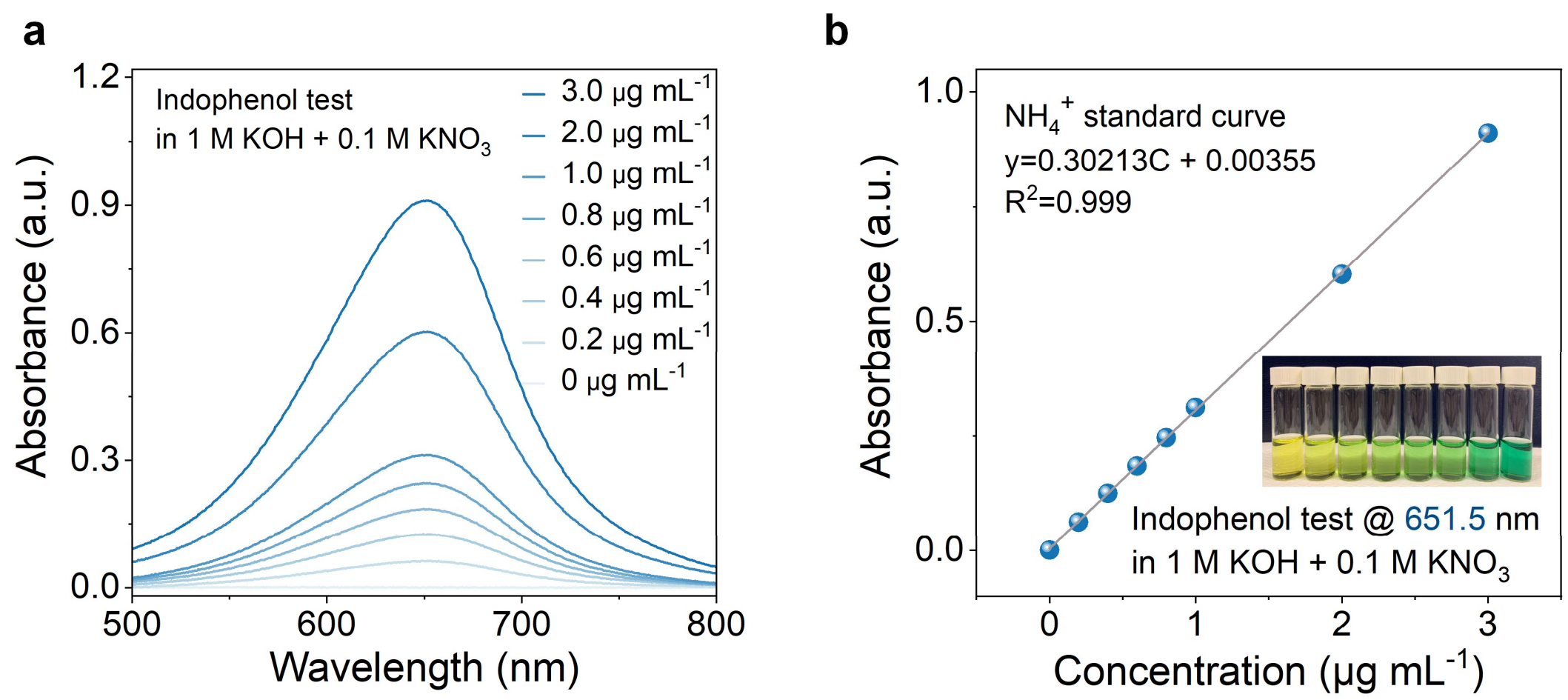

Supplementary Fig. 10. a The UV-vis absorption spectra of indophenol assays for quantitative estimation of NH_4^+ ions concentrations. **b** Linear calibration curve for NH_4^+ quantification based on the indophenol method. The inset shows digital photographs of the colorimetric solutions at the corresponding NH_4^+ concentrations used for the calibration curve, with the concentration increasing from left to right. Source data are provided as a Source Data file.

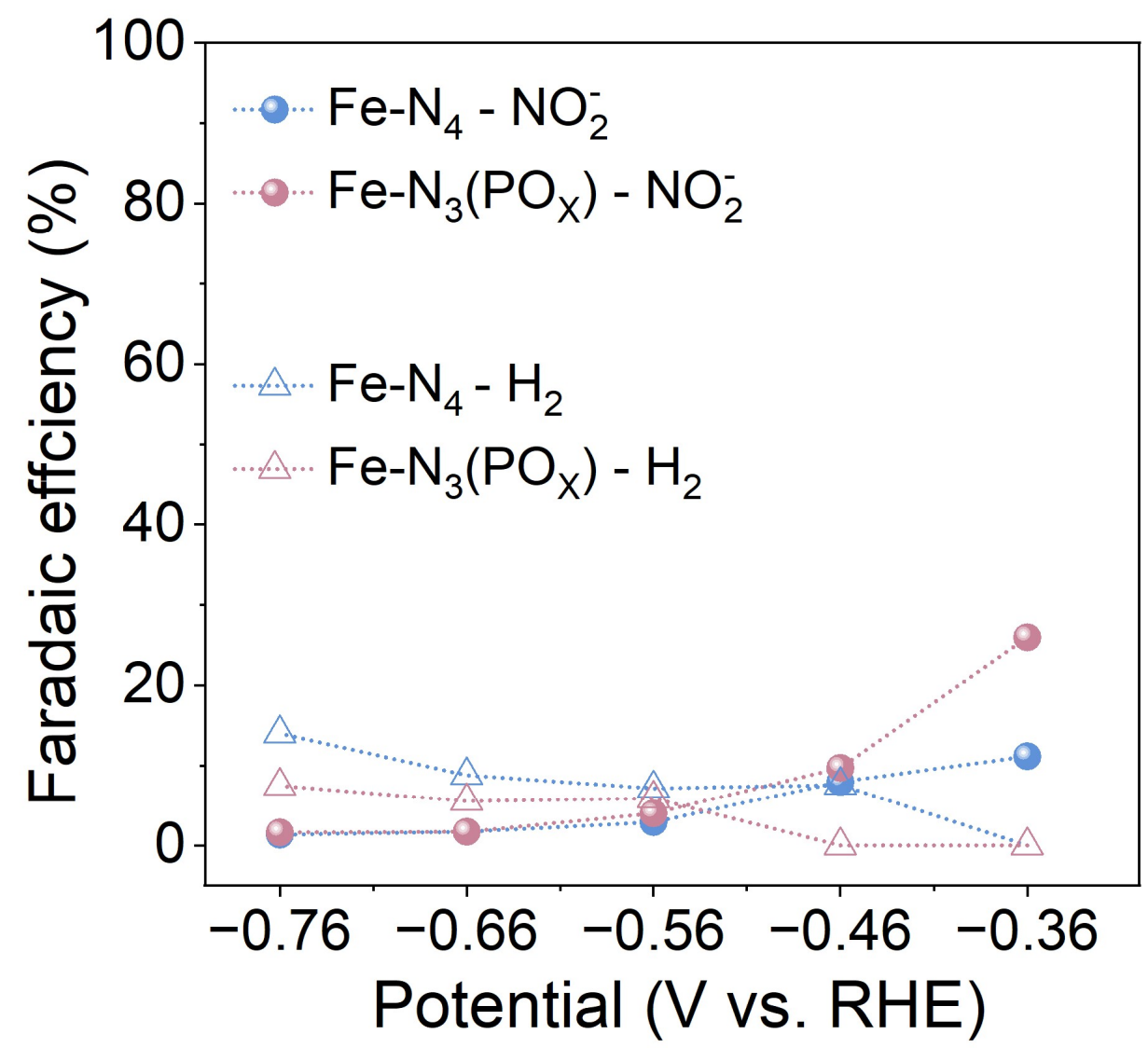

Supplementary Fig. 11. NO_2^- and H_2 Faradaic efficiency for Fe-N_4 and Fe-N_3(PO_X) (potentials were not iR corrected). Source data are provided as a Source Data file.

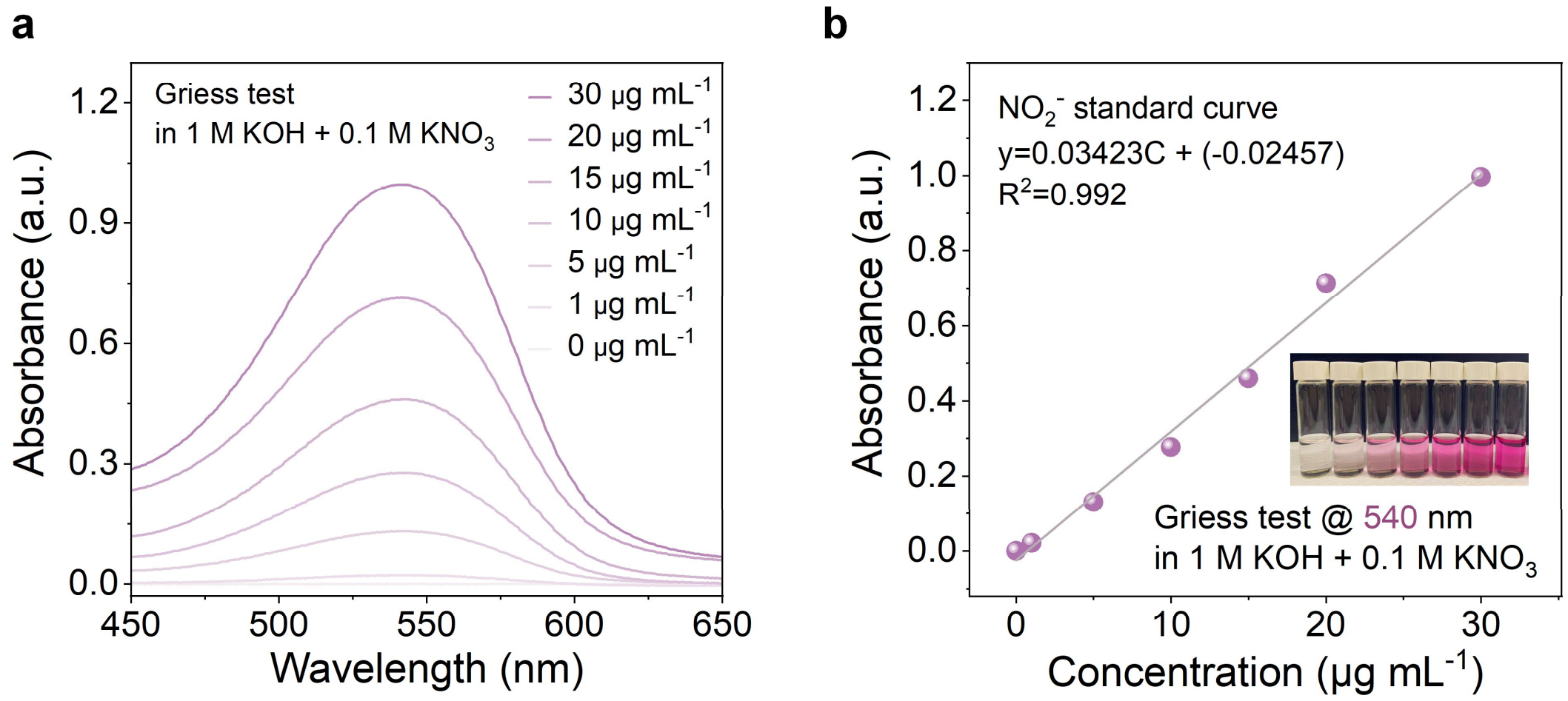

Supplementary Fig. 12. a The UV-vis absorption spectra of Griess assays for quantitative estimation of NO_2^- ions. **b** Linear calibration curve for NO_2^- quantification based on the Griess method. The inset shows digital photographs of the colorimetric solutions at the corresponding NO_2^- concentrations used for the calibration curve, with the concentration increasing from left to right. Source data are provided as a Source Data file.

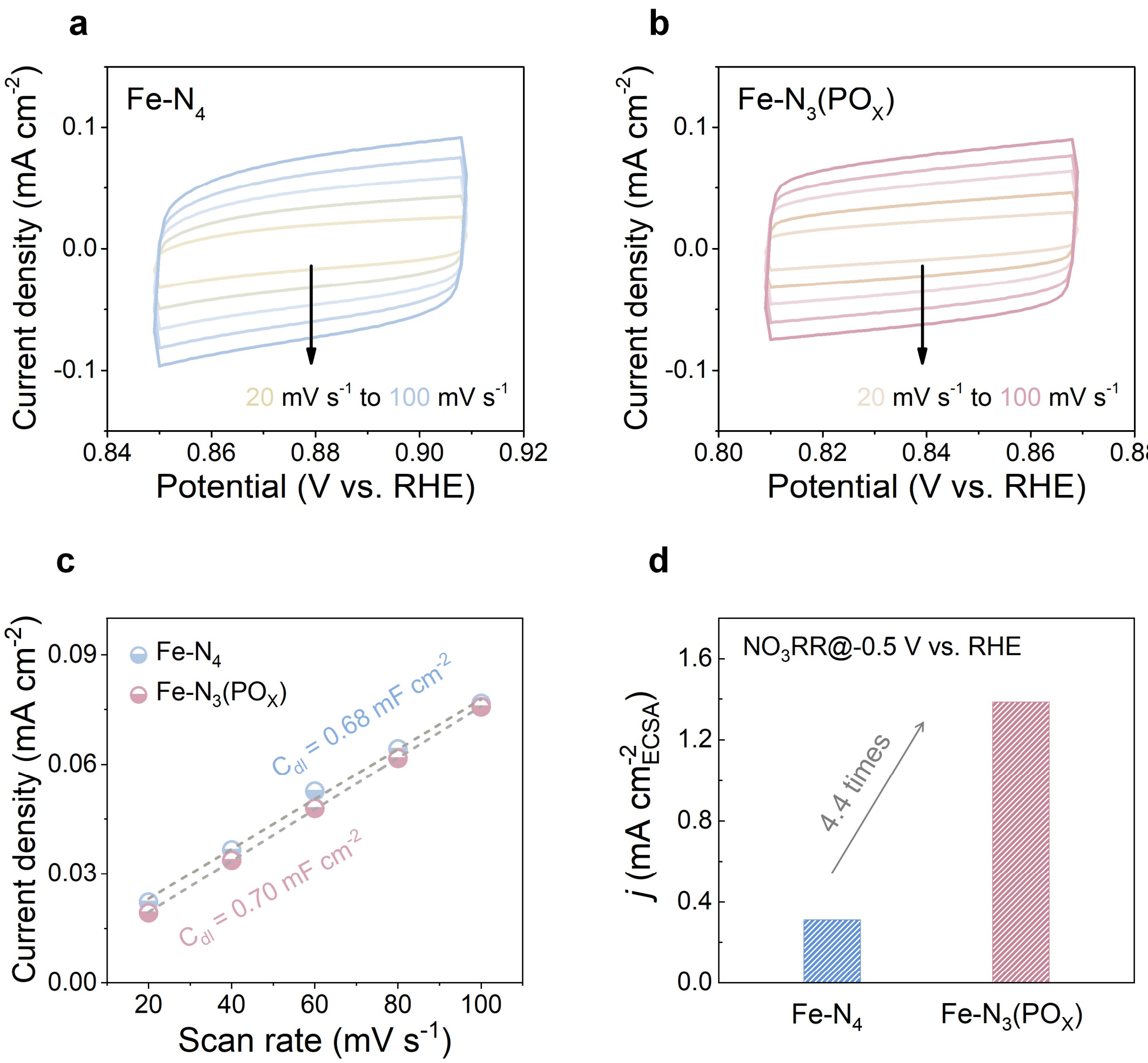

Supplementary Fig. 13. CV curves at varied scan rates for **a** Fe-N_4 and **b** Fe-$N_3(PO_X)$. All the potentials were not iR corrected. **c** Calculated electrochemical double-layer capacitance. **d** ECSA normalized current density recorded at -0.5 V vs. RHE. Source data are provided as a Source Data file.

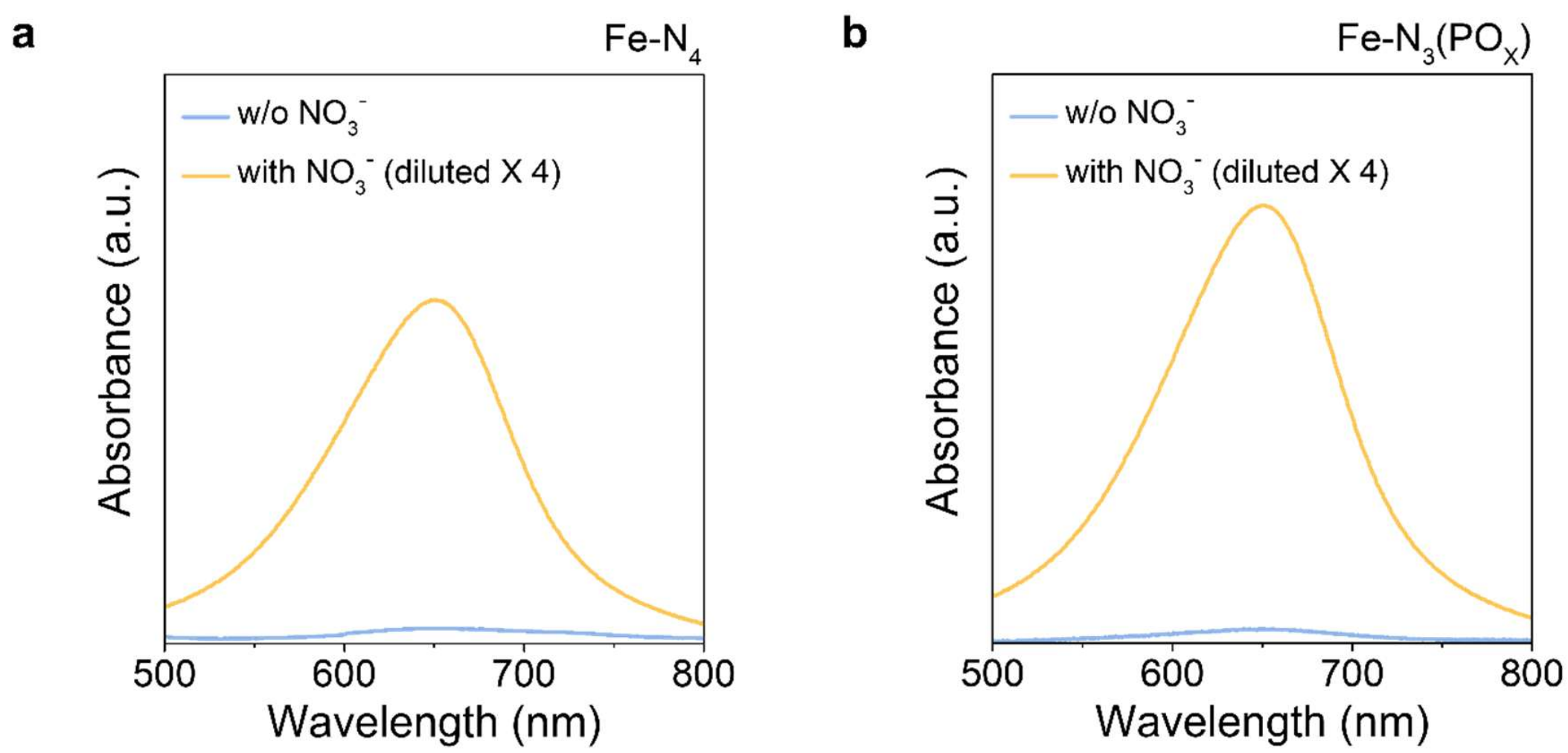

Supplementary Fig. 14. Indophenol assays were conducted for **a** Fe-N_4 and **b** Fe-$N_3(PO_X)$ in 1.0 M KOH, with and without the addition of 0.1 M KNO_3. Source data are provided as a Source Data file.

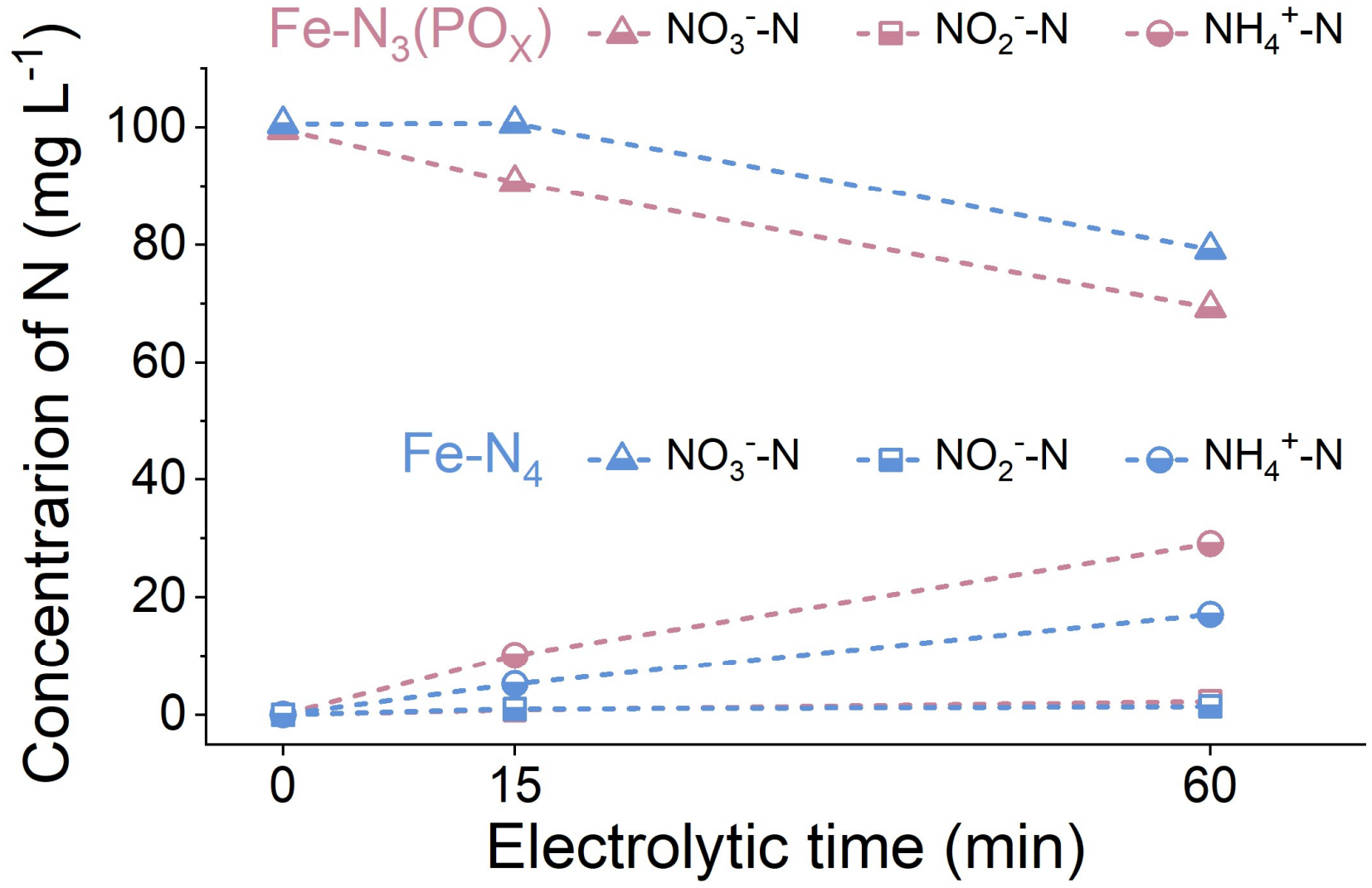

Supplementary Fig. 15. Time-dependent concentration changes of NO_3^-, NO_2^- , and NH_4^+ at -0.46 V for Fe-N_4 and Fe-N_3(PO_X). Source data are provided as a Source Data file.

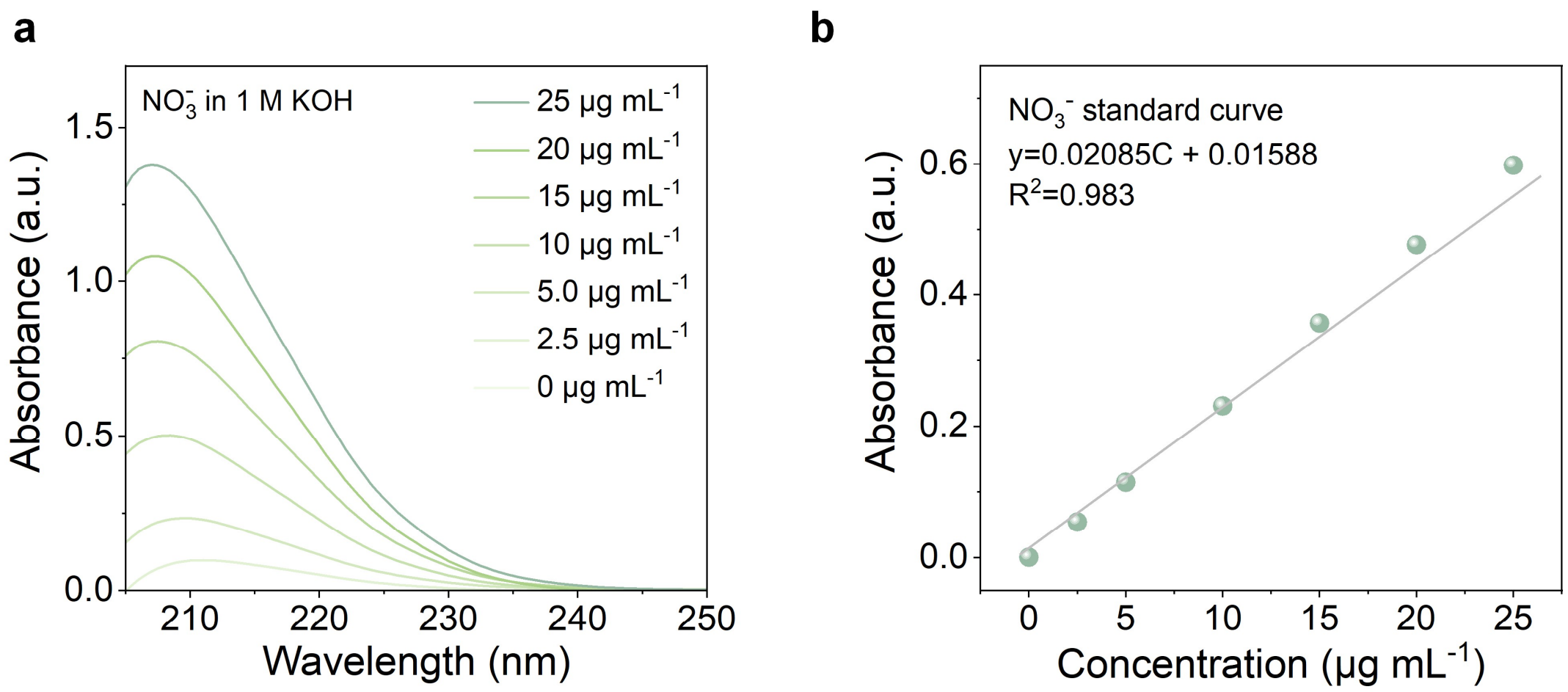

Supplementary Fig. 16. a UV-vis standard curve for NO_3^- standard solutions. **b** Linear Calibration curve for NO_3^- quantification. Source data are provided as a Source Data file.

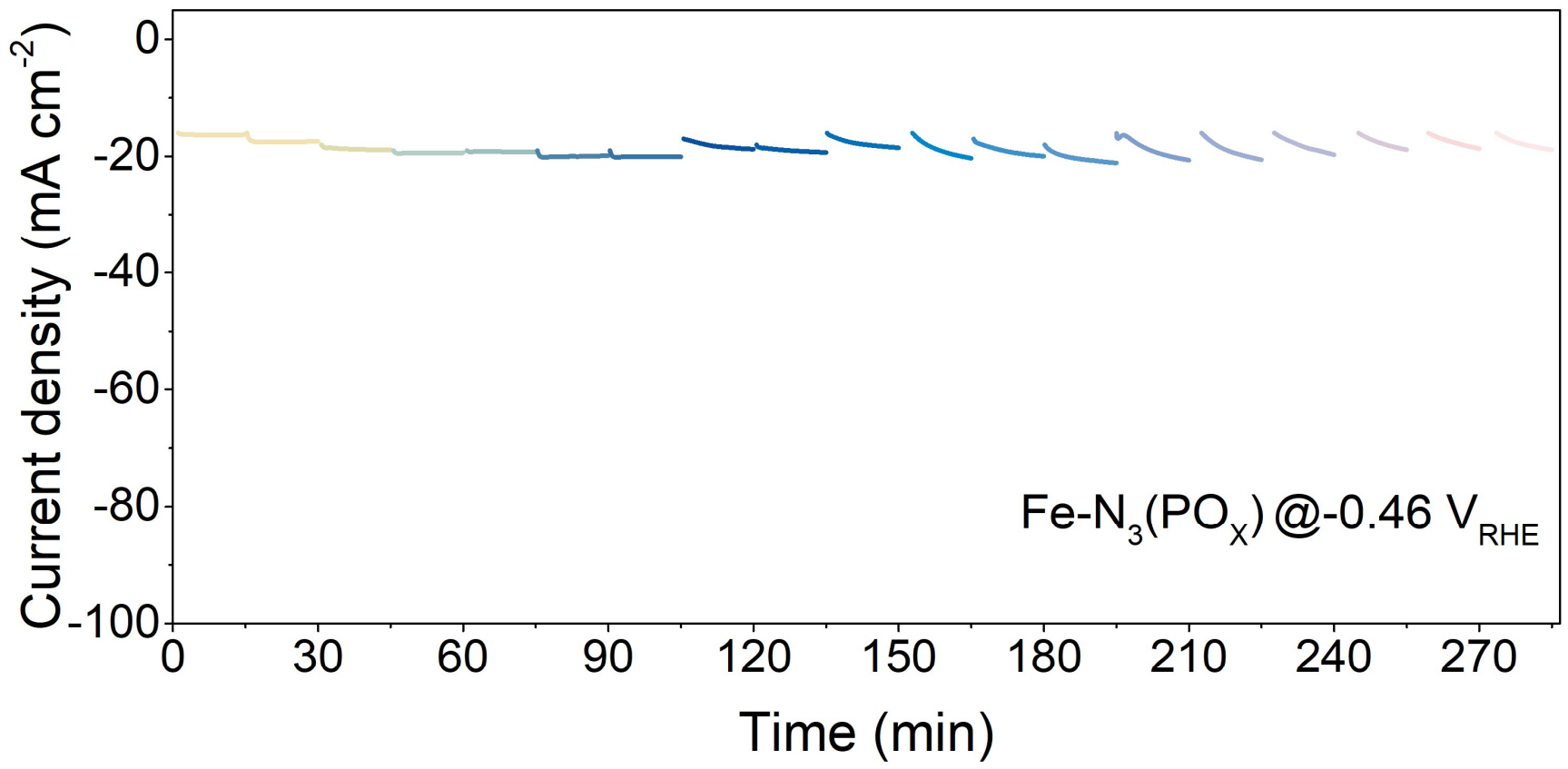

Supplementary Fig. 17. Chronoamperometry curves of Fe-N_3(PO_X) at -0.46 V during 19 periods of 0.25 h electrocatalysis (without iR correction). Source data are provided as a Source Data file.

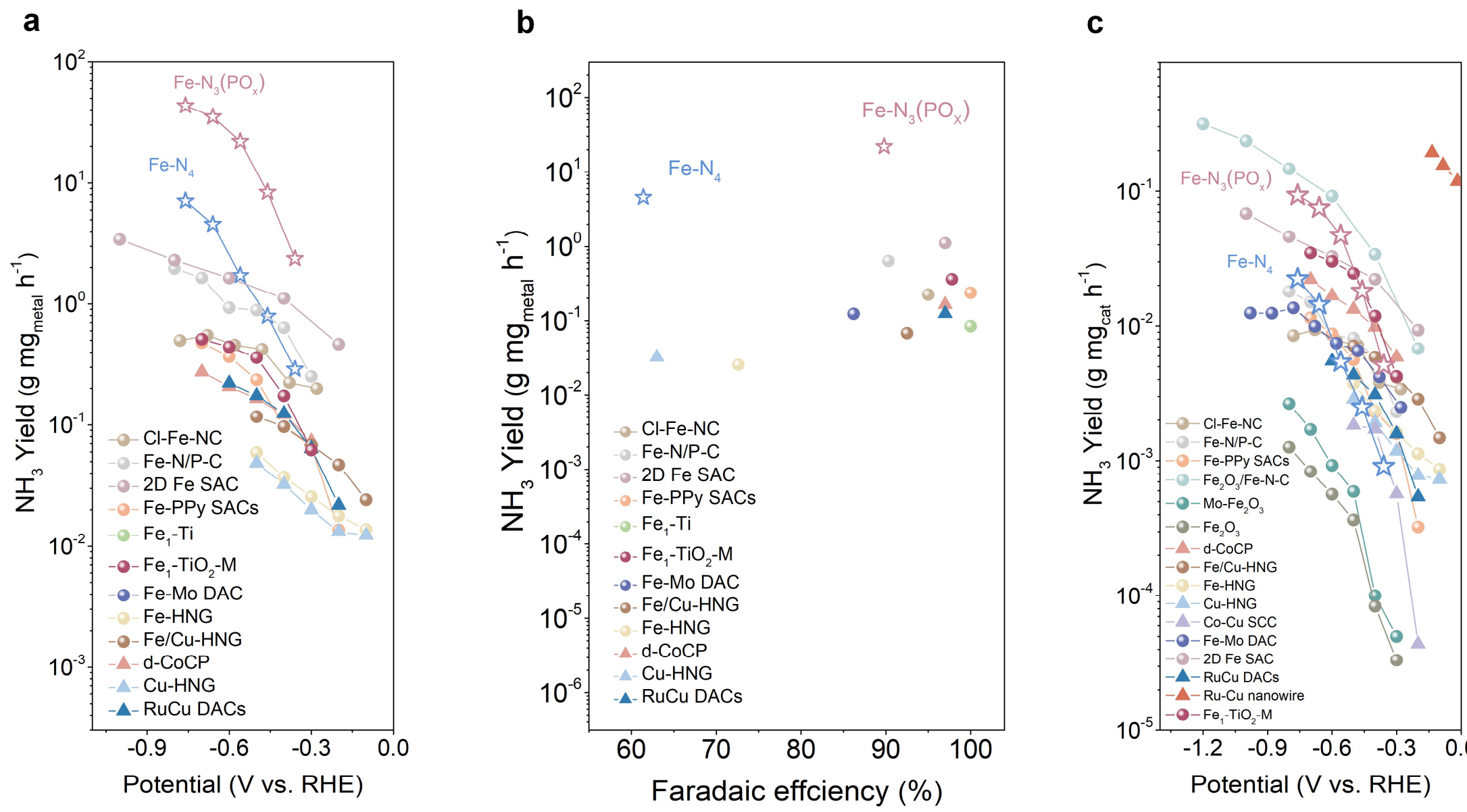

Supplementary Fig. 18 **a** Performance comparison of Fe-N_3(PO_X) electrode with other reported NO_3RR single-atom electrocatalysts. **b** Comparison of NH_3 yield rate and Faradaic efficiency for NO_3RR over Fe-N_3(PO_X), Fe-N_4, and recently reported single-atom catalysts. **c** Performance comparison of the Fe-N_3(PO_X) electrode with representative NO_3RR electrocatalysts based on the ammonia yield rate normalized to the total catalyst mass.

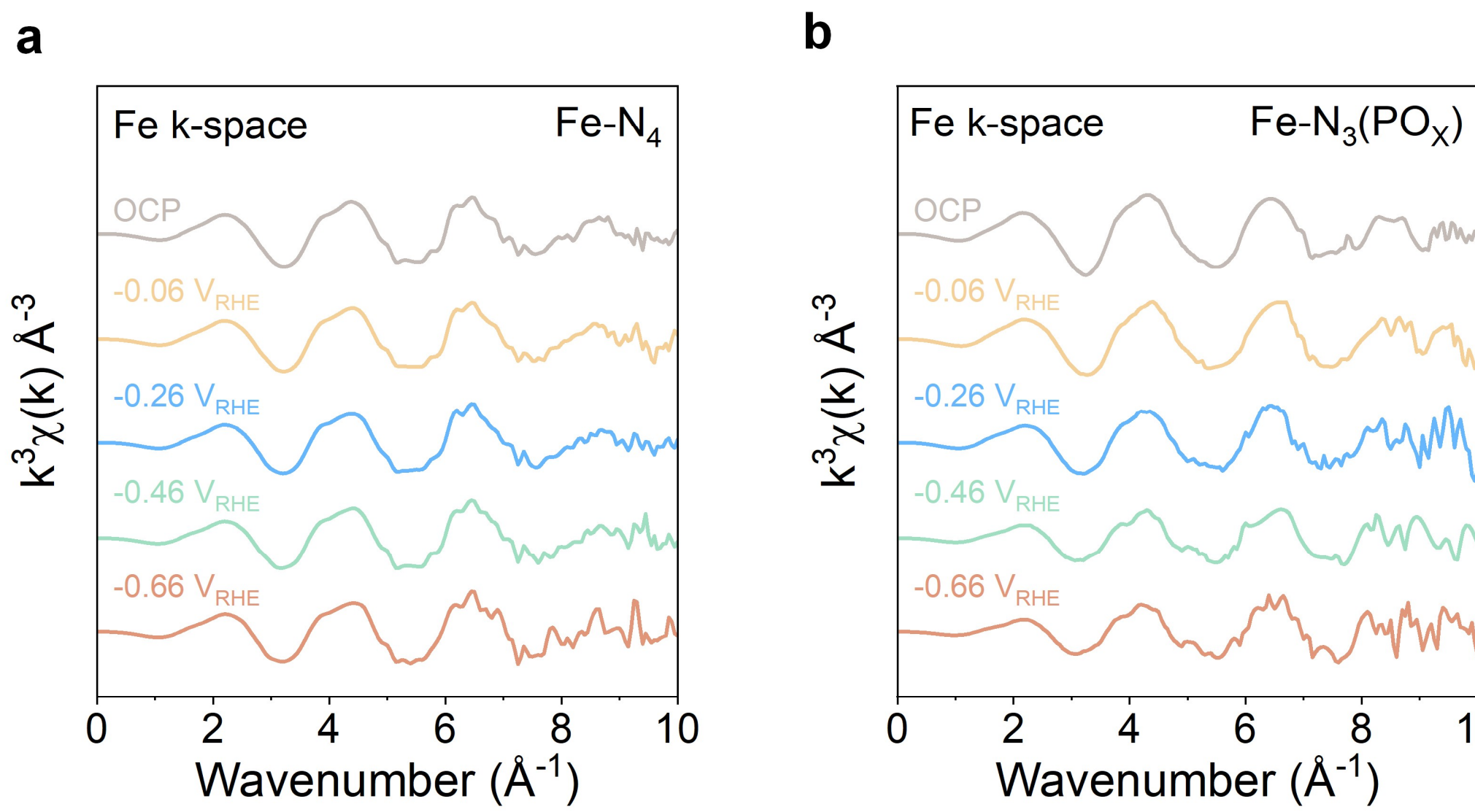

Supplementary Fig. 19. Corresponding *in-situ* k-space EXAFS spectra oscillation of **a** Fe-N_4 and **b** Fe-$N_3(PO_X)$ during NO_3RR. All the potentials were not iR corrected. Source data are provided as a Source Data file.

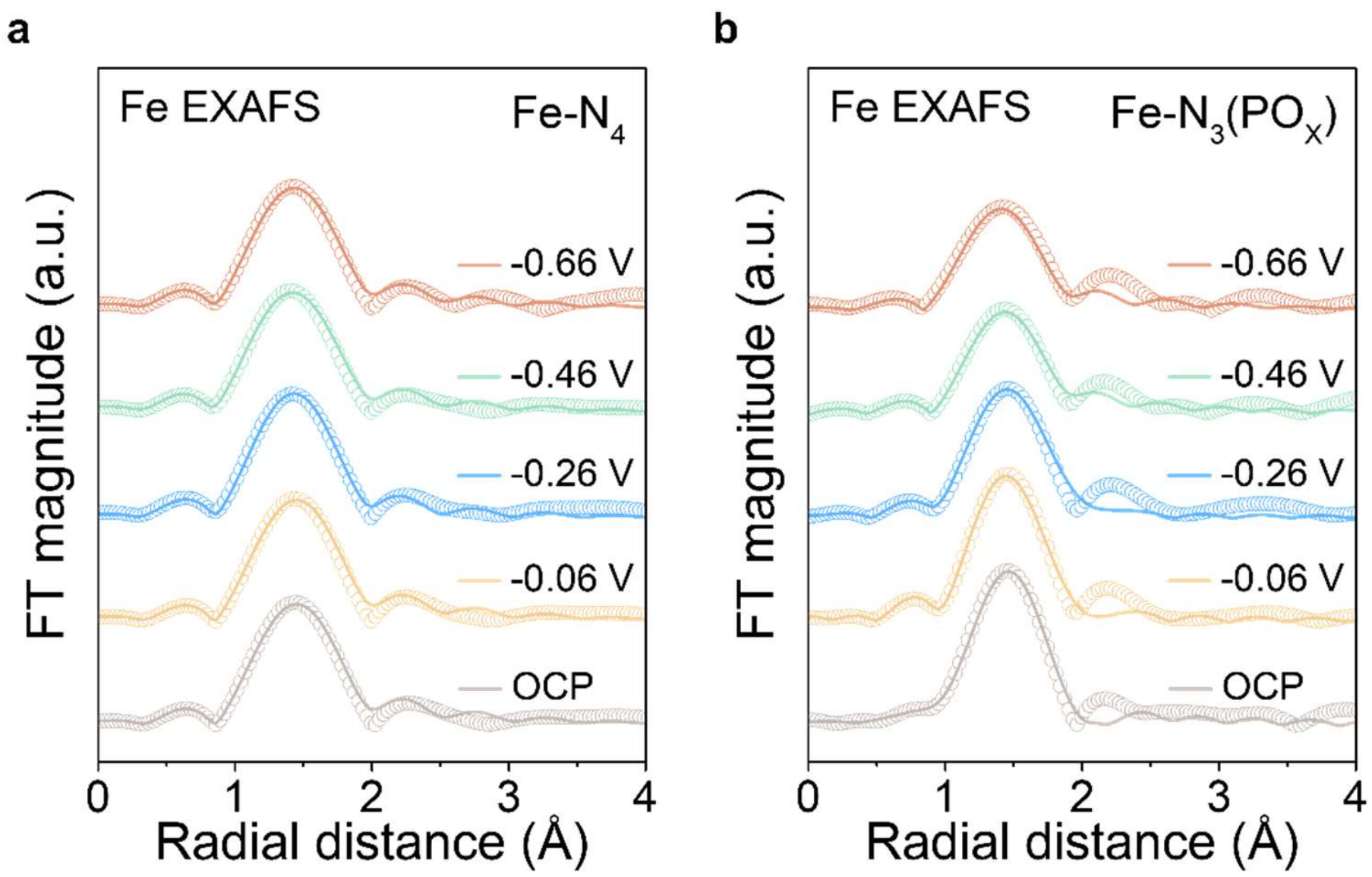

Supplementary Fig. 20. Fe K-edge EXAFS fitting curves recorded from OCP to -0.66 V. The detailed results of the fits are listed in Supplementary Table 4 and 5. All the potentials were not iR corrected. Source data are provided as a Source Data file.

Supplementary Table 4. Summary of parameters extracted from the *in-situ* Fe-K-edge EXAFS fitting of Fe-$N_3(PO_X)$ in NO_3RR.

Sample	Shell	CN	R (Å)	σ^2 (10^{-3} Å^{-2})	ΔE_0 (eV)	*R*-factor
OCP	Fe-N	3.0 ±0.6	1.81 ±0.02	8.5±0.1	8.2±1.9	0.004
	Fe-P(O_X)	1.0 ±0.4	2.18 ±0.06			
-0.06 V	Fe-N	2.6 ±0.4	1.81 ±0.02	7.8±0.1	8.7±1.5	0.001
	Fe-P(O_X)	1.0 ±0.4	2.18 ±0.05			
-0.26 V	Fe-N	2.2 ±0.5	1.81 ±0.02	7.0±0.1	8.0±2.0	0.003
	Fe-P(O_X)	1.0 ±0.4	2.18 ±0.05			
-0.46 V	Fe-N	2.2 ±0.4	1.84 ±0.02	5.6 ±0.1	8.7 ±2.1	0.003
-0.66 V	Fe-N	2.0 ±0.5	1.84 ±0.03	5.4 ±0.1	8.5 ±2.1	0.010

Supplementary Table 5. Summary of parameters extracted from the *in-situ* Fe-K-edge EXAFS fitting of Fe-N_4 in NO_3RR.

Sample	Shell	CN	R (Å)	σ^2 (10^{-3} Å^{-2})	ΔE_0 (eV)	*R*-factor
OCP	Fe-N	4.0 ±0.7	1.82 ±0.02	8.5 ±0.1	7.0 ±2.3	0.004
-0.06 V	Fe-N	4.0 ±0.7	1.82 ±0.02	8.4 ±0.1	6.8 ±2.0	0.001
-0.26 V	Fe-N	4.0 ±1.0	1.82 ±0.02	8.6 ±0.1	6.2 ±2.6	0.003
-0.46 V	Fe-N	4.0 ±0.8	1.81 ±0.02	8.2 ±0.1	5.8 ±2.0	0.003
-0.66 V	Fe-N	4.0 ±0.8	1.82 ±0.02	8.3 ±0.1	6.3 ±2.0	0.010

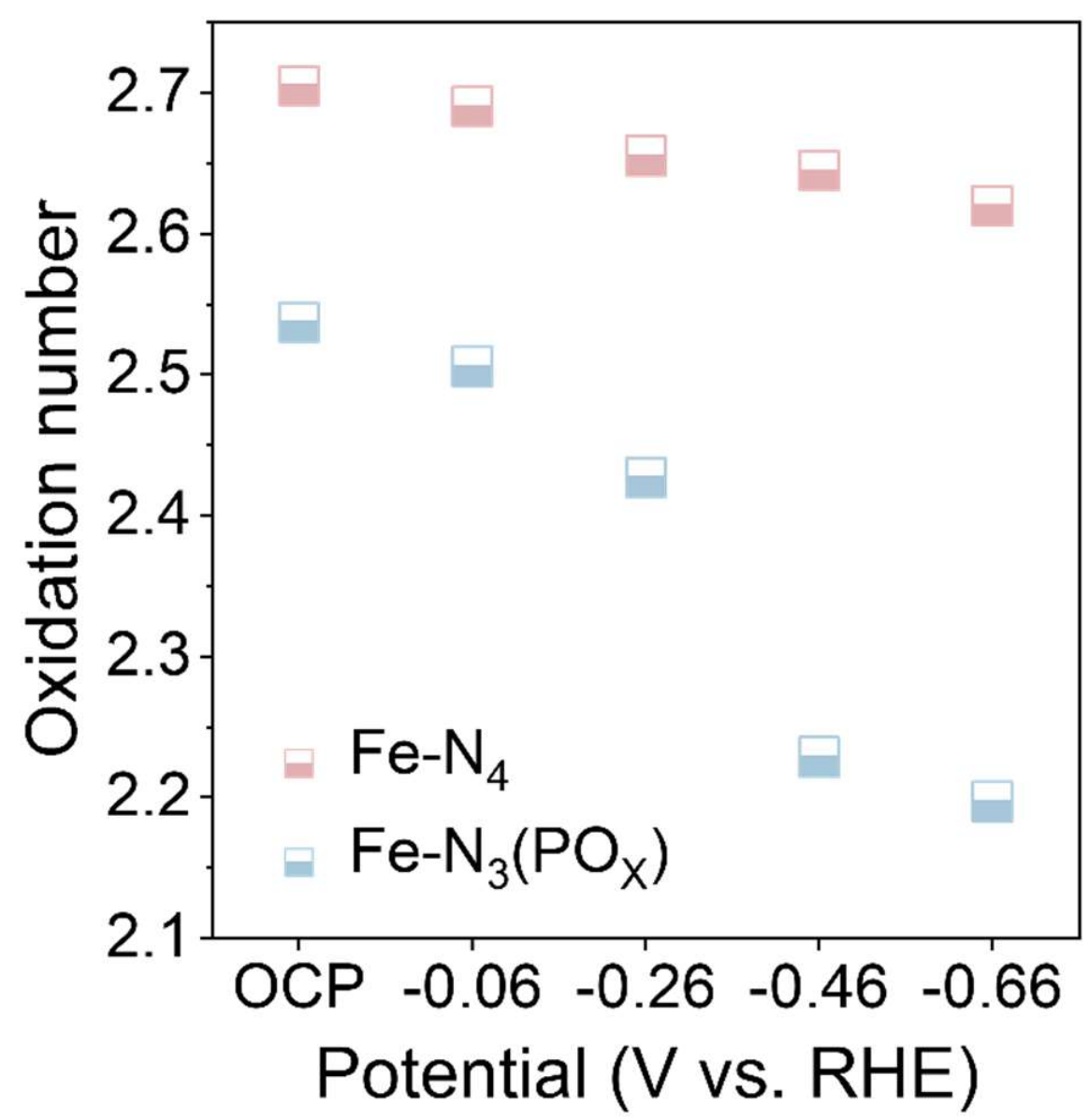

Supplementary Fig. 21. The Fe valence state changes of Fe-N_4 and Fe-N_3(PO_X) during *in-situ* XAS measurements. All the potentials were not iR corrected. Source data are provided as a Source Data file.

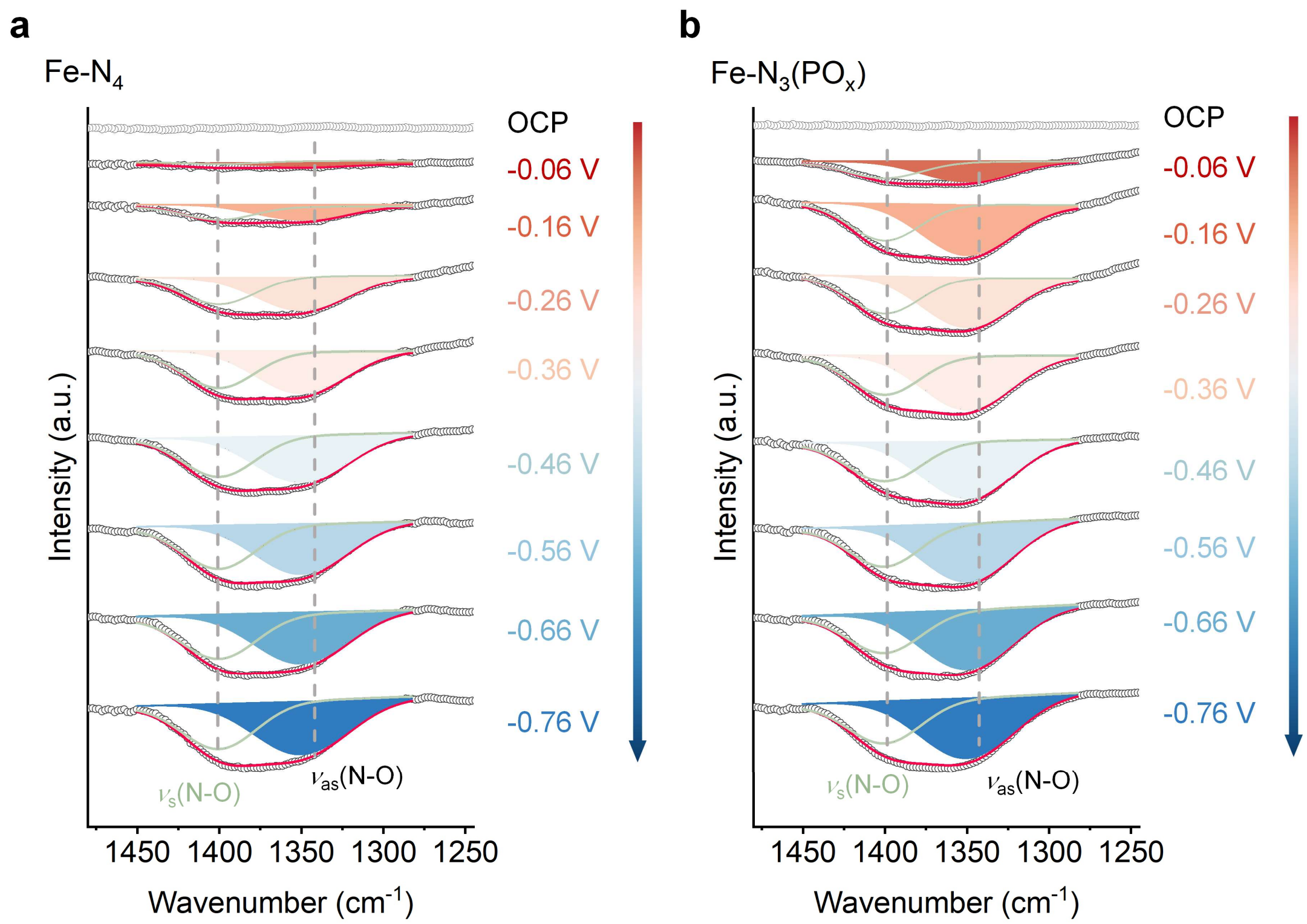

Supplementary Fig. 22. *In-situ* ATR-FTIR fitting spectra for **a** Fe-N_4 and **b** Fe-$N_3(PO_X)$. The NO_3^- stretching bands are deconvoluted into symmetric and asymmetric peak. All the potentials were not iR corrected. Source data are provided as a Source Data file.

Supplementary Table 6. Symmetric and asymmetric peak of NO_3^- stretching band areas, extracted from *in-situ* ATR-FTIR spectra of Fe-N_4 and Fe-N_3(PO_X) in the range 1300-1450 cm^{-1}.

Sample	Fe-N_4		Fe-N_3(PO_X)	
	$Area_{asym}$	$Area_{sym}$	$Area_{asym}$	$Area_{sym}$
-0.06 V	0.018	0.012	0.119	0.074
-0.16 V	0.088	0.058	0.235	0.135
-0.26 V	0.194	0.127	0.292	0.165
-0.36 V	0.248	0.158	0.314	0.170
-0.46 V	0.282	0.183	0.340	0.180
-0.56 V	0.303	0.195	0.345	0.178
-0.66 V	0.312	0.200	0.354	0.182
-0.76 V	0.324	0.212	0.368	0.190

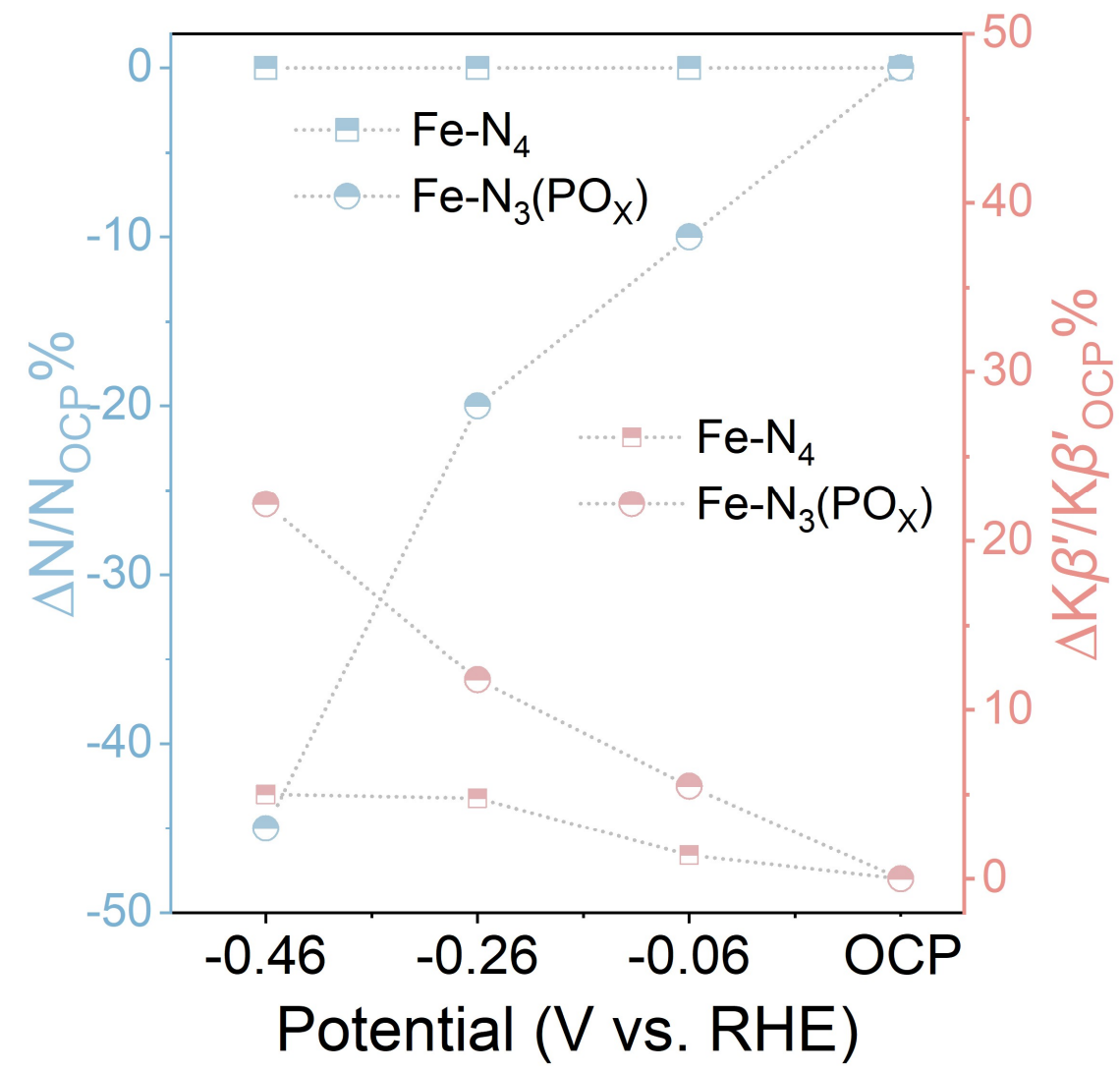

Supplementary Fig. 23. Dynamic evolution of total coordination number and spin state for $Fe\text{-}N_4$ and $Fe\text{-}N_3(PO_X)$ during NO_3RR. All the potentials were not iR corrected. Source data are provided as a Source Data file.

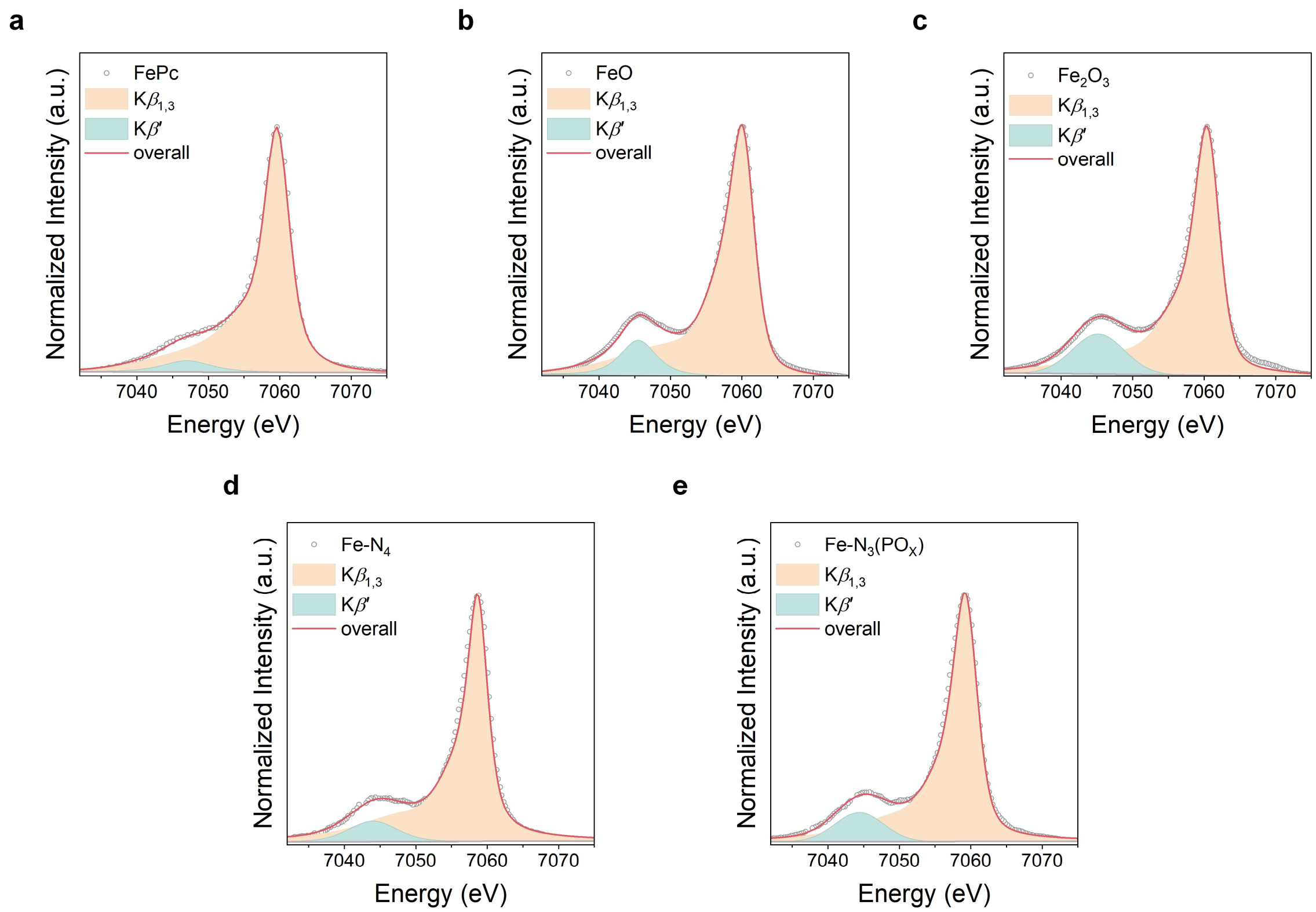

Supplementary Fig. 24. Gaussian fitting of Fe Kβ X-ray emission spectra of **a** iron phthalocyanine (FePc) reference, **b** FeO reference, **c** Fe_2O_3 reference, **d** Fe-N_4 and **e** Fe-$N_3(PO_X)$. The experimental spectra were fitted using Gaussian functions to deconvolute the Kβ' satellite and K$\beta_{1,3}$ main emission features. All the potentials were not iR corrected. Source data are provided as a Source Data file.

Supplementary Table 7. Fitting results of Fe Kβ emission spectra of the reference compounds, Fe-N_4 and Fe-N_3(PO_X). The spectra were fitted using four Gaussian functions to extract the K$\beta_{1,3}$, Kβ', and total Kβ peak areas.

	K$\beta_{1,3}$ area	Kβ' area	Relative Kβ area (%)	Average spin state
FePc	5.34	0.51	6.33	1
FeO	5.75	1.21	12.77	2
Fe_2O_3	6.11	1.48	15.48	2.5
Fe-N_4	5.80	0.82	9.30	1.51
Fe-N_3(PO_X)	5.90	1.02	11.23	1.82

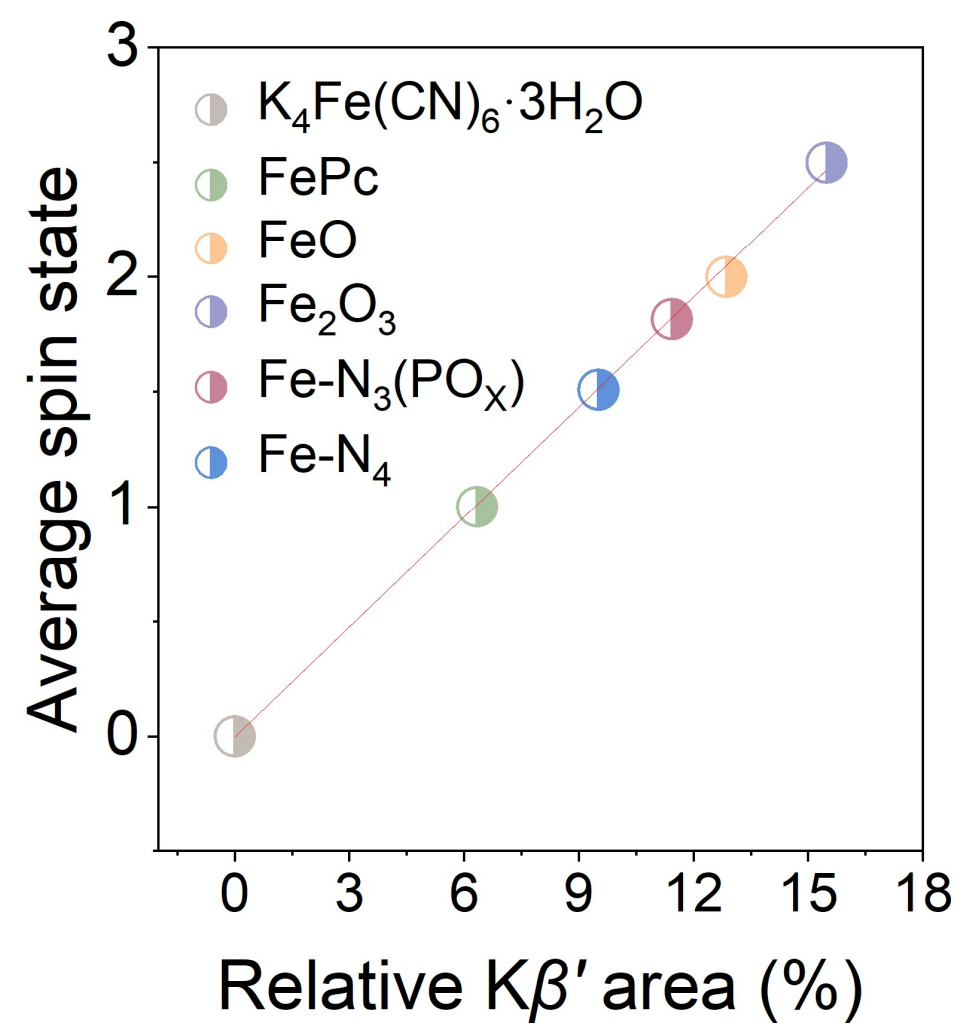

Supplementary Fig. 25. Linear correlation between the relative Kβ' peak area and the average spin state of the reference compounds with Fe-N_4 and Fe-N_3(PO_X) positioned on the fitted calibration curve for spin-state evaluation. Source data are provided as a Source Data file.

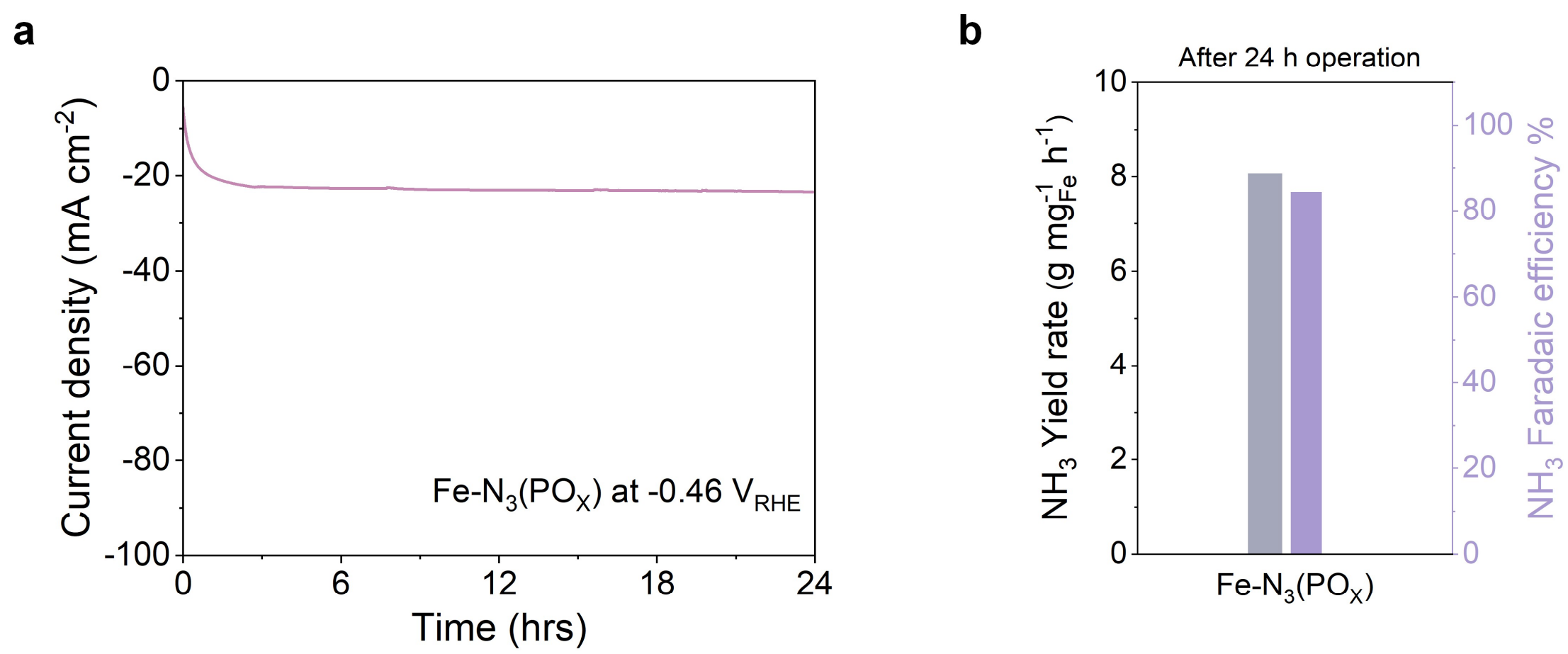

Supplementary Fig. 26. a chronoamperometry stability test for 24 hours at -0.46 V vs. RHE (without iR correction) **b** corresponding NH_3 yield rate and Faradaic efficiency after 24 h of stability test. Source data are provided as a Source Data file.

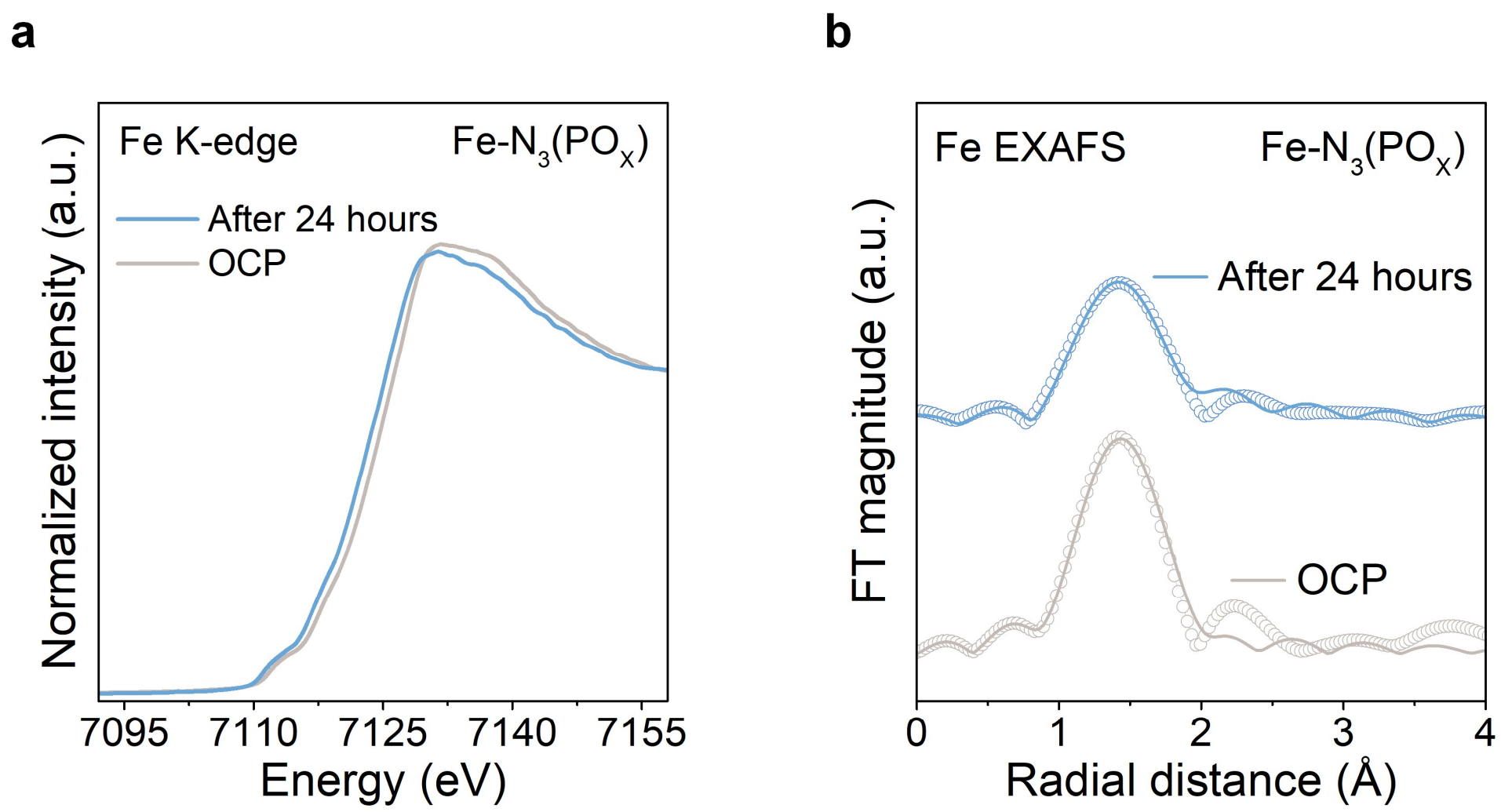

Supplementary Fig. 27. a XANES and **b** EXAFS spectra of Fe-$N_3(PO_X)$ collected before and after 24 h stability testing at -0.46 V vs. RHE (without iR correction). Source data are provided as a Source Data file.

Supplementary Table 8. Summary of structural parameters extracted from Fe K-edge EXAFS fitting of Fe-N_3(PO_X) before and after the NO_3RR test.

Sample	Shell	CN	R (Å)	σ^2 (10^{-3} Å^{-2})	ΔE_0 (eV)	*R*-factor
OCP	Fe-N	3.0 ±0.5	1.81 ±0.03	7.0±0.1	11.36±1.5	0.003
	Fe-P(O_X)	1.0 ±0.5	2.18 ±0.07			
After 24 hours @-0.46 V	Fe-N	2.1±0.1	1.84 ±0.06	3.9 ±0.1	10.37±5.9	0.011

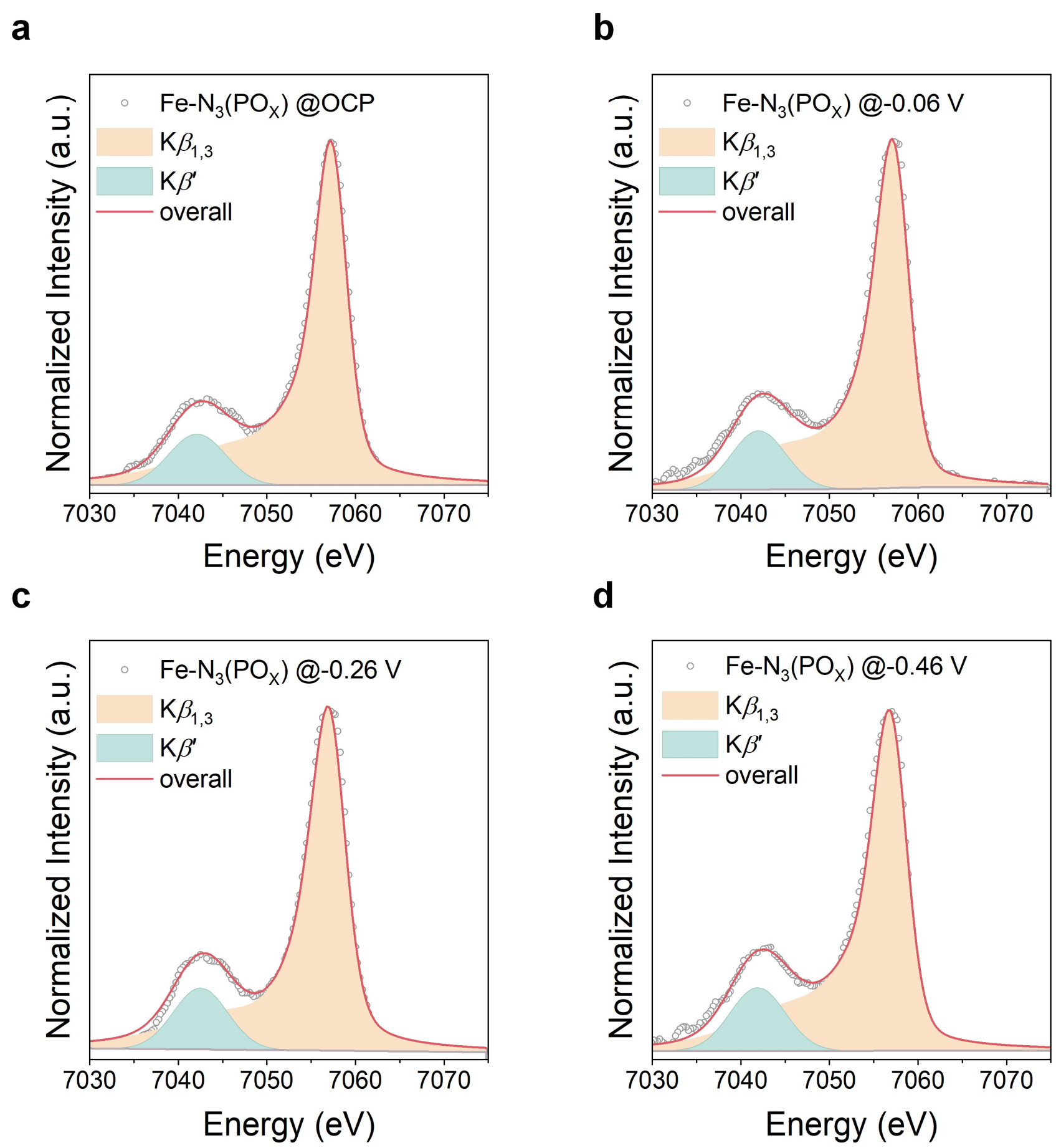

Supplementary Fig. 28. Gaussian fitting of *in-situ* Fe Kβ X-ray emission spectra of Fe-$N_3(PO_X)$ during the NO_3RR process at **a** OCP, **b** -0.06 V, **c** -0.26 V, and **d** -0.46 V. The experimental spectra were fitted using Gaussian functions to deconvolute the Kβ' satellite and K$\beta_{1,3}$ main emission features. All the potentials were not iR corrected. Source data are provided as a Source Data file.

Supplementary Table 9. Fitting results of Fe Kβ emission spectra of Fe-N_3(PO_X) under NO_3RR conditions at different applied potentials. The spectra were fitted using four Gaussian functions to extract the K$\beta_{1,3}$, Kβ', and total Kβ peak areas.

	K$\beta_{1,3}$ area	Kβ' area	Relative Kβ area (%)	Average spin state
OCP	1.42	0.20	12.35	1.96
-0.06 V	1.44	0.22	13.25	2.11
-0.26 V	1.46	0.23	13.61	2.16
-0.46 V	1.47	0.24	14.04	2.23

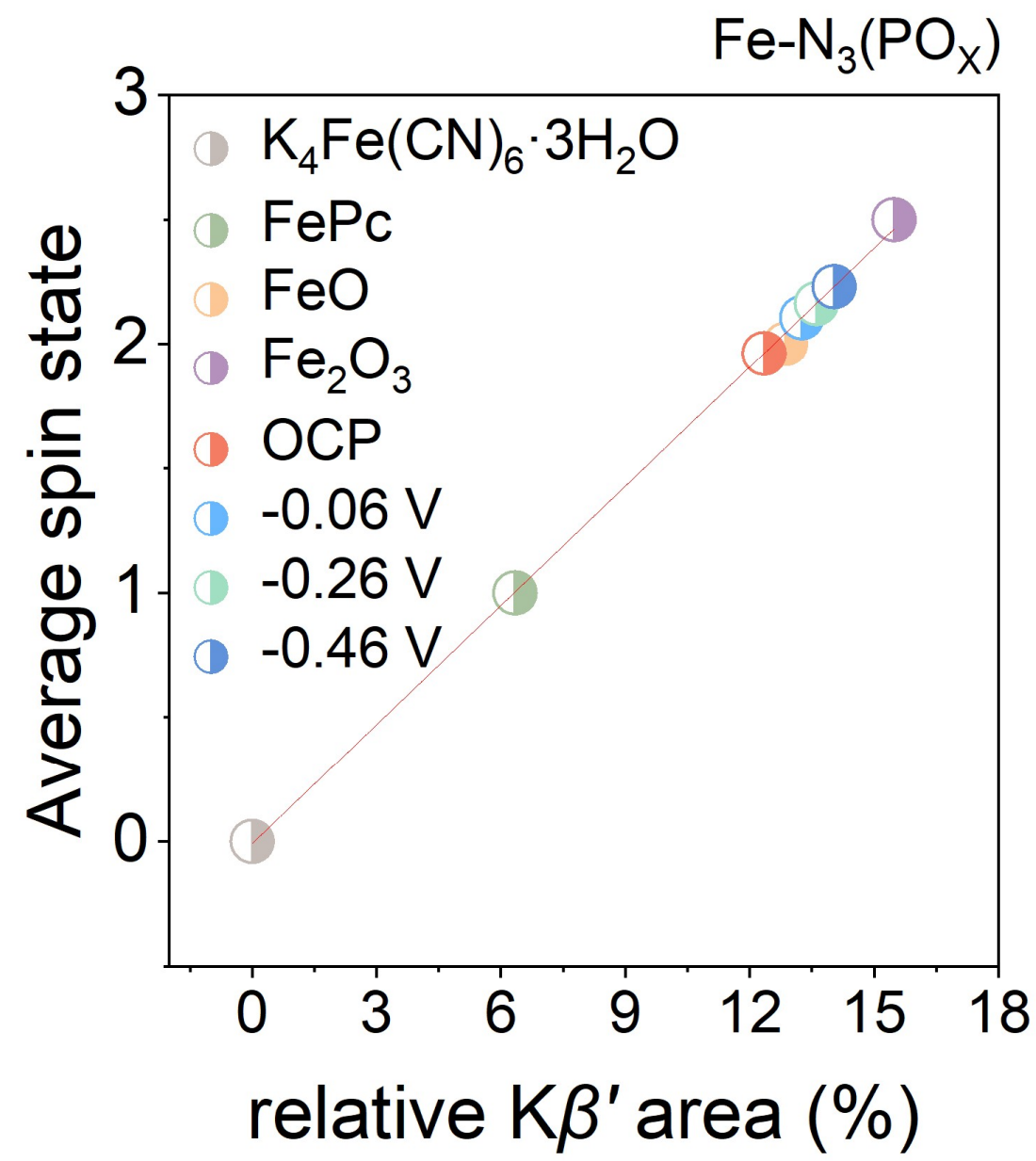

Supplementary Fig. 29. Linear correlation between the relative Kβ' peak area and the average spin state of the reference compounds, showing the positions of Fe-N_3(PO_X) during the NO_3RR conditions for evaluating its spin-state evolution. All the potentials were not iR corrected. Source data are provided as a Source Data file.

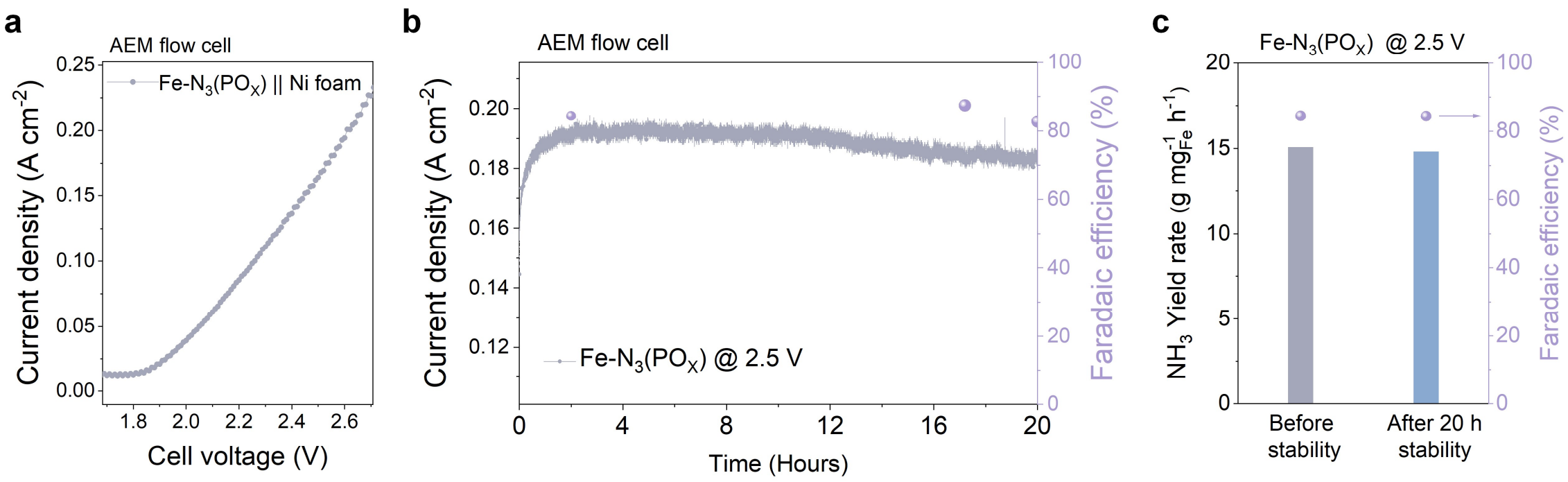

Supplementary Fig. 30. a LSV curve of Fe-N_3(PO_X) measured in an alkaline AEM flow cell. **b** 20 h flow-cell stability test at 2.5 V with corresponding NH_3 Faradaic efficiency. **c** Comparison of NH_3 yield rate and Faradaic efficiency at 2.5 V before and after the 20 h flow-cell stability test. All the voltage were not iR corrected. Source data are provided as a Source Data file.

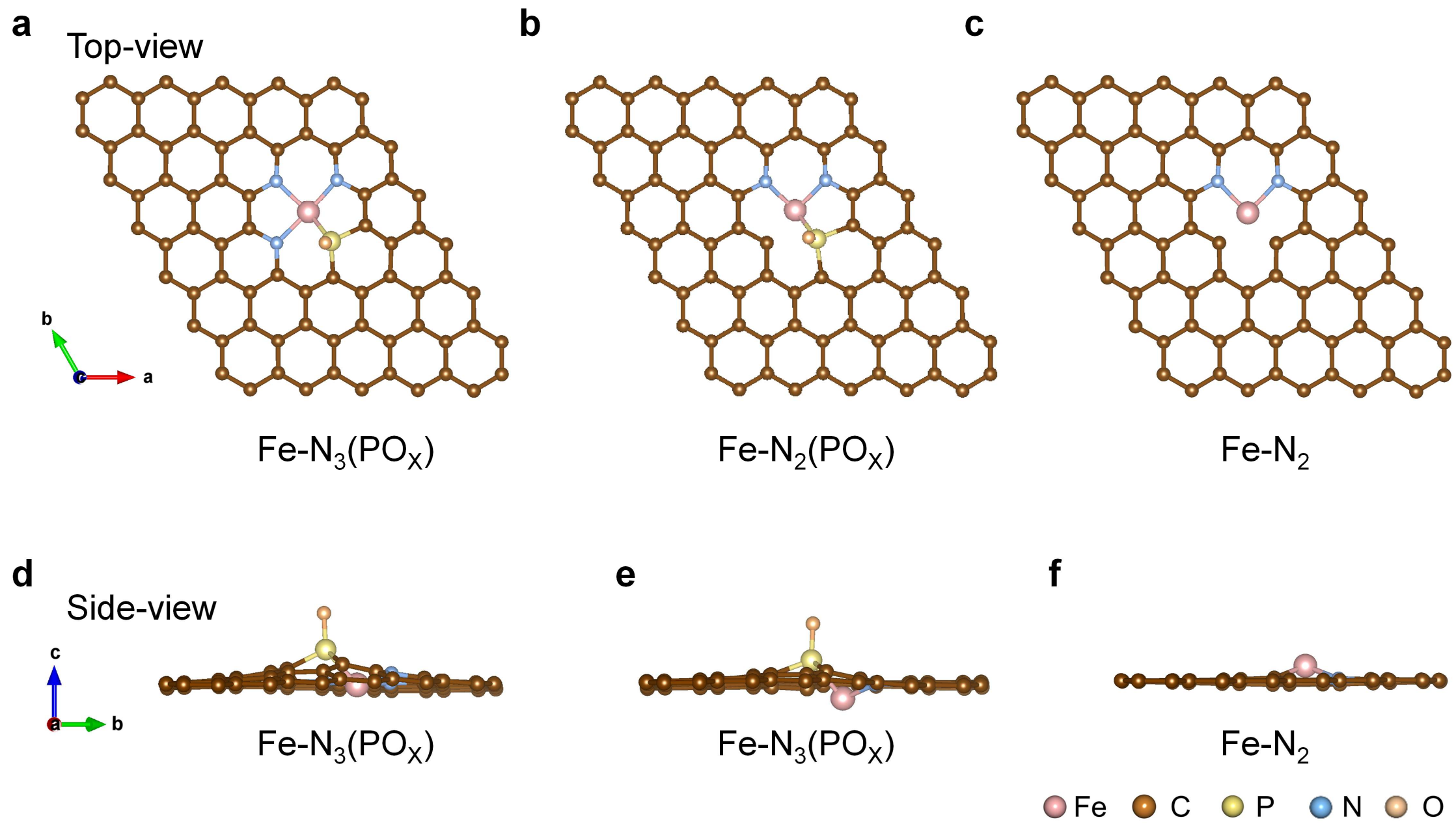

Supplementary Fig. 31. Optimized atomic structures of representative Fe coordination models. **a-c** Top views of Fe-N_3(PO_X), Fe-N_2(PO_X) and Fe-N_2. **d-f** Side views of the corresponding models.

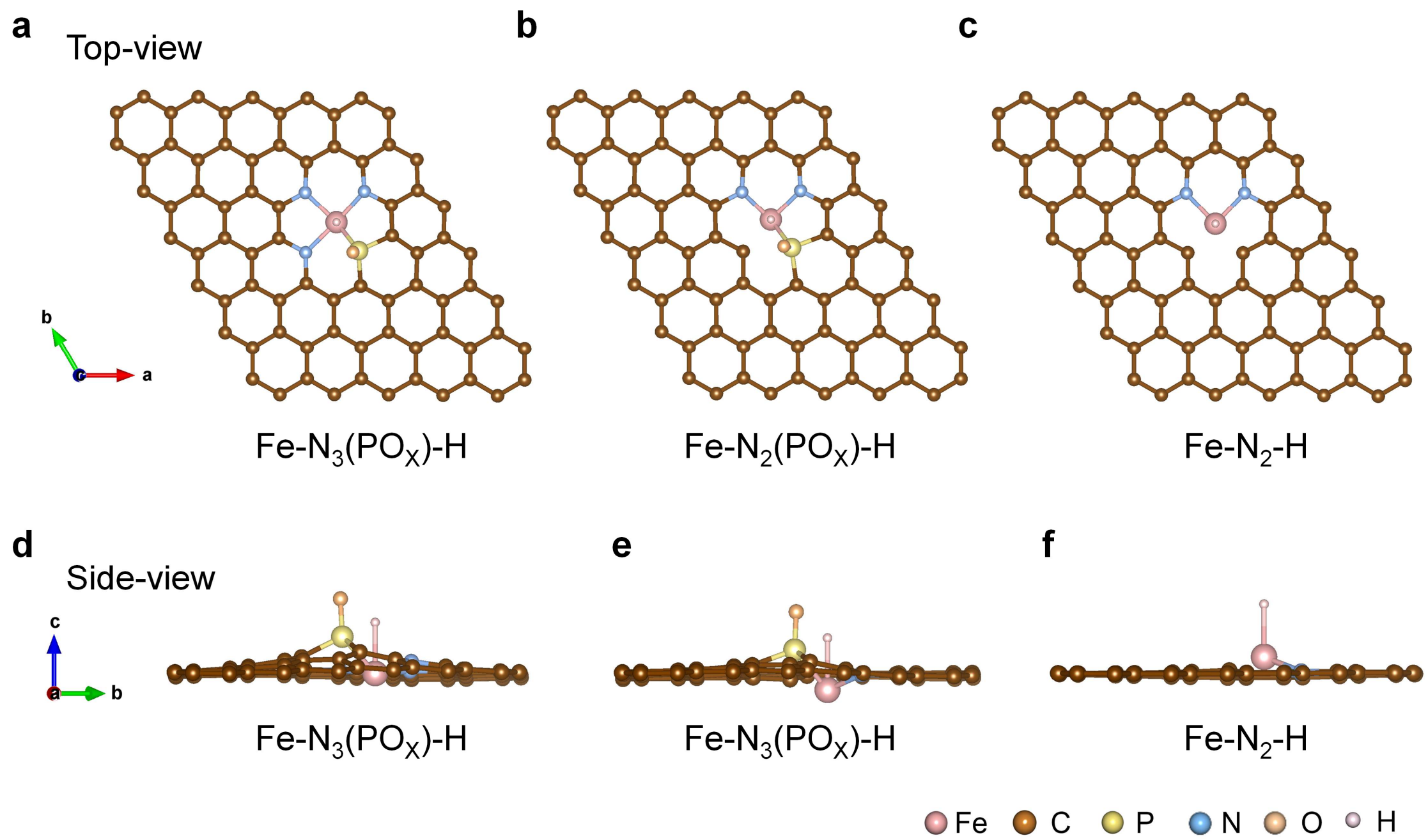

Supplementary Fig. 32. Hydrogen adsorption configurations on three models. **a-c** Top views of $Fe-N_3(PO_X)$, $Fe-N_2(PO_X)$ and $Fe-N_2$. **d-f** Side views of the corresponding models.

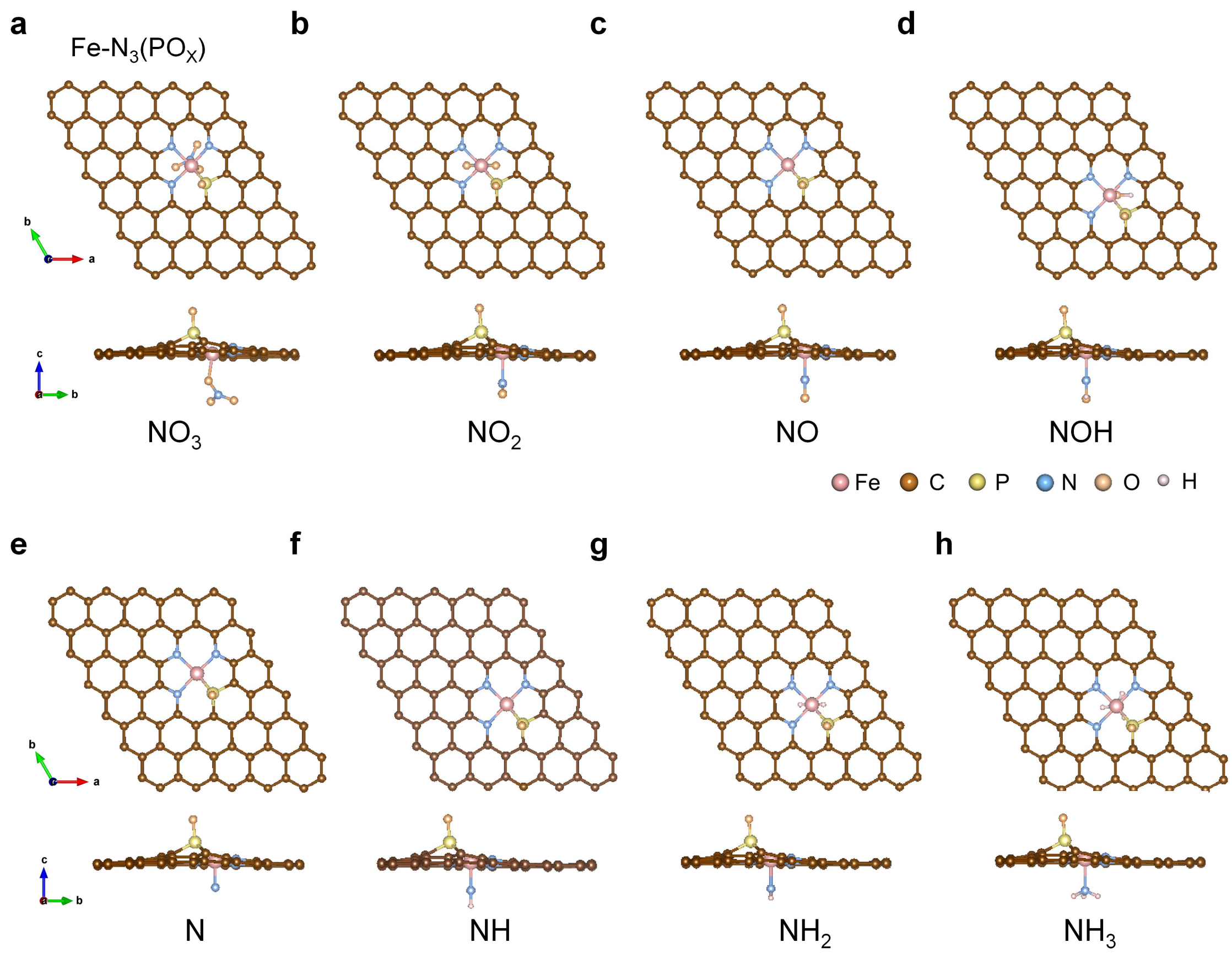

Supplementary Fig. 33. Optimized adsorption configurations of key NO_3RR intermediates on different working-state models. Optimized adsorption structures of **a** NO_3*, **b** NO_2*, **c** NO*, **d** NOH*, **e** N*, **f** NH*, **g** NH_2* and **h** NH_3* on Fe-N_3(PO_X), showing the preferred adsorption configurations during the nitrate reduction pathway.

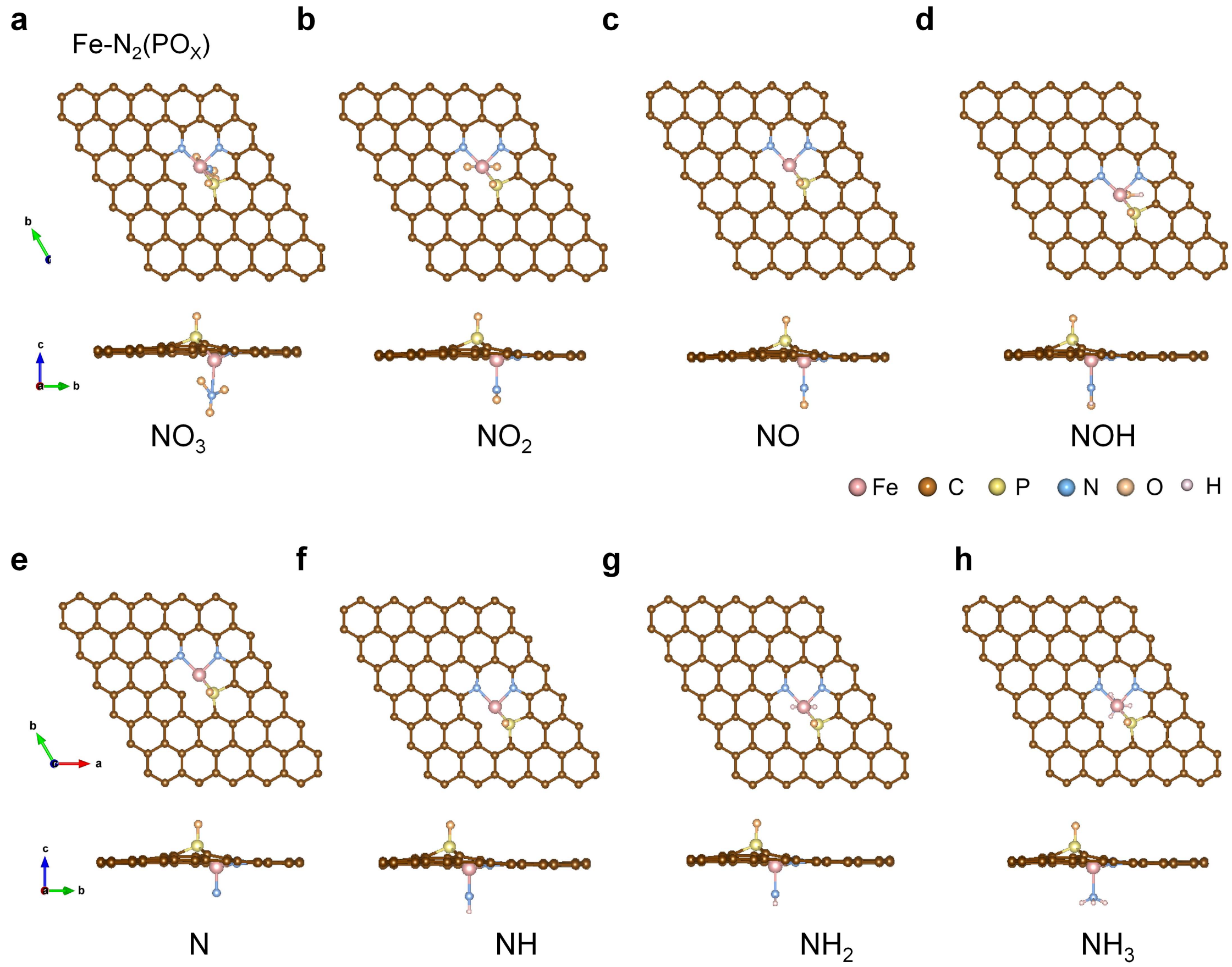

Supplementary Fig. 34. Optimized adsorption configurations of key NO_3RR intermediates on different working-state models. Optimized adsorption structures of **a** NO_3*, **b** NO_2*, **c** NO*, **d** NOH*, **e** N*, **f** NH*, **g** NH_2* and **h** NH_3* on Fe-N_2(PO_X), showing the preferred adsorption configurations during the nitrate reduction pathway.

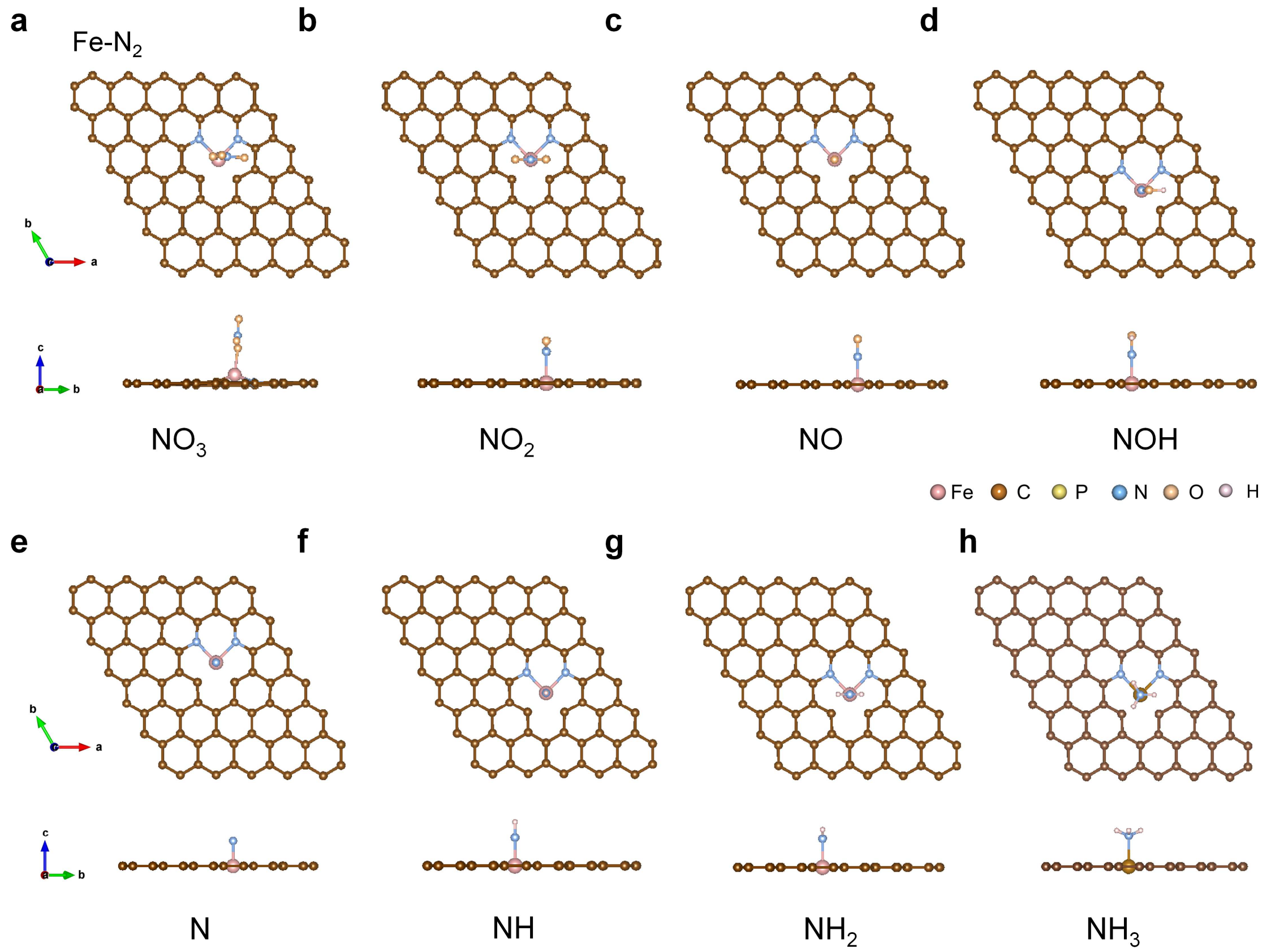

Supplementary Fig. 35. Optimized adsorption configurations of key NO_3RR intermediates on different working-state models. Optimized adsorption structures of Optimized adsorption structures of **a** NO_3*, **b** NO_2*, **c** NO*, **d** NOH*, **e** N*, **f** NH*, **g** NH_2* and **h** NH_3* on Fe-N_2, showing the preferred adsorption configurations during the nitrate reduction pathway.

Supplementary Table 10. Zero-point energy and entropic corrections for key adsorbed intermediates involved in nitrate reduction reaction at 298.15 K.

Species	ZPE (without solvent effect)	-TS
NO_3	0.42	-0.08
$*NO_2$	0.34	-0.07
$*NO$	0.22	-0.05
$*N$	0.06	-0.03
$*NH$	0.36	-0.05
$*NH_2$	0.62	-0.07
$*NH_3$	0.91	-0.10

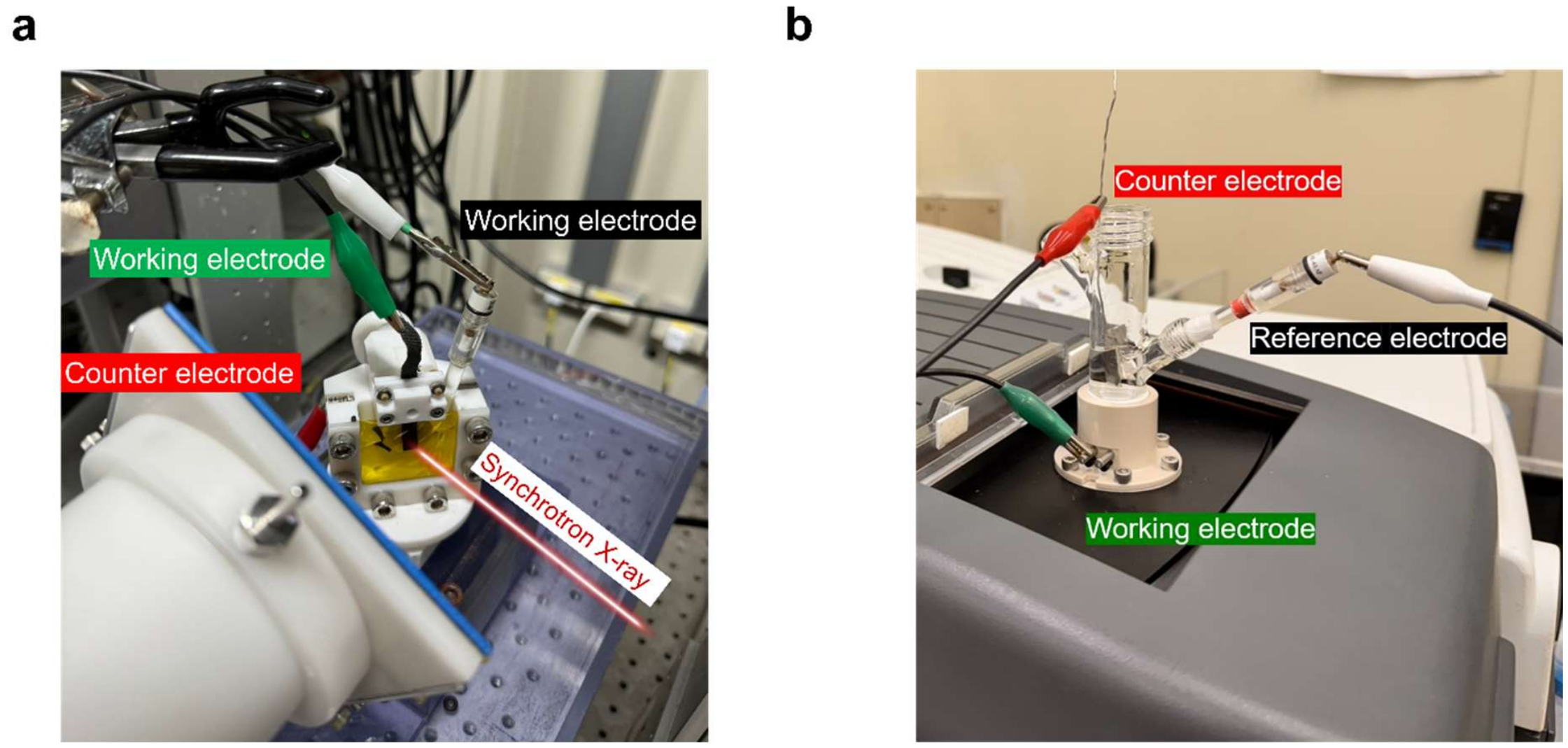

Supplementary Fig. 36. Digital photographs of **a** the *in-situ* XAS cell and **b** the *in-situ* FTIR setup.

Supplementary Table 11. Detailed performance comparison of the Fe-$N_3(PO_X)$ electrode with previously reported NO_3RR single-atom electrocatalysts.

Catalysts	Electrolyte	E (V_{RHE})	FE_{NH3}	NH_3 Yield (g mg_{cat}^{-1} h^{-1})	NH_3 Yield (g mg_{metal}^{-1} h^{-1})	Ref.
Fe-$N_3(PO_X)$	1 M KOH+ 0.1 M KNO_3	-0.36	65.56	0.005108787	2.3762	This work
		-0.46	81.52	0.018070815	8.4050	
		-0.56	89.80	0.047210582	21.9584	
		-0.66	88.32	0.075268619	35.0087	
		-0.76	84.67	0.093280825	43.3864	
2D Fe-SACs	1 M KOH+ 0.1 M KNO_3	-0.2	78.8	0.009315068	0.4658	1
		-0.4	97	0.0222	1.1100	
		-0.6	94	0.032767123	1.6384	
		-0.8	86	0.046136986	2.3068	
		-1	79.6	0.068493151	3.4247	
Fe-N/P-C	0.1 M KOH+ 0.1 M KNO_3	-0.3	85	0.0023	0.2516	2
		-0.4	90.3	0.00583	0.6379	
		-0.5	83	0.008128	0.8893	
		-0.6	80	0.0085	0.9300	
		-0.7	78	0.015	1.6411	
Fe_2O_3/Fe-N-C	1 M KOH+ 0.16 M KNO_3	-0.2	52	0.0068124	0.0162	3
		-0.4	100	0.034062	0.0811	
		-0.6	96	0.0919674	0.2190	
		-0.8	98	0.1464666	0.3487	
		-1	100	0.2350278	0.5596	
Fe_1/Ti	1 M KOH+ 1 M $NaNO_3$	-0.3	100	0.084575946	0.0846	4
		-0.4	95.2	0.266041251	0.2660	
		-0.5	84.6	0.343549332	0.3435	
		-0.6	82	0.554975572	0.5550	
		-0.7	79.2	0.669493719	0.6695	
Fe-TiO_2-M	0.1 M KOH+ 0.1 M KNO_3	-0.3	81.67	0.00422535	0.0621	5
		-0.4	90.56	0.011830986	0.1740	
		-0.5	97.78	0.024577465	0.3614	
		-0.6	90	0.030140845	0.4432	
		-0.7	90.2	0.034859549	0.5126	

Fe-PPy SACs	0.1 M KOH+ 0.1 M KNO_3	-0.2	87	0.000325	0.0137	6
		-0.3	97	0.001583333	0.0665	
		-0.4	90	0.002291667	0.0963	
		-0.5	100	0.005625	0.2363	
		-0.6	98	0.00875	0.3676	
Cl-Fe-NC	1 M KOH+ 0.1 M KNO_3	-0.28	100	0.0034	0.2000	7
		-0.38	99	0.0038	0.2235	
		-0.48	95	0.0072	0.4235	
		-0.58	90	0.0078	0.4588	
		-0.68	75	0.0093967	0.5527	
d-CoCP	1 M KOH+ 0.1 M KNO_3	-0.3	89	0.005895346	0.0732	8
		-0.4	92	0.009825577	0.1221	
		-0.5	97	0.013264529	0.1648	
		-0.6	94	0.016703481	0.2075	
		-0.7	93	0.022107536	0.2746	
Fe/Cu-HNG	1 M KOH+ 0.1 M KNO_3	-0.1	52.5	0.001475696	0.0242	9
		-0.2	80	0.002864583	0.0470	
		-0.3	92.5	0.004166667	0.0683	
		-0.4	82.5	0.005902792	0.0968	
		-0.5	60	0.007118042	0.1167	
RuCu DAs	0.1 M KOH+ 0.1 M KNO_3	-0.2	59.67	0.001355	0.0219	10
		-0.3	76.67	0.0039575	0.0638	
		-0.4	95.33	0.0077075	0.1243	
		-0.5	96.67	0.010825	0.1746	
		-0.6	84.17	0.01375	0.2218	

Reference

1. Li, J., et al. Efficient solar-driven electrocatalytic nitrate-to-ammonia conversion by 2D ultrathin Fe single-atom catalysts. *Journal of Materials Chemistry A* **12,** 26103-26112 (2024).
2. Xu, J., et al. Breaking local charge symmetry of iron single atoms for efficient electrocatalytic nitrate reduction to ammonia. *Angewandte Chemie International Edition* **135,** e202308044 (2023).
3. Murphy, E., et al. Synergizing Fe2O3 nanoparticles on single atom Fe-N-C for nitrate reduction to ammonia at industrial current densities. *Advanced Materials* **36,** 2401133 (2024).
4. Dai, J., et al. Spin polarized Fe1− Ti pairs for highly efficient electroreduction nitrate to ammonia. *Nature Communications* **15,** 88 (2024).
5. Wang, J., et al. Ferromagnetic Fe-TiO(2) spin catalysts for enhanced ammonia electrosynthesis. *Nature Communications* **16,** 1129 (2025).
6. Li, P., et al. A single-site iron catalyst with preoccupied active centers that achieves selective ammonia electrosynthesis from nitrate. *Applied Catalysis B: Environment and Energy* **14,** 3522-3531 (2021).
7. Wan, J., et al. Axial chlorine-induced symmetry-breaking iron single-atom catalyst for electrochemical ammonia synthesis. *ACS Catalysis* **15,** 4507-4518 (2025).
8. Ding, Y., et al. Defect-Enriched Cobalt-Based Coordination Polymers for Selective and Efficient Nitrate Electroreduction to Ammonia. *Advanced Functional Materials* 2422339 (2025).
9. Zhang, S., et al. Fe/Cu diatomic catalysts for electrochemical nitrate reduction to ammonia. *Nature Communications* **14,** 3634 (2023).
10. Liu, K., et al. Tailoring asymmetric RuCu dual-atom electrocatalyst toward ammonia synthesis from nitrate. *Nature Communications* **16,** 2167 (2025).
