# Supplementary material for: Coupled spin-coordination self-adaptation drives efficient nitrate to ammonia conversion
Source: Nat Commun. 2026 Aug 29;17:10334. doi: 10.1038/s41467-026-77163-2 (PMC13623963; doi:10.1038/s41467-026-77163-2)
Supplement: Supplementary file 2 [file 41467_2026_77163_MOESM2_ESM.pdf]

Description of Additional Supplementary Files

File Name: Supplementary Data 1

Description: Atomic coordinates of the optimized computational models used in the DFT calculations, including Fe-N_3(PO_X), Fe-N_2(PO_X) and Fe-N_2.
