# Supplementary material for: Coupled spin-coordination self-adaptation drives efficient nitrate to ammonia conversion
Source: Nat Commun. 2026 Aug 29;17:10334. doi: 10.1038/s41467-026-77163-2 (PMC13623963; doi:10.1038/s41467-026-77163-2)
Supplement: Supplementary file 3 [file 41467_2026_77163_MOESM4_ESM.pdf]

nature portfolio

Peer Review File

Coupled spin-coordination self-adaptation drives efficient nitrate to ammonia conversion

Corresponding Author: Professor Zongping Shao

This file contains all reviewer reports in order by version, followed by all author rebuttals in order by version.

Version 0:

Reviewer comments:

Reviewer #1

(Remarks to the Author)
This paper investigates the impact of spin control on the nitrate reduction. The authors designed a Fe-N3 (POx) single-atom catalyst, enhancing catalytic performance by regulating the Fe spin state and coordination environment. The paper employs multiple in situ characterization techniques to monitor the valence state and spin states of Fe sites during the reaction. The work possesses a certain degree of innovation. However, issues exist in the data analysis and discussion, as well as in the mechanism interpretation. This paper can be accepted after a major revision. Below are my questions.

1. In the introduction, the author emphasizes that “the development of a design strategy that can simultaneously break the Fe-N4 symmetry, drive spontaneous and adaptive spin regulation, and unambiguously identify the true active sites has become a central challenge.” Using this as a benchmark, the authors introduce their subsequent research efforts. However, numerous similar studies were reported, involving either altering the coordination environment of $M-N_4$ by introducing heteroatoms such as P, B, and S, or modifying the symmetry of $M-N_4$ through axial modifications. I recommend that the author refine the innovative points based on the actual characterization contents.

2. The analytical section of XES contains significant issues. In Fig. 1h, the Kβ’ of FeO ref is similar to the porphyrin iron that we have tested. This means there are five single electrons on Fe 3d orbitals. Therefore, the Kβ’ intensity of Fe-N4 or Fe-N3 (POx) proves that more than two unpaired electrons in Fe 3d orbitals. The author should supply XES samples including porphyrin iron (S=5/2) and phthalocyanine iron (S=1) to accurately calculate the number of unpaired electrons in Fe-N3 (POx).

3. Based on the second comment, the schematic of the electronic structure of Fe-N4 and Fe-N3(POx) in Fig. 1i is wrong.

4. The signal for Kβ’ in Figure 4e appears unusual, with the peak area seemingly much larger than that of FeO ref. However, the Fe d orbital can accommodate at most five single electrons. Given this context, the authors should have included the XES of porphyrin iron (S=5/2) and phthalocyanine iron (S=1) in the in situ XES data to ensure the accuracy of the spin state changes.
5. Without theoretical calculations, the author provides numerous schematic diagrams of d-bands and Fermi level positions, such as Fig. 1i and Fig. 3h, which are highly unscientific. I strongly recommend that the author supplement the relevant calculations to substantiate the effect of spin regulation on nitrate reduction. Even more puzzling is that the Fe single site is an atomic-level site with no overlap between the d orbitals of multiple metal atoms, so where do the energy band and d band center come from?

6. For the Fe-N3 (POx) configuration, does POx act as an active site participating in the reaction, such as by promoting proton transfer? or participate in the dissociation of NO_3^-? If these factors cannot be ruled out, the authors cannot claim to have modulated Fe spin to alter catalytic activity.

7. ICP-OES shows Fe contents of 0.314% for Fe-N4 and 0.215% for Fe-N3(POX), and a P content of 7.25% for Fe-N3(POX). Does this mean that a significant amount of phosphorus has been doped into the carbon substrate? Does this affect the catalysis? The author should provide more rigorous controlled experiments.

8. More importantly, since the author tested XES, why are only the Kβ’ data provided, and not the Kβ’’ test results? The Kβ’’

signal is highly effective in distinguishing C/N/O coordination environments, a capability beyond the reach of XAS. The author's research primarily focuses on constructing Fe-N4 and Fe-N3(POX), the corresponding data for Kβ'' and K2,5 should be provided.

Reviewer #2

(Remarks to the Author)
In this work, the authors report an Fe-N3(POX) single-atom catalyst for electrochemical nitrate reduction to ammonia. By breaking the symmetry of conventional Fe-N4 coordination and introducing POX ligands, the catalyst is proposed to dynamically regulate the coordination structure and spin state of Fe active centers under operating conditions. The authors claim that this coupled spin-coordination self-adaptation enhances nitrate activation and hydrogenation, leading to improved ammonia yield rate and Faradaic efficiency. But the experimental evidence and mechanistic discussion are still insufficient to fully support the proposed conclusions. Several important characterizations and analyses are missing or require further clarification. Therefore, the current version of the manuscript is not suitable for publication in Nature Communications and should be rejected.

1. Although the authors claim that the catalyst achieves gram-scale ammonia production, the reported ammonia yield rate is normalized to the mass of Fe single atoms (g mgFe-1 h-1). This normalization may artificially exaggerate the apparent catalytic performance because the Fe loading in single-atom catalysts is typically very low. For a fair comparison with previously reported NO3RR catalysts, the ammonia yield rate should be normalized using more commonly adopted metrics, such as mgNH3 mgcat-1 h-1 or mgNH3 cm-2 h-1. After converting to these standard units, the catalytic performance may not appear as competitive as suggested in the manuscript. Therefore, the authors are encouraged to recalculate the ammonia yield rate using unified units and provide a more rigorous comparison with representative catalysts reported in the literature.
2. The manuscript proposes that the POX-induced asymmetric coordination and spin-state transition of the Fe center play a key role in facilitating nitrate activation and subsequent hydrogenation steps. However, the mechanistic explanation is mainly obtained from qualitative spectroscopic interpretation. To further substantiate this claim, theoretical calculations should be provided. In particular, density functional theory calculations should be performed to evaluate the adsorption energies of key intermediate sites. Moreover, the free-energy profiles of the nitrate reduction pathway should be constructed to identify the potential-determining step and clarify how the POX coordination and spin-state modulation influence the reaction energetics. Such theoretical insights may significantly strengthen the mechanistic understanding and provide stronger support for the proposed catalytic model.
3. Although the catalyst exhibits promising Faradaic efficiency and ammonia yield rate under laboratory conditions, the practical applicability of the system remains unclear. In particular, the current densities reported in the manuscript are relatively low compared with those required for industrial nitrate-to-ammonia conversion. It is recommended that the authors evaluate the catalytic performance at higher current densities to better demonstrate the potential for practical application.
4. In addition, the stability test presented in the manuscript is relatively limited. Considering that the proposed mechanism involves dynamic coordination reconstruction of the Fe active sites, longer-term electrolysis should be conducted to verify the structural robustness and catalytic durability of the catalyst under continuous operation. Post-reaction characterizations would also be helpful to confirm whether the atomic dispersion and coordination environment remain intact after prolonged electrolysis.

Reviewer #3

(Remarks to the Author)
The authors have designed an adaptive coordination transformation coupled with dynamic regulation of the Fe spin state, enabling the catalyst to exhibit excellent activity and selectivity for electrochemical nitrate-to-ammonia conversion. This study provides important insights into how the coordination dynamics and spin-state modulation of single-atom catalytic sites can be leveraged to enhance the activity and selectivity of NO3RR toward ammonia production. In particular, the combination of in-situ XAS, XES, and FTIR offers strong support for the proposed reaction mechanism. Overall, the manuscript is well organized and presents novel findings, although several minor issues still need to be clarified to further improve the quality of the work. This work is worthy of publication in Nature Communications after minor revision. My specific comments are as follows.
1. The authors may discuss the role of active hydrogen species in either the Introduction or the Discussion section, with appropriate citation to relevant literature.
2. When the term "asymmetric adsorption" is first introduced in the main text, the authors should provide a brief definition and clarify its potential advantages.
3. The manuscript suggests that stabilizing the asymmetric adsorption mode is beneficial for the reaction. To further validate this experimentally observed trend, the authors are encouraged to provide theoretical calculations (or cite relevant literature) comparing the hydrogenation energy barriers for symmetric versus asymmetric nitrate adsorption configurations. Such analysis would help confirm whether the asymmetric mode is indeed kinetically more favorable.
4. Likewise, for Lines 307-309, where the authors state that "the asymmetric mode is more readily hydrogenated by a nearby adsorbed H," appropriate literature citations should be included to support this claim.
5. The applied potentials should be clearly indicated in the in-situ FTIR data to facilitate direct comparison with other in situ measurements. Currently, the potential intervals are only provided in the table and manuscript text. Please clearly label the corresponding potentials in both the figure and the Methods section.

Version 1:

Reviewer comments:

Reviewer #1

(Remarks to the Author)
The author has made complete and detailed revisions to the comments, and I suggest that the manuscript be accepted directly

Reviewer #2

(Remarks to the Author)
This manuscript has been revised in response to our previous comments. However, some comments are still not replied directly and the central arguments were not salient, despite the heavy workload, including
i) No solid proof can prove the proposed reaction pathway for the NO3RR. Particularly, the insignificant In-situ ATR-FTIR signals cannot be assigned to the intermediate species, which is an equivocal inference. Although the authors reposition the manuscript as a mechanistic study, the mechanistic understanding provided remains insufficient. In NO3RR, the identification of key reaction intermediates and dominant reaction pathways represents the most fundamental basis for mechanistic interpretation. However, these issues are still not clearly resolved in the present work. While operando characterizations of catalyst reconstruction are provided, the manuscript mainly focuses on structural evolution itself, without convincingly establishing how such reconstruction directly governs the catalytic pathway or activity. As a result, the proposed structure–activity relationship and mechanistic conclusions remain largely speculative and are not yet adequately supported by direct experimental or theoretical evidence.
ii) The authors attribute the absence of theoretical calculations to the dynamic coordination reconstruction of Fe single-atom sites under NO3RR conditions. However, this argument is not sufficiently convincing and instead exposes a more fundamental issue in the current mechanistic interpretation. If the catalyst structure is continuously evolving under operating conditions, this directly raises concerns regarding the structural stability and identity of the true active sites. More importantly, the existence of reconstruction cannot be used as a justification for avoiding theoretical analysis. On the contrary, if the reconstructed configuration is proposed to be the actual active state, then representative working-state models should be established based on the operando characterization results, followed by corresponding theoretical calculations to clarify the structure–activity relationship and reaction pathway.
iii) Repositioning the manuscript as a “mechanistically informative model catalyst platform” does not fully address the original concern. For a study targeting a high-impact journal such as Nature Communications, application-oriented evaluations are not merely optional additions, but are increasingly expected to demonstrate the broader significance and practical relevance of the proposed catalyst system. Simply acknowledging that the current density is insufficient for practical operation does not eliminate the need for meaningful application-level assessment. More importantly, the manuscript now attempts to shift its emphasis entirely toward mechanistic understanding. However, as discussed above, the mechanistic insights themselves remain insufficiently established, since key reaction intermediates, dominant pathways, and realistic active-site configurations have not been conclusively identified. Therefore, the work currently lacks both the practical demonstration expected for application-driven NO3RR studies and the mechanistic rigor required for a fundamentally oriented study.
In conclusion, I still can't recommend the publication of this manuscript on Nature Communications.

Reviewer #3

(Remarks to the Author)
In this updated version, I can see the authors have properly addressed the reaction mechanism concerns including the active sites, the innovations were re-clarified. Overall, I am satisfied with the revision and would like to suggest acceptance.

Version 2:

Reviewer comments:

Reviewer #2

(Remarks to the Author)
All comments are addressed.

Reviewer #4

(Remarks to the Author)
As a new reviewer for this revised manuscript, I checked the response letter, revised manuscript, and the newly added computational results. This is a excellent work and thus I recommend acceptance in its current form.

Open Access This Peer Review File is licensed under a Creative Commons Attribution 4.0 International License, which permits use, sharing, adaptation, distribution and reproduction in any medium or format, as long as you give appropriate credit to the original author(s) and the source, provide a link to the Creative Commons license, and indicate if changes were made.
In cases where reviewers are anonymous, credit should be given to 'Anonymous Referee' and the source.
The images or other third party material in this Peer Review File are included in the article's Creative Commons license, unless indicated otherwise in a credit line to the material. If material is not included in the article's Creative Commons license and your intended use is not permitted by statutory regulation or exceeds the permitted use, you will need to obtain permission directly from the copyright holder.
To view a copy of this license, visit https://creativecommons.org/licenses/by/4.0/

Response to Decision Letter
Manuscript ID:

Thank you for reviewers' serious and constructive comments on our manuscript. According to their suggestions, the manuscript has been revised as a letter to editor. The revisions and replies we have made are as follows:

Reviewer #1:
This paper investigates the impact of spin control on the nitrate reduction. The authors designed a Fe-N_3 (PO_X) single-atom catalyst, enhancing catalytic performance by regulating the Fe spin state and coordination environment. The paper employs multiple *in-situ* characterization techniques to monitor the valence state and spin states of Fe sites during the reaction. The work possesses a certain degree of innovation. However, issues exist in the data analysis and discussion, as well as in the mechanism interpretation. This paper can be accepted after a major revision. Below are my questions.

We sincerely thank the reviewer for the careful evaluation and constructive comments. We appreciate the reviewer's recognition of the novelty of this work and fully agree that several parts of the original manuscript required more rigorous analysis and more cautious interpretation.

1. In the introduction, the author emphasizes that "the development of a design strategy that can simultaneously break the Fe-N_4 symmetry, drive spontaneous and adaptive spin regulation, and unambiguously identify the true active sites has become a central challenge." Using this as a benchmark, the authors introduce their subsequent research efforts. However, numerous similar studies were reported, involving either altering the coordination environment of M-N_4 by introducing heteroatoms such as P, B, and S, or modifying the symmetry of M-N_4 through axial modifications. I recommend that the author refine the innovative points based on the actual characterization contents.

Response: We thank the reviewer for this important suggestion.

We agree that simply introducing heteroatoms or breaking the symmetry of M-N_4 single-atom sites has been widely reported, and that these strategies alone should not be overemphasized as the main novelty of this work. Accordingly, in the revised manuscript, we have refined the innovation of this study based on the actual in-situ characterization results. In the revised Introduction, we now clarify that although heteroatom doping and axial coordination have been widely used to tune the electronic structures of M-N_4 single-atom sites, most related mechanisms are still discussed based on relatively static structural models. For a complex reaction such as NO_3RR, which involves multiple nitrate-derived intermediates and coupled electron/proton transfer steps, the active site may undergo continuous coordination and electronic evolution under operating potentials. Therefore, beyond simply constructing an asymmetric Fe coordination environment, it is more important to directly identify how the local coordination structure, spin state, and nitrate-intermediate adsorption behavior evolve under working conditions.

Based on this consideration, the novelty of this work has been redefined as revealing the dynamic coupling between coordination reconstruction, spin-state evolution, and nitrate-reduction behavior in Fe-N_3(PO_X) single-atom sites. Specifically, *in-situ* XAS captures the potential-induced coordination reconstruction of the Fe site, *in-situ* XES tracks the accompanying spin-state modulation, and *in-situ* ATR-FTIR correlates these

structural/electronic changes with nitrate-intermediate adsorption and conversion. These results establish a direct link between local structural/electronic evolution and NO_3RR catalytic behavior, rather than merely demonstrating heteroatom-induced symmetry breaking. The corresponding description has been revised in the Introduction (Lines 87-90).

2. The analytical section of XES contains significant issues. In Fig. 1h, the K*β'* of FeO ref is similar to the porphyrin iron that we have tested. This means there are five single electrons on Fe 3*d* orbitals. Therefore, the K*β'* intensity of Fe-N_4 or Fe-$N_3(PO_X)$ proves that more than two unpaired electrons in Fe 3*d* orbitals. The author should supply XES samples including porphyrin iron (S=5/2) and phthalocyanine iron (S=1) to accurately calculate the number of unpaired electrons in Fe-$N_3(PO_X)$.

Response: We sincerely thank the reviewer for this important and professional comment.

We agree that the Kβ' feature in XES should not be interpreted only by direct visual comparison, and that appropriate reference compounds and quantitative fitting are necessary for a more reliable evaluation of the Fe spin state. In the revised manuscript, we have added a more rigorous XES fitting analysis. Specifically, the Kβ' emission spectra were fitted by deconvoluting the main K$\beta_{1,3}$ emission line and the Kβ' satellite feature, as shown in Supplementary Fig. S24a-e. Reference compounds, including FePc, FeO, and Fe_2O_3, were used for comparison and calibration. We also compared the relative Kβ' peak areas of these reference compounds with reported literature values, which show comparable trends and support the validity of our fitting procedure. The extracted relative Kβ' peak areas are summarized in Supplementary Table 7. Based on these values, we further constructed a linear correlation between the relative Kβ' peak area and the average spin state of the reference compounds, as shown in Supplementary Fig. 25. Fe-N_4 and Fe-$N_3(PO_X)$ were then positioned on this fitted calibration curve for spin-state evaluation. The fitting results indicate that Fe-$N_3(PO_X)$ indeed possesses a slightly higher apparent average spin-state character than Fe-N_4. Specifically, the estimated average spin-state value increases from 1.51 for Fe-N_4 to 1.82 for Fe-$N_3(PO_X)$. We emphasize that this value should be regarded as an experimentally derived average spin-state evaluation, rather than an exact assignment of an integer spin state or a direct count of unpaired Fe 3*d* electrons. Therefore, the revised XES analysis supports our conclusion that the Fe-$N_3(PO_X)$ configuration promotes spin-state modulation of the Fe center. The corresponding fitting results, calibration curve, and discussion have been added to the revised manuscript (Lines 177-186) and Supporting Information Fig 24a-e, 25 and Table7.

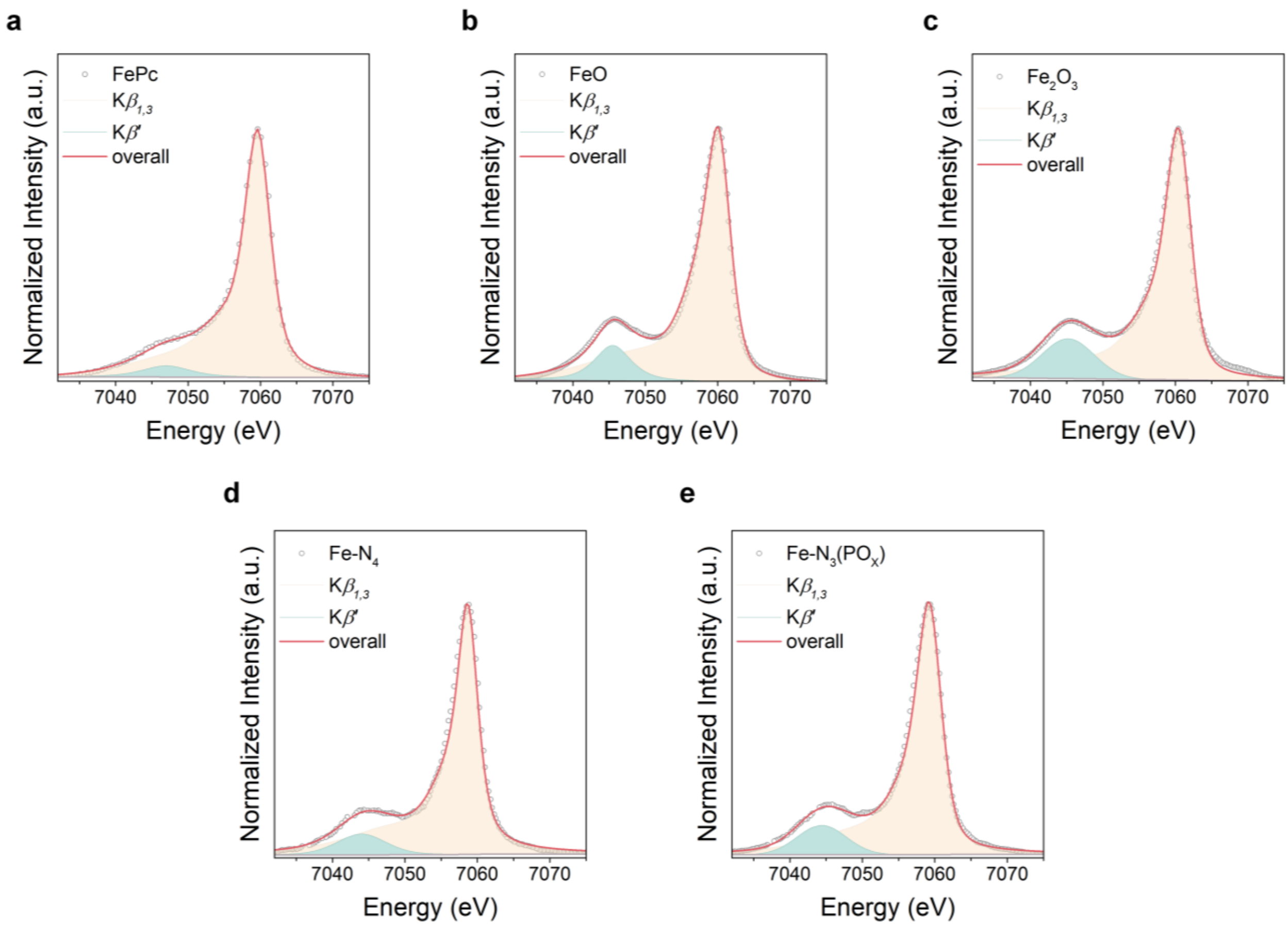

Supplementary Fig. 24. Gaussian fitting of Fe Kβ X-ray emission spectra of **a** iron phthalocyanine (FePc) reference, **b** FeO reference, **c** Fe_2O_3 reference, **d** Fe-N_4 and **e** Fe-$N_3(PO_X)$. The experimental spectra were fitted using Gaussian functions to deconvolute the Kβ' satellite and K$\beta_{1,3}$ main emission features.

	K$\beta_{1,3}$ area	Kβ' area	Relative Kβ area (%)	Average spin state
FePc	5.34	0.51	6.33	1
FeO	5.75	1.21	12.77	2
Fe_2O_3	6.11	1.48	15.48	2.5
Fe-N_4	5.80	0.82	9.30	1.51
Fe-$N_3(PO_X)$	5.90	1.02	11.23	1.82

Supplementary Table 7. Fitting results of Fe Kβ emission spectra of the reference compounds, Fe-N_4 and Fe-$N_3(PO_X)$. The spectra were fitted using four Gaussian functions to extract the K$\beta_{1,3}$, Kβ', and total Kβ peak areas.

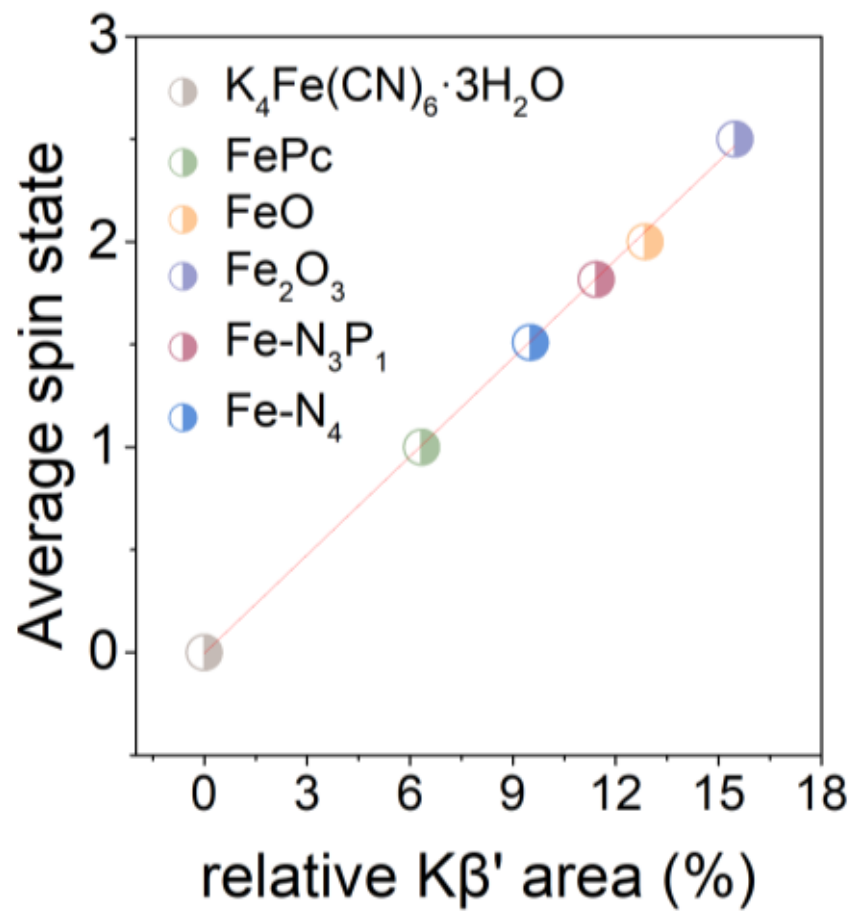

Supplementary Fig. 25. Linear correlation between the relative Kβ' peak area and the average spin state of the reference compounds with Fe-N_4 and Fe-$N_3(PO_X)$ positioned on the fitted calibration curve for spin-state evaluation..

3. Based on the second comment, the schematic of the electronic structure of Fe-N_4 and Fe-$N_3(PO_X)$ in Fig. 1i is wrong.

Response: We sincerely thank the reviewer for this important comment.

We agree that the original schematic illustration of the electronic structure in Fig. 1i was oversimplified and may have implied a definite orbital filling configuration that was not fully supported by the experimental data. In the revised manuscript, we have therefore removed the detailed orbital-filling schematic from Fig. 1i and Fig. 4f. Although our XES fitting and calibration analysis indicate that the estimated average spin-state value increases 1.51 for Fe-N_4 to 1.82 for Fe-$N_3(PO_X)$, this value should be regarded as an experimentally derived average spin-state evaluation rather than a direct assignment of a pure integer spin state or a specific electron occupation. Because the Fe single-atom site is strongly affected by its local coordination environment, covalency, and possible mixed spin-state character, assigning an exact electron-filling diagram without additional theoretical or multiplet calculations may lead to overinterpretation. Therefore, in the revised Fig. 1i, we no longer present a specific electron-filling model. Instead, we describe the electronic structure more cautiously in terms of experimentally supported spin-state modulation, namely that Fe-$N_3(PO_X)$ exhibits a relatively higher spin-state character than Fe-N_4. The corresponding discussion has also been revised to avoid overstatement and to better align with the XES fitting results. In addition, we retained the original description but reorganized the figure layout for clarity. The P K-edge XANES data have been moved to Supplementary Fig. 7b, while the XES results are now presented in Fig. 1g.

4. The signal for Kβ' in Figure 4e appears unusual, with the peak area seemingly much larger than that of FeO ref. However, the Fe *d* orbital can accommodate at most five single electrons. Given this context, the authors should have included the XES of porphyrin iron (S=5/2) and phthalocyanine iron (S=1) in the *in-situ* XES data to ensure the accuracy of the spin state changes.

Response: We sincerely thank the reviewer for this important comment.

To address this issue, we have added a quantitative Gaussian fitting analysis of the *in-situ* Fe Kβ XES spectra in the revised manuscript. As shown in Supplementary Fig. 28, the *in-situ* Fe Kβ XES spectra of Fe-$N_3(PO_X)$ collected during the NO_3RR process were fitted by deconvoluting the Kβ' satellite feature and the K$\beta_{1,3}$ main emission line. The extracted relative Kβ' peak areas are summarized in Supplementary Table 9.

For spin-state calibration, reference compounds including Fe_2O_3 (S=5/2) were used as high-spin Fe references, together with other Fe reference compounds for comparison and calibration. We also compared the relative Kβ′ peak areas of the reference compounds with reported literature values to ensure a fair and reasonable calibration range [*Nature Sustainability* 8.7 (2025): 793-805, *Angewandte Chemie* 133.21 (2021): 11813-11818.].

Based on these reference compounds, we established a linear correlation between the relative Kβ′ peak area and the average spin-state value, as shown in Supplementary Fig. 29. The results show that the apparent average spin-state character of Fe-N_3(PO_X) gradually increases from OCP to the applied potentials during NO_3RR, indicating a progressive enhancement of the higher-spin character at the Fe center under reaction conditions. We emphasize that this analysis is used to evaluate the relative spin-state evolution rather than to assign an exact number of unpaired electrons. Therefore, the *in-situ* XES results support a potential-dependent increase in the spin-state character of Fe-N_3(PO_X) during NO_3RR. The corresponding fitting analysis, calibration curve, and discussion have been added to the revised manuscript. (Lines 358-367).

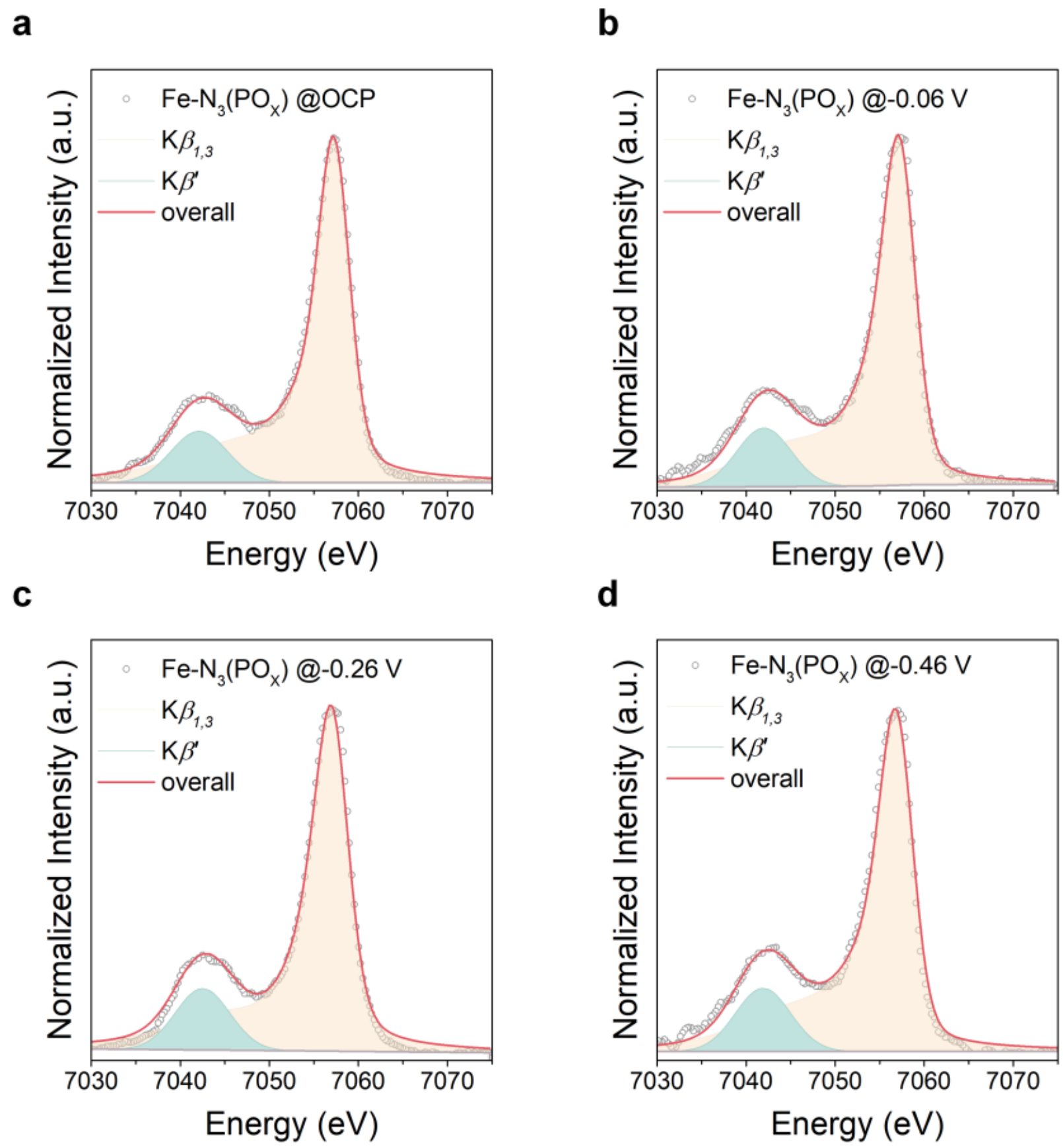

Supplementary Fig. 28. Gaussian fitting of *in-situ* Fe Kβ X-ray emission spectra of Fe-N_3(PO_X) during the NO_3RR process at **a** OCP, **b** -0.06 V, **c** -0.26 V, and **d** -0.46 V. The experimental spectra were fitted using Gaussian functions to deconvolute the Kβ′ satellite and $K\beta_{1,3}$ main emission features.

	$K\beta_{1,3}$ area	$K\beta'$ area	Relative $K\beta$ area (%)	Average spin state
OCP	1.42	0.20	12.35	1.96
-0.06 V	1.44	0.22	13.25	2.11
-0.26 V	1.46	0.23	13.61	2.16
-0.46 V	1.47	0.24	14.04	2.23

Supplementary Table 9. Fitting results of Fe $K\beta$ emission spectra of Fe-$N_3(PO_X)$ under NO_3RR conditions at different applied potentials. The spectra were fitted using four Gaussian functions to extract the $K\beta_{1,3}$, $K\beta'$, and total $K\beta$ peak areas.

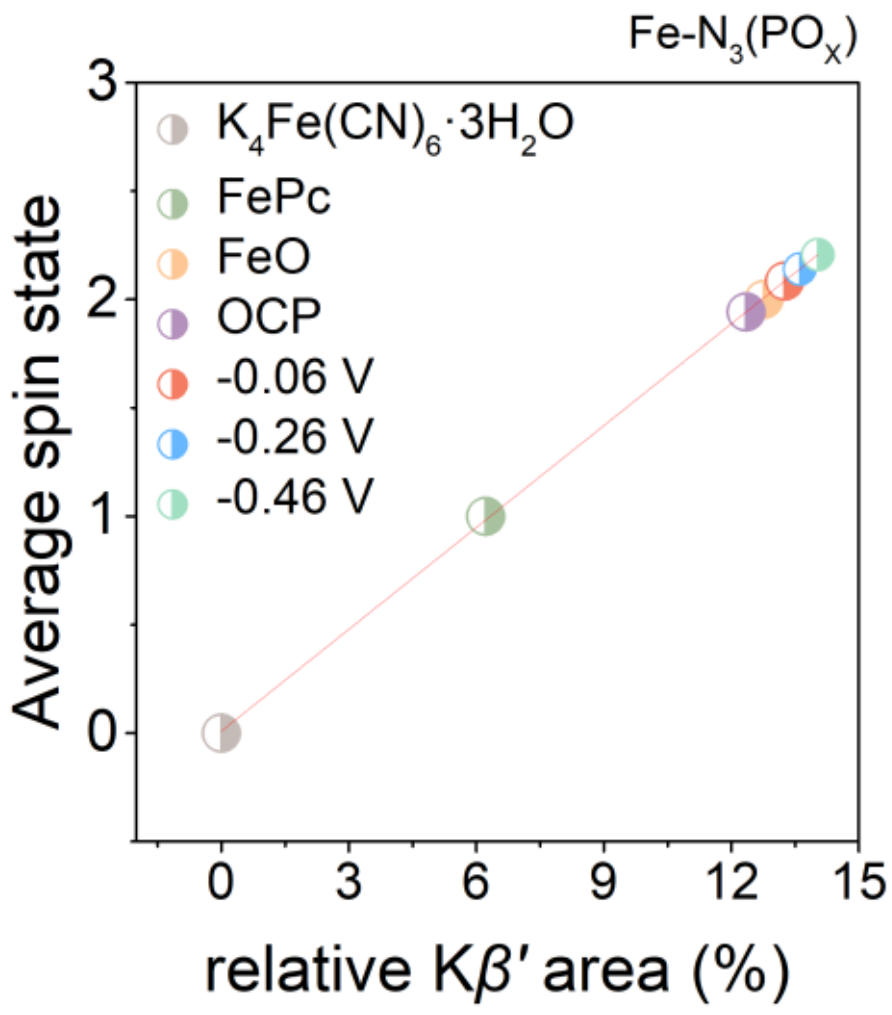

Supplementary Fig. 29. Linear correlation between the relative $K\beta'$ peak area and the average spin state of the reference compounds, showing the positions of Fe-$N_3(PO_X)$ during the NO_3RR conditions for evaluating its spin-state evolution.

5. Without theoretical calculations, the author provides numerous schematic diagrams of *d*-bands and Fermi level positions, such as Fig. 1i and Fig. 3h, which are highly unscientific. I strongly recommend that the author supplement the relevant calculations to substantiate the effect of spin regulation on nitrate reduction. Even more puzzling is that the Fe single site is an atomic-level site with no overlap between the d orbitals of multiple metal atoms, so where do the energy band and *d* band center come from?

Response: We sincerely thank the reviewer for this important comment.

We fully agree with the reviewer that the original *d*-band and Fermi-level schematics were oversimplified and not sufficiently rigorous for an isolated Fe single-site catalyst, particularly in the absence of supporting theoretical calculations. Unlike extended metal surfaces, an Fe single-atom site should be described in terms of localized Fe 3*d* states that are strongly influenced by the local coordination environment, covalency, and spin-state character, rather than by a broad metallic *d* band. Therefore, in the revised manuscript, we have removed the unsubstantiated *d*-band/Fermi-level schematics in Fig. 1i and Fig. 3h and revised the related discussion accordingly. We no longer use *d*-band-center language to describe the Fe single site. Instead, the role of spin regulation in NO_3RR is discussed more cautiously on the basis of experimentally supported changes in the local electronic/spin-state character of the Fe center. We also avoid assigning quantitative

adsorption-energy barriers, potential-determining steps, or Fermi-level shifts without theoretical support. The revised discussion now presents the proposed mechanism as an *in-situ* spectroscopy supported correlation among coordination reconstruction, spin-state modulation, nitrate-related intermediate evolution, and catalytic behavior. We appreciate the reviewer's criticism, which helped us remove inappropriate band-based interpretations for an isolated atomic site. The relevant descriptions have been removed or revised throughout the manuscript.

6. For the Fe-N_3 (PO_X) configuration, does PO_X act as an active site participating in the reaction, such as by promoting proton transfer? or participate in the dissociation of NO_3^-? If these factors cannot be ruled out, the authors cannot claim to have modulated Fe spin to alter catalytic activity.

Response: We thank the reviewer for raising this critical point.

We agree that the possible minor contribution of PO_X to the local reaction microenvironment, including proton-transfer assistance or nitrate activation, should be considered carefully. To avoid overstatement, we have revised the manuscript and no longer describe Fe spin modulation as the sole origin of the enhanced activity. Instead, we now present PO_X as an electronic and structural modulator that induces an asymmetric coordination environment around the Fe center and promotes potential-dependent coordination reconstruction and spin-state evolution during NO_3RR. Importantly, the newly added PO_X-doped carbon control experiment shows only very low current density and negligible NH_3 production under identical NO_3RR conditions, indicating that PO_X-containing carbon without Fe cannot act as an efficient independent active phase. In addition, the *in-situ* structural results show that the Fe-PO_X coordination is largely weakened/broken at -0.46 V, suggesting that PO_X does not remain as a stable, independent primary active site throughout the reaction. On this basis, we conclude that the enhanced catalytic performance mainly originates from the PO_X-induced modulation and reconstruction of the Fe single-atom center, while acknowledging that PO_X may also contribute to the local coordination/electronic microenvironment. The corresponding discussion has been revised to reflect this more cautious interpretation (Line 282-286).

7. ICP-OES shows Fe contents of 0.314% for Fe-N_4 and 0.215% for Fe-N_3(PO_X), and a P content of 7.25% for Fe-N_3(PO_X). Does this mean that a significant amount of phosphorus has been doped into the carbon substrate? Does this affect the catalysis? The author should provide more rigorous controlled experiments.

Response: We thank the reviewer for this important comment.

To exclude the possible contribution of PO_X-doped carbon itself to the NO_3RR activity, we added a PO_X-doped carbon (PO_X-C) blank control sample in the revised manuscript and tested it under the same NO_3RR conditions. The results show that the current density of this control sample is much lower than those of Fe-N_4 and Fe-N_3(PO_X), indicating that PO_X-C alone cannot generate significant NO_3RR electrocatalytic current. Furthermore, we performed the indophenol blue assay for the PO_X-C blank control to determine the amount of NH_3 produced after electrolysis at -0.46 V vs. RHE. The UV-vis absorption signal of this control sample is very weak, with almost no detectable NH_3 production. This result further confirms that PO_X-C itself makes a negligible contribution to the NO_3RR activity.

Based on these control experiments, we conclude that the enhanced NO_3RR performance of Fe-N_3(PO_X) mainly originates from the modulation of the local coordination environment and electronic structure of the Fe center. Specifically, the introduction of PO_X modifies the asymmetric coordination structure around the Fe single-atom site and further regulates the electronic structure and spin state of the Fe center, thereby promoting

the NO_3^- reduction reaction. We have added the corresponding experimental results and discussion for the PO_X -C blank control in the revised manuscript, including its NO_3RR LSV curve and indophenol assay results, to clearly demonstrate the limited contribution of PO_X-C itself to the catalytic activity and to further support that the high activity of Fe-N_3(PO_X) mainly arises from the PO_X-modulated Fe single-atom active centers. We have added the discussion in the revised manuscript (Lines 188-193) and Fig 2a.

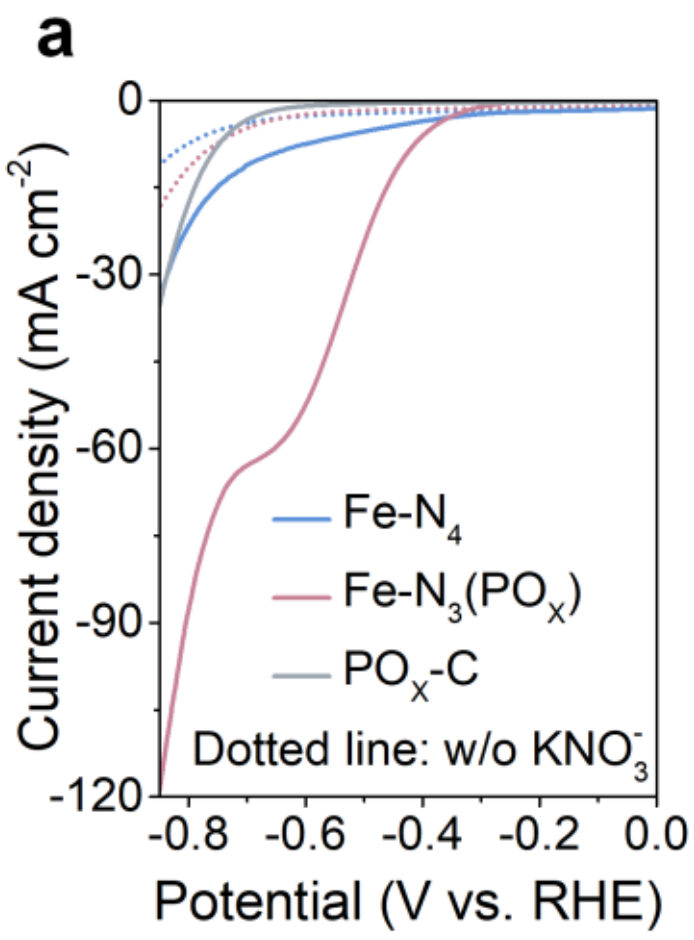

Fig. 2a Linear sweep voltammetry curves of Fe-N_4, Fe-N_3(PO_X) and POx-carbon control in 1.0 M KOH without and with 0.1 M KNO_3 mixed electrolyte (with *iR* correction).

8. More importantly, since the author tested XES, why are only the K*β'* data provided, and not the K*β''* test results? The K*β''* signal is highly effective in distinguishing C/N/O coordination environments, a capability beyond the reach of XAS. The author's research primarily focuses on constructing Fe-N_4 and Fe-N_3(PO_X), the corresponding data for K*β''* and $K_{2,5}$ should be provided.

Response: We sincerely thank the reviewer for this professional and constructive suggestion.

We agree that the $K\beta''$ and $K\beta_{2,5}$ valence-to-core XES features can provide additional information on the local coordination environment of light elements. In particular, they have potential advantages in distinguishing C/N/O coordination atoms and can serve as an important complement to XAS-based structural analysis. In general, the intensity of the valence-to-core XES region is approximately two orders of magnitude weaker than that of the $K\beta$ mainline, namely about 100 times weaker [*Physical Chemistry Chemical Physics* 26.29 (2024): 19960-19975]. Therefore, reliable measurement of these weak features is intrinsically technically challenging. This limitation becomes particularly significant for single-atom catalysts. In the present study, the Fe contents of Fe-N_4 and Fe-N_3(PO_X) are relatively low, and the number of Fe single-atom sites contributing to the XES signal is therefore limited. As a result, the weak $K\beta''$ and $K\beta_{2,5}$ features could not be reliably resolved with sufficient signal-to-noise ratio.

Therefore, to avoid overinterpretation of low-quality signals, we did not include the $K\beta''$ and $K\beta_{2,5}$ region in the mechanistic discussion. Instead, we focused on the $K\beta'$ and $K\beta_{1,3}$ features, which show much higher signal quality and are widely used for spin-state analysis, to evaluate the average spin-state character of the Fe sites. The coordination structures of Fe-N_4 and Fe-N_3(PO_X) were confirmed by Fe K-edge XANES/EXAFS fitting, wavelet-transform EXAFS analysis, and complementary elemental and structural characterizations, including P 2*p* XPS and P K-edge XAS. We have revised the manuscript to clarify this limitation and avoid overinterpretation of the unresolved valence-to-core features.

Reviewer #2:

In this work, the authors report an Fe-$N_3(PO_X)$ single-atom catalyst for electrochemical nitrate reduction to ammonia. By breaking the symmetry of conventional Fe-N_4 coordination and introducing PO_X ligands, the catalyst is proposed to dynamically regulate the coordination structure and spin state of Fe active centers under operating conditions. The authors claim that this coupled spin-coordination self-adaptation enhances nitrate activation and hydrogenation, leading to improved ammonia yield rate and Faradaic efficiency. But the experimental evidence and mechanistic discussion are still insufficient to fully support the proposed conclusions. Several important characterizations and analyses are missing or require further clarification. Therefore, the current version of the manuscript is not suitable for publication in Nature Communications and should be rejected.

We sincerely thank the reviewer for the careful reading of our manuscript and for the constructive comments. The reviewer's concerns regarding the rigor of the mechanistic interpretation and the presentation of the catalytic evaluation have been highly valuable in improving the quality and rigor of this work.

1. Although the authors claim that the catalyst achieves gram-scale ammonia production, the reported ammonia yield rate is normalized to the mass of Fe single atoms (g mg_{Fe}^{-1} h^{-1}). This normalization may artificially exaggerate the apparent catalytic performance because the Fe loading in single-atom catalysts is typically very low. For a fair comparison with previously reported NO_3RR catalysts, the ammonia yield rate should be normalized using more commonly adopted metrics, such as mg_{NH_3} mg_{cat}^{-1} h^{-1} or mg_{NH_3} cm^{-2} h^{-1}. After converting to these standard units, the catalytic performance may not appear as competitive as suggested in the manuscript. Therefore, the authors are encouraged to recalculate the ammonia yield rate using unified units and provide a more rigorous comparison with representative catalysts reported in the literature.

Response: We thank the reviewer for this important comment.

We agree that reporting the ammonia yield rate only on the basis of Fe content is not sufficient for broad comparison across different NO_3RR catalysts, especially when the metal loading and catalyst architecture differ substantially. To avoid potentially misleading comparisons, we have removed the direct comparison with non-single-atom systems from the main text. In the revised manuscript, the NH_3 yield rate normalized to Fe content is retained only for comparison among low-loading Fe single-atom catalysts and related systems. In addition, the NH_3 yield rate normalized to the total catalyst mass (mg_{NH_3} mg_{cat}^{-1} h^{-1}) is provided in the Supporting Information for broader comparison with catalysts having higher metal loadings or different architectures. The corresponding results and comparisons have been updated in the revised manuscript (Lines 253-255 and 260-268, Fig. 2g, and Supplementary Fig. 18a, b).

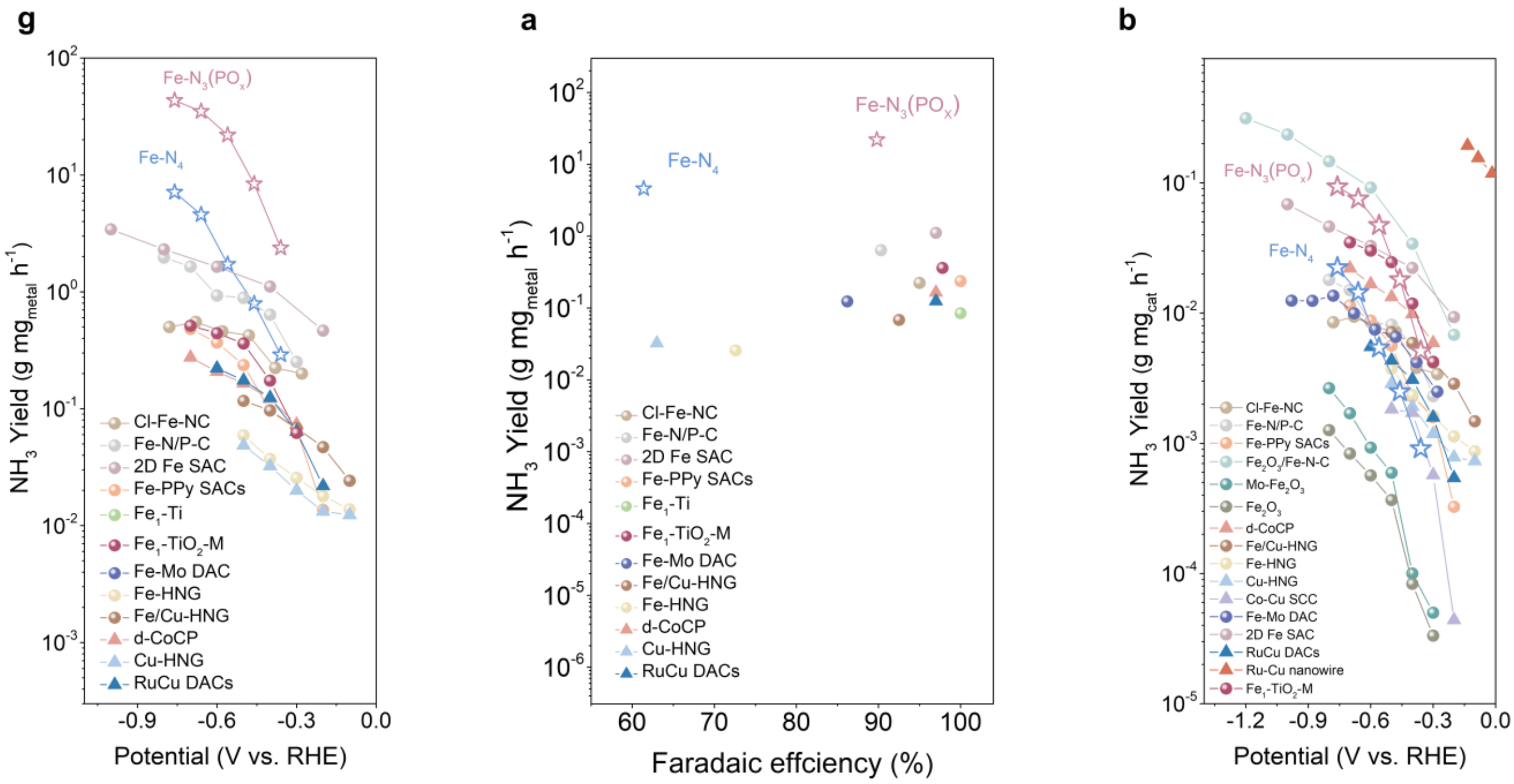

Fig. 2g Performance comparison of the Fe-$N_3(PO_X)$ electrode with previously reported NO_3RR single-atom electrocatalysts **and Supplementary Fig. 18a** Comparison of NH_3 yield rate and Faradaic efficiency for NO_3RR over Fe-$N_3(PO_X)$, Fe-N_4, and recently reported single-atom catalysts. **b** Performance comparison of the Fe-$N_3(PO_X)$ electrode with representative NO_3RR electrocatalysts based on the ammonia yield rate normalized to the total catalyst mass.

2. The manuscript proposes that the PO_X-induced asymmetric coordination and spin-state transition of the Fe center play a key role in facilitating nitrate activation and subsequent hydrogenation steps. However, the mechanistic explanation is mainly obtained from qualitative spectroscopic interpretation. To further substantiate this claim, theoretical calculations should be provided. In particular, density functional theory calculations should be performed to evaluate the adsorption energies of key intermediate sites. Moreover, the free-energy profiles of the nitrate reduction pathway should be constructed to identify the potential-determining step and clarify how the PO_X coordination and spin-state modulation influence the reaction energetics. Such theoretical insights may significantly strengthen the mechanistic understanding and provide stronger support for the proposed catalytic model.

Response: We thank the reviewer for this valuable suggestion.

We fully agree that density functional theory calculations can provide useful insight into intermediate adsorption energetics and reaction pathways in electrocatalytic NO_3RR, especially when the catalytically relevant active site can be reasonably represented by a well-defined and structurally stable model. However, in the present Fe-$N_3(PO_X)$ system, our *in-situ* spectroscopic results indicate that the Fe single-atom site does not remain as a static coordination motif under NO_3RR conditions. Instead, the Fe center undergoes potential-dependent coordination reconstruction, accompanied by changes in its local electronic/spin-state character. Therefore, a free-energy profile constructed from a single fixed structural model, such as the initial Fe-$N_3(PO_X)$ configuration, may not faithfully represent the actual working state of the catalyst. Several possible working-state structures may coexist or evolve sequentially under applied potentials, and each may exhibit different adsorption configurations, spin states, solvation environments, and proton/electron-transfer energetics. Under such circumstances, a simplified static DFT model could provide an apparently quantitative pathway but may lead to mechanistic overinterpretation. To address the reviewer's concern while avoiding unsupported

theoretical claims, we have substantially revised the manuscript. First, we removed the original d-band/Fermi-level schematics and no longer discuss the isolated Fe single-atom site using metallic band descriptors. Second, we toned down the mechanistic language throughout the manuscript and no longer assign a quantitative adsorption-energy barrier or potential-determining step. Third, we now define the proposed mechanism as an experimentally supported correlation among potential-induced coordination reconstruction, spin-state modulation, nitrate-intermediate adsorption behavior, and NO_3RR catalytic performance, based on the combined *in-situ* XAS, *in-situ* XES, *in-situ* ATR-FTIR, and electrochemical results. Specifically, in-situ XAS reveals the potential-dependent reconstruction of the local Fe coordination environment, *in-situ* XES tracks the accompanying change in the average spin-state character of the Fe center, and *in-situ* ATR-FTIR shows the evolution of nitrate-related adsorption/intermediate features within the same reaction-relevant potential range. These results collectively support that the PO_X-induced asymmetric coordination environment promotes a dynamically adaptive Fe site during NO_3RR. We therefore present the proposed mechanism as an experimentally grounded mechanistic interpretation rather than a DFT-derived quantitative reaction pathway. The relevant descriptions, figure captions, and unsupported statements related to d-band centers, Fermi-level positions, adsorption-energy barriers, and potential-determining steps have been revised accordingly.

3. Although the catalyst exhibits promising Faradaic efficiency and ammonia yield rate under laboratory conditions, the practical applicability of the system remains unclear. In particular, the current densities reported in the manuscript are relatively low compared with those required for industrial nitrate-to-ammonia conversion. It is recommended that the authors evaluate the catalytic performance at higher current densities to better demonstrate the potential for practical application.

Response: We thank the reviewer for the careful reading of our work and for this important comment.

We agree that the current densities reported in this manuscript remain below those required for practical or industrial nitrate-to-ammonia conversion. The reviewer's comment is very helpful for clarifying the scope of the present study. Because the Fe loading in this single-atom catalyst system is relatively low, the achievable current output is inherently limited, and high-current-density operation was not the primary objective of this work. Accordingly, in the revised manuscript, we have removed statements implying direct "practical application" and limited the discussion to the improved catalytic activity demonstrated under laboratory conditions. We have also redefined the focus of this work as revealing local coordination reconstruction, spin-state evolution, and their correlation with catalytic behavior for asymmetric Fe single-atom sites during NO3RR, rather than demonstrating a catalyst system already optimized for industrially relevant operation. Therefore, the present study is more appropriately viewed as a mechanistically informative model catalyst platform rather than a practical high-current-density system. The relevant statements have been revised in Lines 259-263.

4. In addition, the stability test presented in the manuscript is relatively limited. Considering that the proposed mechanism involves dynamic coordination reconstruction of the Fe active sites, longer-term electrolysis should be conducted to verify the structural robustness and catalytic durability of the catalyst under continuous operation. Post-reaction characterizations would also be helpful to confirm whether the atomic dispersion and coordination environment remain intact after prolonged electrolysis.

Response: We thank the reviewer for the careful reading of our work and for this important suggestion.

To address this concern, we have added a 24 h continuous electrolysis test at -0.46 V to further evaluate

the catalytic durability under prolonged operation (Supplementary Fig. 26). The current density remained stable during the 24 h electrolysis without obvious decay, demonstrating the good operational stability of the catalyst. After 24 h of continuous electrolysis, the catalyst still achieved a high Faradaic efficiency of 84.37% and an NH_3 yield rate of 8.06 g mg_{Fe}^{-1} h^{-1}, which are close to the values obtained at -0.46 V in the initial performance test. These results indicate that Fe-$N_3(PO_X)$ can maintain stable NO_3RR activity and selectivity under long-term operation. In addition, we performed XAS characterization before and after the stability test to examine whether the Fe sites maintain their atomic dispersion and local coordination environment after extended electrolysis. The XAS results before and after electrolysis indicate that the Fe species remain atomically dispersed. More importantly, the coordination number obtained from the post-reaction EXAFS fitting is 2.1, closely matching the value of 2.2 derived from the *in-situ* EXAFS fitting at -0.46 V. This result further supports that the catalyst retains a local coordination environment similar to the working-state active structure after prolonged operation (Supplementary Fig. 27 and Table 8). These results collectively demonstrate the structural robustness and catalytic durability of Fe-$N_3(PO_X)$ under long-term NO_3RR operation. The corresponding data and discussion have been added in the revised manuscript (Lines 239-255 and Supplementary Figs. 26 and 27).

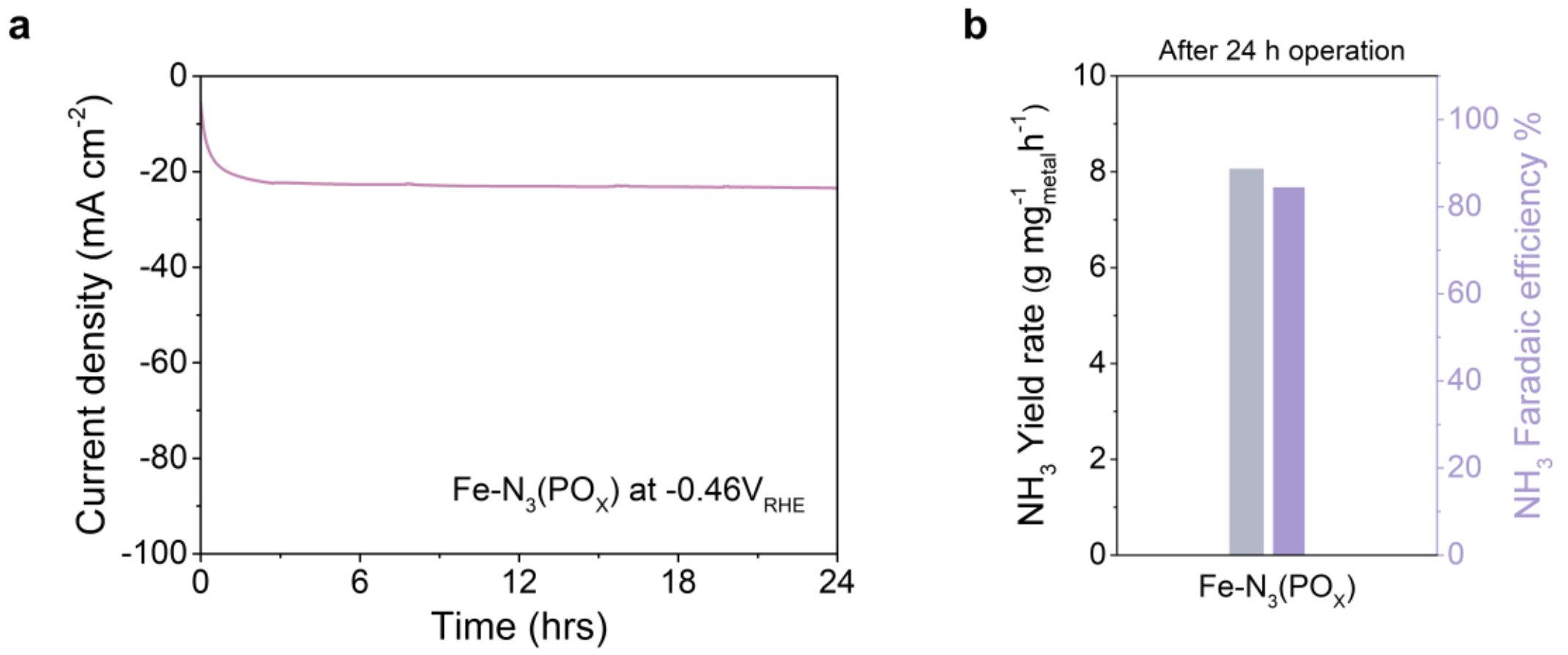

Supplementary Fig. 26. a chronoamperometry stability test for 24 hours at -0.46 V vs. RHE **b** corresponding NH_3 yield rate and Faradaic efficiency after 24 h of stability test.

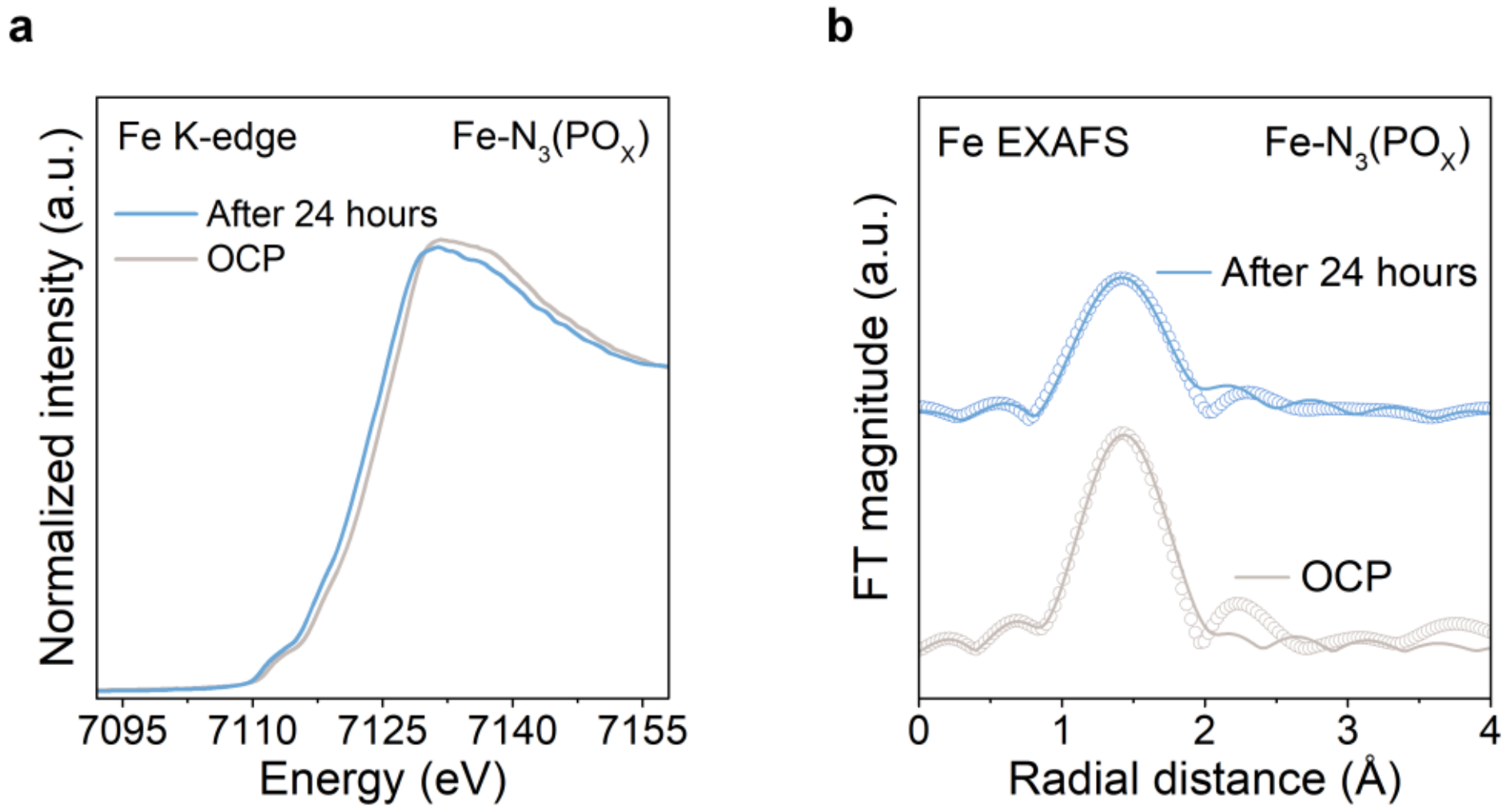

Supplementary Fig. 27. a XANES and **b** EXAFS spectra of Fe-$N_3(PO_X)$ collected before and after 24 h stability testing at -0.46 V vs. RHE.

Sample	Shell	CN	R (Å)	$\sigma^2(10^{-3}Å^{-2})$	ΔE_0 (eV)	*R*-factor
OCP	Fe-N	3.0 ±0.5	1.81 ±0.03	7.0±0.1	11.36±1.5	0.003
	Fe-P(O_X)	1.0 ±0.5	2.18 ±0.07			
After 24 hours @-0.46 V	Fe-N	2.1±0.1	1.84 ±0.06	3.9 ±0.1	10.37±5.9	0.011

Supplementary Table 8. Summary of structural parameters extracted from Fe K-edge EXAFS fitting of Fe-$N_3(PO_X)$ before and after the NO_3RR test.

Reviewer #3:

The authors have designed an adaptive coordination transformation coupled with dynamic regulation of the Fe spin state, enabling the catalyst to exhibit excellent activity and selectivity for electrochemical nitrate-to-ammonia conversion. This study provides important insights into how the coordination dynamics and spin-state modulation of single-atom catalytic sites can be leveraged to enhance the activity and selectivity of NO_3RR toward ammonia production. In particular, the combination of *in-situ* XAS, XES, and FTIR offers strong support for the proposed reaction mechanism. Overall, the manuscript is well organized and presents novel findings, although several minor issues still need to be clarified to further improve the quality of the work. This work is worthy of publication in Nature Communications after minor revision. My specific comments are as follows.

We sincerely thank the reviewer for the positive comments and recommendation for publication, which have been highly valuable in improving this work.

1. The authors may discuss the role of active hydrogen species in either the Introduction or the Discussion section, with appropriate citation to relevant literature.

Response: Many thanks for the reviewer's important suggestion and the helpful literature pointers.

Recent studies have demonstrated that active hydrogen species play an important role in NO_3RR, particularly in the stepwise hydrogenation of nitrate-derived intermediates toward NH_3. For example, previous literature has shown that the dynamic equilibrium between active hydrogen generation and its timely consumption by nitrogen-containing intermediates is critical for achieving high ammonia yield and Faradaic efficiency. In the revised manuscript, we have added a discussion of this issue in the Introduction. For Fe SACs, active hydrogen regulation is particularly important because the isolated Fe center needs to balance nitrate/NOx adsorption, subsequent hydrogenation steps, and the competing HER. In addition, relevant Fe SAC studies have shown that hydrogenation-related steps, such as NO* reduction to HNO* and HNO* reduction to N*, can serve as key potential-limiting steps during NO_3RR. Compared with Cu-based catalysts, which are often effective in nitrate adsorption and N-O bond activation, and Co-based catalysts, which have been associated with promoting the H* pathway, Fe SACs still require careful balancing of nitrate/NOx adsorption, active hydrogen utilization, and HER suppression.

Therefore, improving NO_3RR activity and selectivity does not simply rely on increasing *H coverage, but requires effective generation, transfer, and utilization of active hydrogen while suppressing HER competition. In this work, the asymmetric $Fe\text{-}N_3(PO_X)$ coordination environment modulates the local electronic structure and spin state of the Fe center, and further undergoes dynamic coordination reconstruction during NO_3RR. Such structural and spin-state evolution may help improve the balance between nitrate-derived intermediate activation and subsequent hydrogenation steps, thereby enhancing NO_3RR activity and NH_3 selectivity. The corresponding discussion and literature citations have been added to the revised manuscript (Lines 71-78).

2. When the term "asymmetric adsorption" is first introduced in the main text, the authors should provide a brief definition and clarify its potential advantages.

Response: We thank the reviewer for this helpful suggestion.

We agree that the term "asymmetric adsorption" should be clearly defined when it is first introduced. In the revised manuscript, we have added a brief explanation of asymmetric adsorption and clarified its potential advantage for NO_3RR, together with appropriate references.

Specifically, NO_3^- has a planar structure with D_{3h} symmetry, in which the excess negative charge is delocalized over the entire ion and the three N-O bonds are equivalent under ideal conditions. In this work, "asymmetric adsorption" refers to a locally symmetry-broken adsorption configuration induced by the catalyst surface or local coordination environment. When interfacial interactions or hydrogen bonding preferentially involve one N-O group, the local symmetry of NO_3^- can be broken, accompanied by transient elongation of the involved N-O bond and charge redistribution within the nitrate ion. Such an asymmetric ν_{as}(N-O) configuration may make one oxygen terminus more reactive and facilitate subsequent protonation/hydrogenation steps, thereby accelerating nitrate conversion and improving NO_3RR kinetics.
This explanation has been incorporated at the first mention of "asymmetric adsorption" in the revised manuscript, with appropriate references (Lines 325-331).

3. The manuscript suggests that stabilizing the asymmetric adsorption mode is beneficial for the reaction. To further validate this experimentally observed trend, the authors are encouraged to provide theoretical calculations (or cite relevant literature) comparing the hydrogenation energy barriers for symmetric versus asymmetric nitrate adsorption configurations. Such analysis would help confirm whether the asymmetric mode is indeed kinetically more favorable.

Response: We thank the reviewer for this insightful suggestion.

We agree that comparing the hydrogenation energy barriers of symmetric and asymmetric nitrate adsorption configurations would be valuable for theoretically confirming the kinetic advantage of the asymmetric adsorption mode. However, in the present Fe-N_3(PO_X) system, the Fe active site undergoes dynamic coordination reconstruction during NO_3RR, as revealed by the *in-situ* XAS results. Therefore, a theoretical model based on a single static adsorption configuration may not adequately represent the real working state of the catalyst and could lead to an oversimplified mechanistic interpretation. Instead of adding a potentially incomplete static calculation, we have strengthened the discussion by combining literature precedent with the trends observed in our *in-situ* ATR-FTIR and electrochemical results.
Specifically, NO_3^- has a planar structure with D_{3h} symmetry, in which the excess negative charge is delocalized over the entire ion and the three N-O bonds are equivalent under ideal conditions. The so-called asymmetric adsorption can be attributed to local symmetry breaking induced by the catalyst surface or local coordination environment. When hydrogen bonding or interfacial interactions preferentially involve one N-O group, this local asymmetry can be accompanied by transient elongation of the involved N-O bond and charge redistribution within the nitrate ion [J. Phys. Chem. A 114, 1255–1269 (2010); J. Chem. Phys. 93, 3379–3388 (1990)]. Such an asymmetric adsorption configuration may make one oxygen terminus more reactive and facilitate subsequent protonation/hydrogenation or deoxygenation-related steps, thereby improving NO_3RR kinetics.

Moreover, our *in-situ* ATR-FTIR and electrochemical results provide experimental support for this mechanistic interpretation. As shown in Fig 4c and Fig. 2d, Fe-N_3(PO_X) exhibits a higher ν_{as}/ν_s ratio than Fe-

N_4 under operating potentials, indicating a larger fraction of asymmetric nitrate-related consumption. Meanwhile, Fe-N_3(PO_X) also shows enhanced NH_3 partial current density, NH_3 yield rate, and Faradaic efficiency within the corresponding potential range. This correlation between the increased asymmetric nitrate signature and improved NO_3RR performance supports the catalytic relevance of asymmetric adsorption in our system.

Accordingly, we now describe the asymmetric adsorption mode more cautiously as an experimentally observed and literature-supported configuration that may facilitate subsequent hydrogenation/deoxygenation steps, rather than as a fully quantified kinetic model established by theory. The corresponding discussion and literature citations have been added to the revised manuscript (Lines 343-345).

4. Likewise, for Lines 307-309, where the authors state that "the asymmetric mode is more readily hydrogenated by a nearby adsorbed H," appropriate literature citations should be included to support this claim.

Response: We thank the reviewer for this careful suggestion.

We thank the reviewer for this careful suggestion. We agree that appropriate literature support should be provided for the statement that the asymmetric adsorption mode may facilitate subsequent hydrogenation.
In the revised manuscript, we have added relevant references and revised the mechanistic discussion more cautiously. According to previous studies, NO_3^- has a planar structure with D_{3h} symmetry, in which the three N-O bonds are theoretically equivalent. When hydrogen bonding or interfacial interactions preferentially involve one N-O group, local symmetry breaking can occur, accompanied by transient elongation of the involved N-O bond and charge redistribution within the NO_3^- ion [J. Phys. Chem. A 114, 1255–1269 (2010); J. Chem. Phys. 93, 3379–3388 (1990)].

Based on this understanding, the asymmetric adsorption mode may render a specific oxygen terminus more reactive than in the symmetric adsorption mode, thereby facilitating subsequent hydrogenation/deoxygenation-related steps. To avoid overstatement, we have revised the original sentence from "the asymmetric mode is more readily hydrogenated by a nearby adsorbed H" to a more cautious description: the asymmetric nitrate adsorption configuration may promote local N-O bond activation and facilitate subsequent hydrogenation/deoxygenation steps.

Moreover, *in-situ* ATR-FTIR and electrochemical results provide consistent experimental support for this interpretation. The increased fraction of asymmetric nitrate-related species, reflected by the v_{as}/v_s ratio, correlates with the enhanced NH_3 selectivity and yield rate over Fe-N_3(PO_X), supporting the catalytic relevance of asymmetric adsorption configurations in NO_3RR. The corresponding explanation and literature citations have been added to the revised manuscript (Lines 343-345).

5. The applied potentials should be clearly indicated in the *in-situ* FTIR data to facilitate direct comparison with other *in-situ* measurements. Currently, the potential intervals are only provided in the table and manuscript text. Please clearly label the corresponding potentials in both the figure and the Methods section.

Response: We thank the reviewer for this helpful suggestion.

To improve clarity and facilitate direct comparison with the other *in-situ* measurements, we have revised the *in-situ* FTIR figure to clearly indicate the corresponding applied potentials. We have also added the relevant potential information more explicitly in the Methods section. These revisions have been made in the

updated figure and the corresponding experimental description in the revised manuscript (Fig 4a, b and Supplementary Fig. 22).

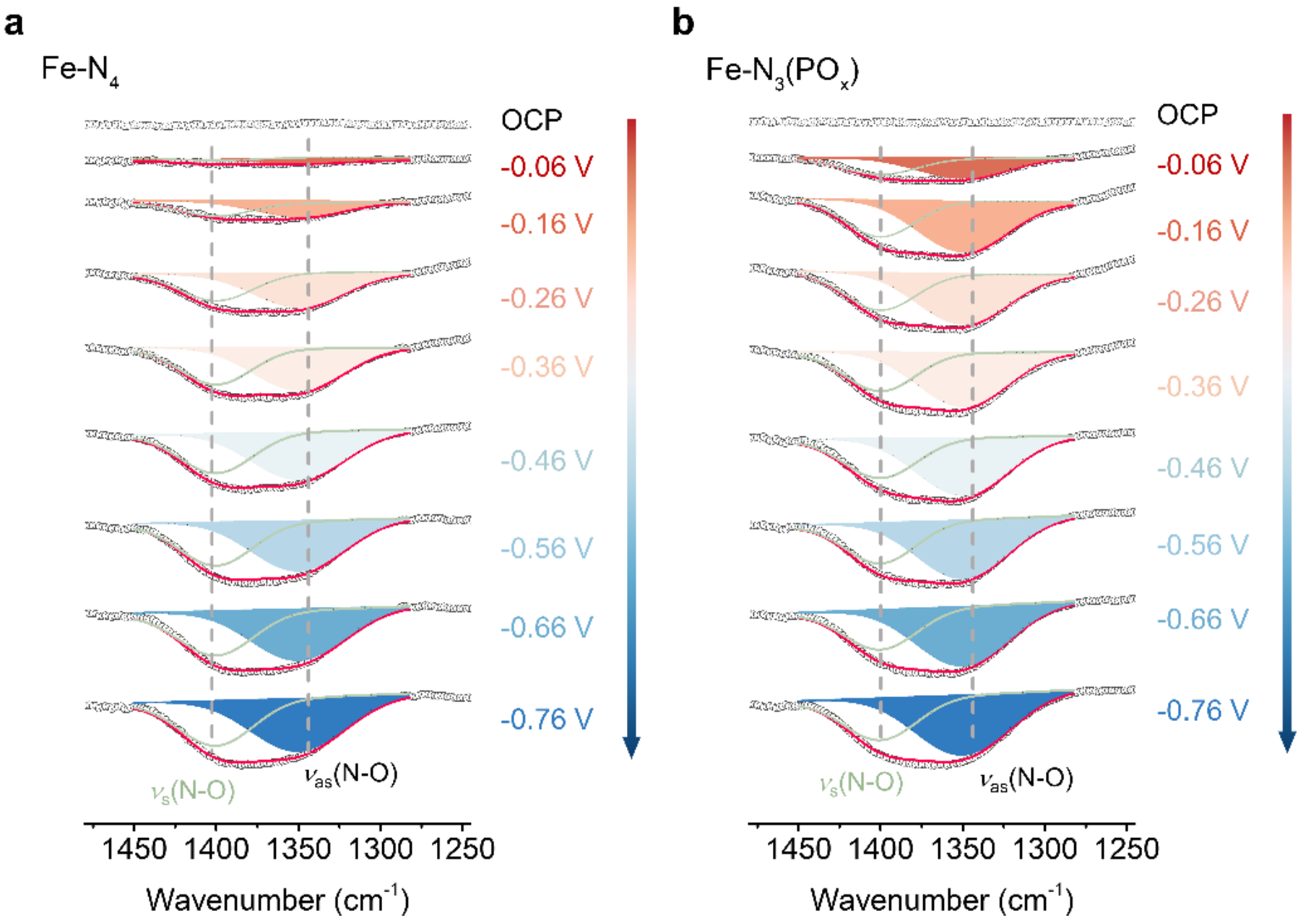

Supplementary Fig. 22. *In-situ* ATR-FTIR fitting spectra for a Fe-N_4 and b Fe-$N_3(PO_X)$. The NO_3^- stretching bands are deconvoluted into symmetric and asymmetric peak.

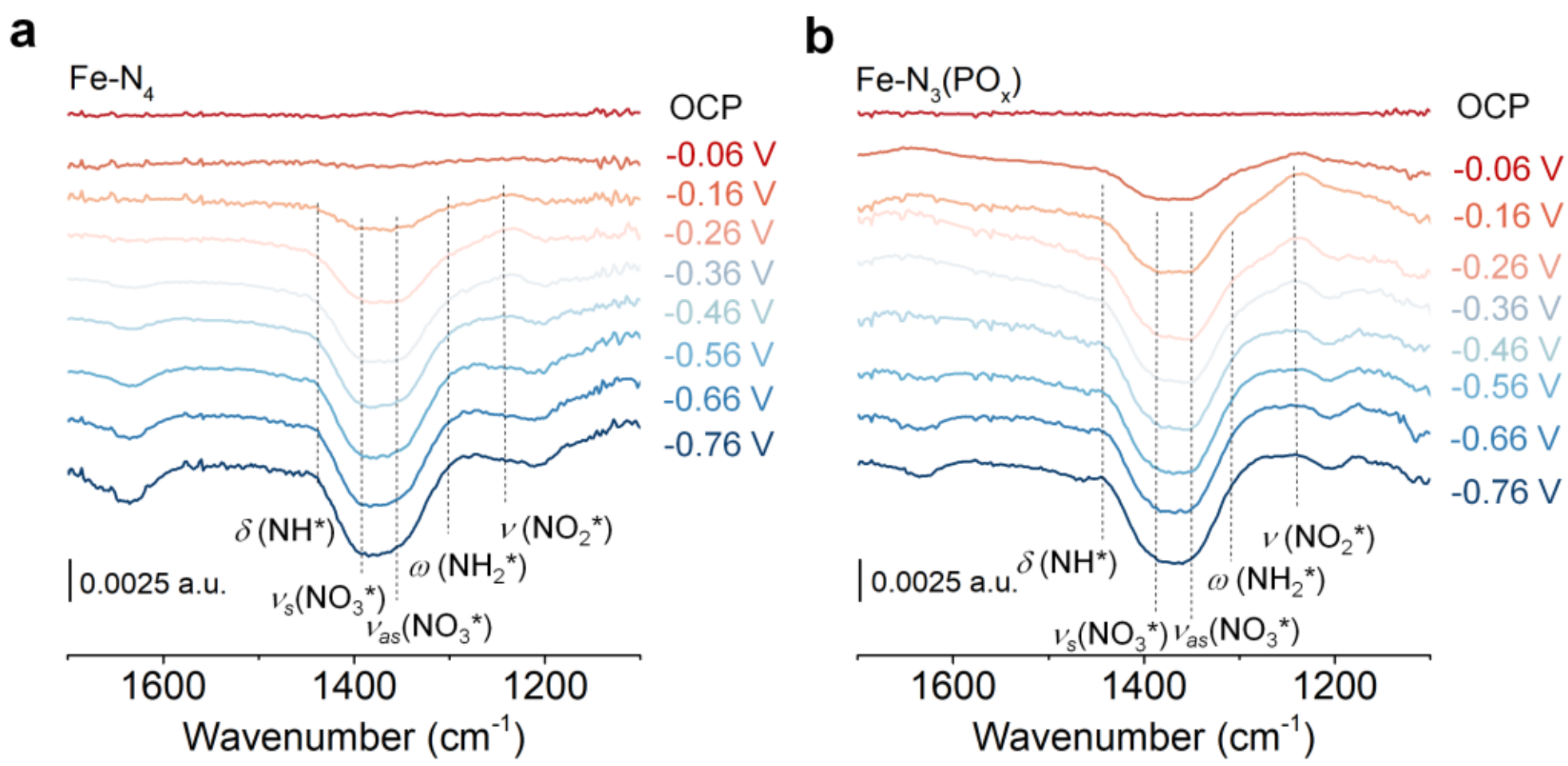

Fig. 4 | *In-situ* ATR-FTIR and XES mechanistic study of NO_3RR. *In-situ* ATR-FTIR spectra of **a** Fe-N_4 and **b** Fe-$N_3(PO_X)$ during potential scan from OCP to -0.76 V.

Thank you for the reviewers' constructive comments. We have revised the manuscript to improve its scientific rigor and clarity.

Response to Decision Letter
Manuscript ID: NCOMMS-26-006178A

We sincerely thank the editor and reviewer for their careful evaluation of our manuscript and for the constructive comments. In response to the remaining concerns, we have substantially revised the manuscript and provided additional evidence to strengthen the mechanistic interpretation and practical relevance of our work. Specifically, we have supplemented theoretical calculations to clarify the structure-activity relationship and the proposed reaction pathway, and we have further included a practical demonstration expected for application-driven NO_3RR studies. The detailed responses to the comments and the corresponding revisions are provided below.

Reviewer #2:
This manuscript has been revised in response to our previous comments. However, some comments are still not replied directly and the central arguments were not salient, despite the heavy workload, including:

(i) No solid proof can prove the proposed reaction pathway for the NO_3RR. Particularly, the insignificant *In-situ* ATR-FTIR signals cannot be assigned to the intermediate species, which is an equivocal inference. Although the authors reposition the manuscript as a mechanistic study, the mechanistic understanding provided remains insufficient. In NO_3RR, the identification of key reaction intermediates and dominant reaction pathways represents the most fundamental basis for mechanistic interpretation. However, these issues are still not clearly resolved in the present work. While *operando* characterizations of catalyst reconstruction are provided, the manuscript mainly focuses on structural evolution itself, without convincingly establishing how such reconstruction directly governs the catalytic pathway or activity. As a result, the proposed structure–activity relationship and mechanistic conclusions remain largely speculative and are not yet adequately supported by direct experimental or theoretical evidence.

Response: We thank the reviewer for this important suggestion.

We agree that identifying key reaction intermediates, dominant reaction pathways and realistic working-state configurations is essential for establishing a reliable mechanistic interpretation of NO_3RR. To address this concern, we have added systematic DFT calculations in the revised manuscript and revised the mechanistic discussion to more directly connect the experimentally observed catalyst reconstruction with the catalytic pathway and activity (**Fig. 5**). Specifically, Fe-N_3(PO_X), Fe-N_2(PO_X) and Fe-N_2 were constructed as representative working-state configurations based on the *in-situ* XAS results to describe the progressive coordination evolution of the Fe center during NO_3RR. These models represent successive reconstructed states of the same Fe active center under reaction conditions, highlighting the potential-driven coordination reconstruction process. Their optimized atomic structures are provided in **Supplementary Fig. 31**. The optimized adsorption configurations of H and key nitrate-derived intermediates are provided in **Supplementary Figs. 32-35. Supplementary Table 10** summarizes the zero-point energy and entropic corrections for key adsorbed intermediates involved in the nitrate reduction reaction.

The calculated formation energies (**Fig. 5a**) of Fe-N_2, Fe-N_2(PO_X) and Fe-N_3(PO_X) are -0.32, -0.68 and -0.94 eV, respectively, indicating that PO_X stabilizes the asymmetric Fe coordination environment and provides a thermodynamically accessible platform for subsequent potential-driven reconstruction. In addition, the Fe local magnetic moment increases from 2.39 μB for Fe-N_3(PO_X) to 2.83 μB for Fe-N_2(PO_X) and remains

high after NO_3 adsorption (**Fig. 5b**), consistent with the higher-spin character observed by *in-situ* Fe Kβ XES. These results establish a direct connection between the experimentally observed coordination reconstruction and the spin-state evolution of the Fe active center. More importantly, the newly added theoretical results further clarify how different reconstructed coordination states regulate different steps of the NO_3RR pathway. COHP and charge-density-difference analyses show that Fe-$N_3(PO_X)$ exhibits the strongest Fe-NO_3 orbital coupling, indicating its favorable role in initial nitrate adsorption and activation (**Fig. 5c-g**). After partial reconstruction to Fe-$N_2(PO_X)$, the Fe-NO_3 interaction is moderated while effective electronic coupling is retained. In addition, Fe-$N_2(PO_X)$ shows an intermediate H adsorption free energy (**Fig. 5h**) and substantially lowers the energy requirement for the NO* to NOH* hydrogenation step from 2.11 eV on Fe-$N_3(PO_X)$ and 1.48 eV on Fe-N_2 to 0.69 eV (**Fig. 5i**). These results indicate that Fe-$N_2(PO_X)$ provides a balanced local environment for the hydrogenation of nitrate-derived intermediates, while the further reconstructed Fe-N_2 configuration favors downstream conversion of N-containing intermediates toward NH_3.

Therefore, PO_X-assisted coordination reconstruction enables the Fe active center to form continuous working-state configurations with complementary catalytic functions during NO_3RR, thereby integrating nitrate activation, intermediate hydrogenation and NH_3 formation into a dynamic structural-evolution framework (**Lines 396-432**). We sincerely hope that these revisions address the reviewer's concern and provide a more solid mechanistic basis for the proposed NO_3RR pathway and structure-activity relationship.

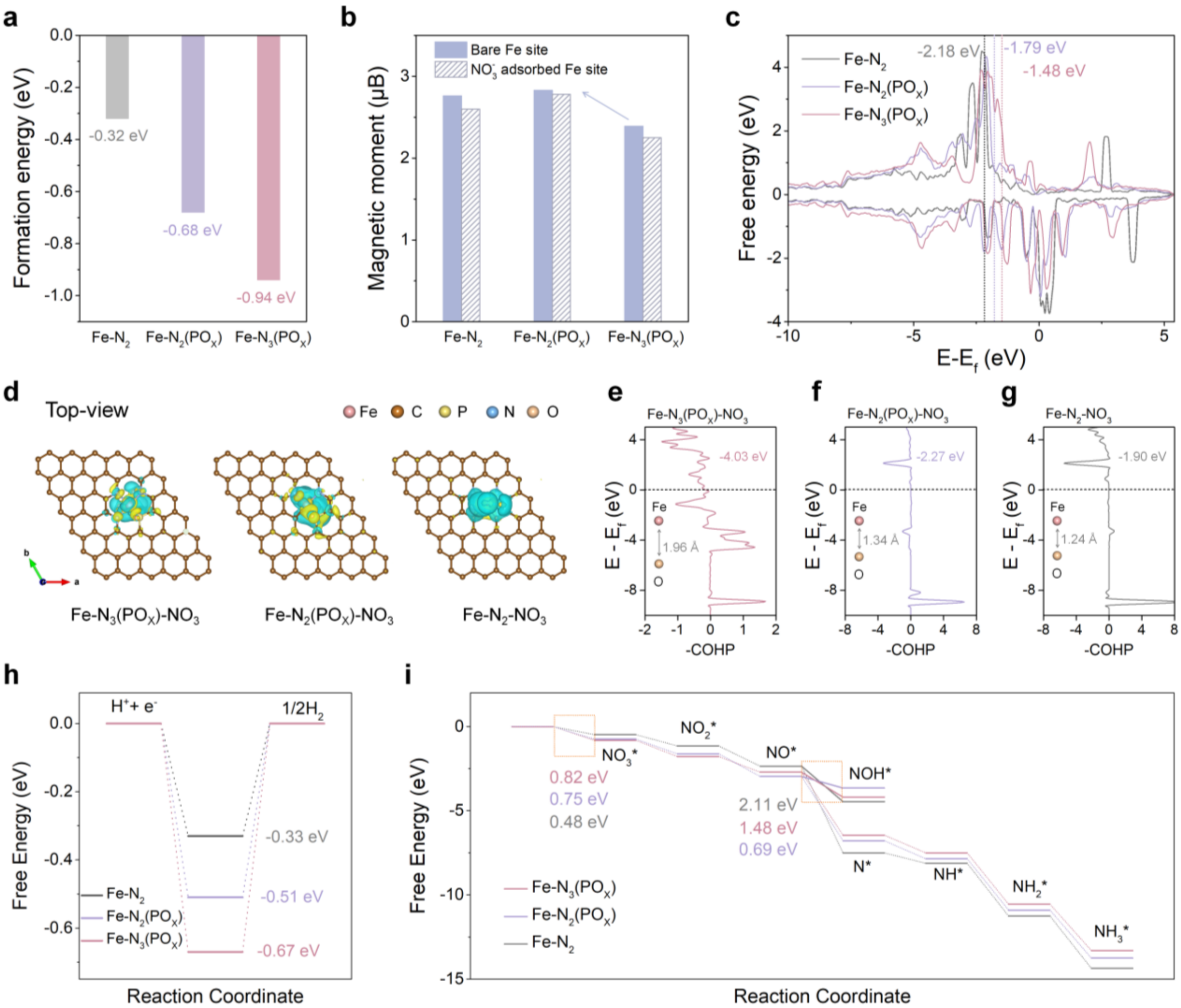

Fig. 5 | Theoretical investigation of the reconstructed active configuration for NO_3RR.

a Formation energy of Fe-N_2, Fe-$N_2(PO_X)$ and Fe-$N_3(PO_X)$ models**. b** Magnetic moments of the three models with before and after NO_3^- adsorption. **c** Fe 3*d* projected density of states and *d*-band center. **d** Charge density

difference of three NO_3^--adsorbed models. COHP analyses of Fe-O interactions in **e** Fe-$N_3(PO_X)$, **f** Fe-$NN_2(PO_X)$ and **g** Fe-N_2. **h** Gibbs free energies of H adsorption of Fe-N_2, Fe-$N_2(PO_X)$ and Fe-$N_3(PO_X)$. **i** Gibbs free-energy diagrams for the NO_3RR pathway over the three models.

(ii) The authors attribute the absence of theoretical calculations to the dynamic coordination reconstruction of Fe single-atom sites under NO_3RR conditions. However, this argument is not sufficiently convincing and instead exposes a more fundamental issue in the current mechanistic interpretation. If the catalyst structure is continuously evolving under operating conditions, this directly raises concerns regarding the structural stability and identity of the true active sites. More importantly, the existence of reconstruction cannot be used as a justification for avoiding theoretical analysis. On the contrary, if the reconstructed configuration is proposed to be the actual active state, then representative working-state models should be established based on the *operando* characterization results, followed by corresponding theoretical calculations to clarify the structure–activity relationship and reaction pathway.

Response: We thank the reviewer for this important suggestion.

We agree that dynamic coordination reconstruction should not be used as a reason for the absence of theoretical analysis. Instead, the reconstruction process should be incorporated into representative working-state models based on *in-situ* characterization results to clarify the catalytically relevant active configurations, the structure-activity relationship and the NO_3RR reaction pathway. In the revised manuscript, Fe-$N_3(PO_X)$, Fe-$N_2(PO_X)$ and Fe-N_2 were constructed as representative working-state configurations based on the *in-situ* XAS results to describe the progressive coordination evolution of the Fe center under NO_3RR conditions. As partly discussed in our response to **Comment (i)**, we have added systematic DFT calculations to strengthen the mechanistic interpretation of NO_3RR and to establish a more direct connection between the experimentally observed coordination reconstruction and the catalytic pathway (**Fig. 5**). These configurations represent successive reconstructed states of the same Fe active center under reaction conditions, rather than three independent static active sites (**Lines 396-432**).

The calculated results show that PO_X stabilizes the asymmetric Fe coordination environment and promotes potential-driven coordination reconstruction toward low-coordinated Fe working states. Meanwhile, the increase in Fe local magnetic moment from Fe-$N_3(PO_X)$ to Fe-$N_2(PO_X)$, together with the higher-spin character observed by *in-situ* Fe Kβ XES, supports the formation of a spin-polarized working state during NO_3RR. The theoretical results further show that these reconstructed coordination states regulate nitrate adsorption, Fe-NO_3 orbital coupling, H adsorption and the hydrogenation of nitrate-derived intermediates. In particular, Fe-$N_2(PO_X)$ retains effective Fe-NO_3 electronic coupling while lowering the energy requirement for the NO* to NOH* hydrogenation step, thereby providing a more balanced local reaction environment. The further reconstructed Fe-N_2 configuration then favors downstream conversion of N-containing intermediates toward NH_3 (**Fig. 5**).

Therefore, the revised manuscript no longer treats dynamic coordination reconstruction as an unresolved structural complexity. Instead, it incorporates the reconstruction process into a working-state mechanistic framework. This revised interpretation integrates *in-situ* observed active-site reconstruction, spin-state enhancement, reaction-intermediate energetics and catalytic activity within a unified structure-activity framework. We sincerely hope that the newly added theoretical calculations and revised discussion address the reviewer's concern and provide a more solid mechanistic basis for the proposed NO_3RR pathway.

(iii) Repositioning the manuscript as a "mechanistically informative model catalyst platform" does not fully address the original concern. For a study targeting a high-impact journal such as Nature Communications, application-oriented evaluations are not merely optional additions, but are increasingly expected to demonstrate the broader significance and practical relevance of the proposed catalyst system. Simply acknowledging that the current density is insufficient for practical operation does not eliminate the need for meaningful application-level assessment. More importantly, the manuscript now attempts to shift its emphasis entirely toward mechanistic understanding. However, as discussed above, the mechanistic insights themselves remain insufficiently established, since key reaction intermediates, dominant pathways, and realistic active-site configurations have not been conclusively identified. Therefore, the work currently lacks both the practical demonstration expected for application-driven NO_3RR studies and the mechanistic rigor required for a fundamentally oriented study. In conclusion, I still can't recommend the publication of this manuscript on Nature Communications.

Response: We sincerely thank the reviewer for this important and constructive comment

We fully agree that, for an application-oriented NO_3RR study, both practical relevance and mechanistic rigor are essential. Accordingly, in the revised manuscript, we have added flow-cell experiments and systematic theoretical calculations to address the reviewer's concerns from both the application and mechanistic perspectives.

First, to strengthen the application-level assessment, Fe-N_3(PO_X) was assembled in a flow-cell configuration using an FAA-130 alkaline anion-exchange membrane, and nitrate reduction measurements were performed under continuous-flow conditions. The corresponding activity and stability results have been added to the revised manuscript. Fe-N_3(PO_X) delivered a current density of 0.15 A cm^{-2} at a cell voltage of 2.5 V with a Faradaic efficiency of 85%, and maintained stable operation for 20 h. The NH_3 yield rate and Faradaic efficiency before and after the stability test showed no obvious change, further indicating the stability of the catalyst during flow-cell operation (**Supplementary Fig. 30**). These results demonstrate that Fe-N_3(PO_X) can sustain nitrate-to-ammonia conversion in a membrane-based flow-cell system with improved mass transport. The relevant discussion has been added to the revised main text (**Lines 253-265**), and the detailed experimental procedure has been included in the Methods section (**Lines 509-520**). These additional results provide an application-relevant assessment beyond conventional H-cell measurements and support the practical application potential of the Fe-N_3(PO_X) catalyst system.

Second, to reinforce the mechanistic interpretation, we have added systematic DFT calculations to evaluate nitrate activation, H adsorption, key hydrogenation steps and the energetic evolution of representative NO3RR intermediates on Fe-N_3(PO_X)-derived working-state configurations. As described in our responses to **Comments (i) and (ii)**, Fe-N_3(PO_X), Fe-N_2(PO_X) and Fe-N_2 were constructed based on the *in-situ* XAS results to describe the progressive coordination evolution of the Fe center during NO_3RR. The calculated results establish a more direct connection between the experimentally observed coordination reconstruction and the catalytic reaction pathway. In particular, the reconstructed Fe-N_2(PO_X) configuration retains effective Fe-NO_3 electronic coupling while substantially lowering the energy requirement for the NO* to NOH* hydrogenation step, thereby providing a favorable local environment for nitrate-derived intermediate hydrogenation. These results further support the proposed structure-activity relationship.

Together, these revisions directly address the reviewer's concern that the previous version lacked sufficient application-level assessment and mechanistic rigor. The revised manuscript now combines flow-cell-level nitrate-to-ammonia conversion with a clearer working-state mechanistic framework, linking

coordination reconstruction, spin-state enhancement, reaction-intermediate energetics and catalytic activity. We sincerely hope that the newly added data and revised discussion more clearly demonstrate the broader significance, practical relevance and mechanistic basis of the Fe-$N_3(PO_X)$ catalyst system.

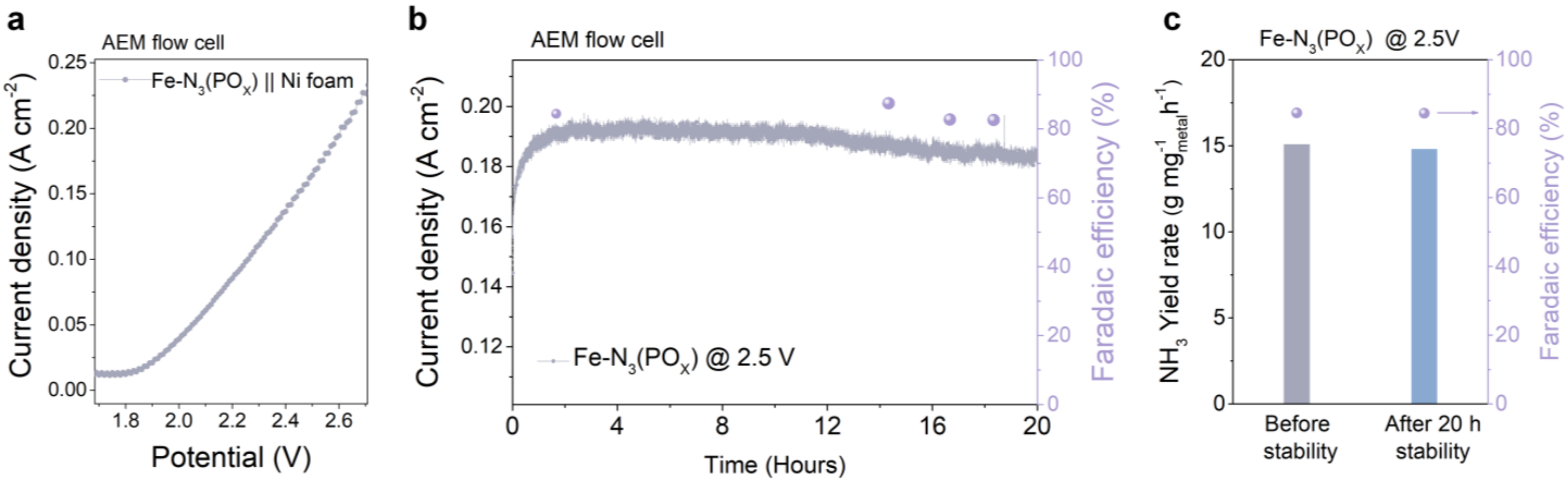

Supplementary Fig. 30. a LSV curve of Fe-$N_3(PO_X)$ measured in an alkaline AEM flow cell. **b** 20 h flow-cell stability test at 2.5 V with corresponding NH_3 Faradaic efficiencies. **c** Comparison of NH_3 yield rate and Faradaic efficiency at 2.5 V before and after the 20 h flow-cell stability test.

We sincerely thank the reviewer for their continued evaluation of our manuscript. We have carefully addressed the remaining concerns and further revised the manuscript to improve its scientific rigor, clarity, and evidentiary support.

Response to Referees

Manuscript ID: NCOMMS-26-006178B-Z

Dear Referees,

We sincerely appreciate the reviewers' careful evaluation and positive assessment of our revised manuscript. Our responses to the reviewers' comments are provided below.

Reviewer #2:

All comments are addressed.

Response: We appreciate the reviewer's confirmation that the previous comments have been fully addressed.

Reviewer #4:

As a new reviewer for this revised manuscript, I checked the response letter, revised manuscript, and the newly added computational results. This is an excellent work and thus I recommend acceptance in its current form.

Response: We appreciate the reviewer's positive assessment and recommendation for acceptance.

We again thank the Editor and Reviewers for their careful assessment and constructive comments, which have helped us improve the clarity and rigor of the manuscript.
